# Pan-cancer analysis reveals SMARCAL1 expression is associated with immune cell infiltration and poor prognosis in various cancers

**DOI:** 10.1038/s41598-025-88955-9

**Published:** 2025-02-24

**Authors:** Wu-jie Zhao, Meng-lei Wang, Yun-fang Zhao, Wen-peng Zhao, Qiong-hui Huang, Zhen-wei Lu, Fang Jia, Jin-jin Shi, Bo-sen Liu, Wan-hong Han, Han-wen Lu, Bing-chang Zhang, Zhan-xiang Wang

**Affiliations:** 1 Department of Neurosurgery and Department of Neuroscience, Fujian Key Laboratory of Brain Tumors Diagnosis and Precision Treatment, Xiamen Key Laboratory of Brain Center, The First Affiliated Hospital of Xiamen University, School of Medicine, Xiamen University, Xiamen, 361005 Fujian China; 2 Department of Digestive Diseases, The First Affiliated Hospital of Xiamen University, School of Medicine, Xiamen University, Xiamen, 361005 Fujian China; 3 Jitang College of North China University of Science and Technology, Tangshan, 063000 Hebei China; 4 The School of Clinical Medicine, Fujian Medical University, Fuzhou, 350108 Fujian China; 5 Department of Neurosurgery, The Third Affiliated Hospital of Sun Yat-Sen University, Guangzhou, 510630 Guangdong China

**Keywords:** SMARCAL1, Pan-cancer, Glioma, Immunotherapy, Integrative analysis, Cancer, Computational biology and bioinformatics

## Abstract

Although immune checkpoint inhibition in particular has shown promise in cancer immunotherapy, it is not always efficient. Recent studies suggest that SMARCAL1 may play a role in tumor immune evasion, yet its pan-cancer role is unclear. We conducted a comprehensive analysis of SMARCAL1 using TCGA, GTEx, and CCLE databases, evaluating its expression, genetic alterations, epigenetic modifications, and their clinical correlations across 33 cancer types. Our findings indicate that SMARCAL1 is overexpressed in several cancers, such as Glioma, LUAD, KIRC, and LIHC, impacting prognosis. Elevated SMARCAL1 is linked to poor outcomes in Glioma, LUAD, and LIHC but correlates with better survival in KIRC. We also found significant associations between SMARCAL1 expression and DNA methylation in 13 cancers. Furthermore, SMARCAL1 expression correlates with immune infiltration, suggesting it as a potential therapeutic target in cancer immunotherapy. This study underscores the need for further research on SMARCAL1 to enhance immunotherapeutic strategies.

## Introduction

Cancer continues to be the world’s greatest cause of death, posing serious threats to life expectancy and public health. Patients suffer significant physical and financial consequences from cancer, and recent research has shown that the prevalence and death of cancer are quickly rising globally^1,2^. The five-year survival rate is still poor despite attempts to enhance clinical outcomes and quality of life, leading to the need for the investigation of innovative treatment approaches^3,4^.

Immunotherapy, utilizing drugs targeting immune checkpoint genes (ICGs), has emerged as a critical approach to cancer treatment. Immune checkpoint inhibitors, such as those targeting PD-1 or PD-L1, have shown efficacy in the treatment of advanced melanoma by inhibiting pathways that shield tumor cells from immune system attack^5,6^. Even while immunotherapy can cause long-lasting remissions in a variety of cancer types, many people still do not benefit from it^7^. Therefore, there is a pressing need for alternative strategies to enhance its efficacy^8,9^.

Recent targeted screening efforts have identified the DNA translocase SMARCAL1 as a factor inhibiting the immune response of tumor cells due to its dual role in restricting innate immune signaling and upregulating PD-L1 levels^10^. SMARCAL1 functions as a DNA helicase, playing a crucial role in DNA replication and repair processes. It unwinds DNA double strands, facilitating DNA rearrangement and repair^11^. The function of SMARCAL1 is essential for maintaining genome stability and preventing DNA damage^12^. Aberrant SMARCAL1 function may impede DNA damage repair, thereby increasing cellular susceptibility and potentially leading to the onset of diseases such as immunodeficiency and DNA repair-deficient disorders^13^. More research is needed to fully understand the impact of systemic therapeutic SMARCAL1 suppression on host immune cells, especially in light of possible negative feedback against anti-tumor immunity.

This study aims to explore the expression characteristics of SMARCAL1 in pan-cancer, its prognostic value, and its relationship with immunotherapy. To achieve this objective, we obtained patients’ gene expression profiles from public databases, assessing their prognostic value, methylation levels, and potential associations between SMARCAL1 expression and immune-related functions. Additionally, we examined the levels of SMARCAL1 in patients exhibiting different responses to immunotherapy, elucidating its potential role in immunotherapy.

## Methods

### SMARCAL1 expression and subcellular localization analysis

Using information from the Human Protein Atlas (HPA, https://www.proteinatlas.org/), we investigated the immunohistochemical staining of SMARCAL1 and used immunofluorescence staining images of three human cancer cell lines (HDLM-2, HEK293, and U2OS) to show where SMARCAL1 is subcellularly located in cancer cells. The expression of SMARCAL1 mRNA in both normal and malignant tissues was determined using information from CCLE (https://sites.broadinstitute.org/ccle/)^14^ and UCSC XENA (https://xena.ucsc.edu/)^15^. The R software’s ggplot2.0 package was used to create graphical representations of the analysis results.

### Prediction of prognostic potential of SMARCAL1 in pan-cancer

The predictive potential of SMARCAL1 in pan-cancer was examined utilizing the R packages survivin and survminer, and the Kaplan–Meier technique. Survival data were gathered from the UCSC XENA (https://xena.ucsc.edu/)^15^ and CGGA (http://www.cgga.org.cn/)^16^ databases, and overall survival (OS) was assessed. Single-factor and multi-factor Cox regression analyses were conducted using SPSS v26 to analyze the association between SMARCAL1 and survival, displaying p-values, hazard ratios (HR), and 95% confidence intervals (CI).

### Genetic alteration and DNA methylation analysis

The cBioPortal database (https://www.cbioportal.org/)^17^ was utilized to acquire SMARCAL1 gene alteration profiles across all 32 cancer types from a cohort of 10,953 patients encompassing 10,967 samples. Alterations in SMARCAL1 included Mutation Amplification and Deep Deletion. The mutation datasets were acquired from the publicly available TCGA database (https://portal.gdc.cancer.gov/). Subsequent analyses were conducted using the maftools package in the R software. The promoter DNA methylation levels of SMARCAL1 in both normal and pan-cancer tissues were investigated using UALCAN (http://ualcan.path.uab.edu/analysis.html)^18,19^. The amount of DNA methylation is indicated by the beta value.

### Correlation analysis between SMARCAL1 expression and immune pathways

Publicly available data for 33 human cancers from The Cancer Genome Atlas (TCGA) via the UCSC Xena website (https://xena.ucsc.edu/)^15^ were accessed. Gene Set Variation Analysis (GSVA) using the GSVA package evaluated immune infiltration within the gene expression dataset. Furthermore, the ESTIMATE package estimated the levels of stromal/immune cell infiltration in tumor tissues, utilizing pre-screened stromal/immune-related gene sets. Subsequently, Pearson correlation analysis explored the relationship between SMARCAL1 expression and immune pathways.

### Analysis of SMARCAL1 expression and its relationship with tumor mutation burden (TMB)

Data for cancer types were downloaded from the UCSC Xena website (https://xena.ucsc.edu/)^15^. TMB was calculated for each tumor tissue accordingly, and their correlation was assessed using the Pearson method. R software version 4.0.2 was used for the statistical study, and a p-value of less than 0.05 was deemed statistically significant.

### Analysis of immunogenicity

Information on tumor samples’ immunophenotype scoring (IPS) was sourced from The Cancer Immune Atlas (TCIA, https://dev.cancerimagingarchive.net/) database. The Wilcoxon test was used to assess sample sensitivity to PD1/PDL1 and CTLA4 antibodies by comparing low- and high-SMARCAL1 groups.

### Genes co-expressed with SMARCAL1 and functional analysis

Genes showing positive and negative co-expression with SMARCAL1 were identified from UCSC Xena data (|cor|> 0.3, P < 0.05). The list of genes most correlated with SMARCAL1 or characteristics of cell clusters was uploaded to the Database for Annotation, Visualization, and Integrated Discovery (DAVID, v6.8)^20,21^ for annotation, visualization, and integrated discovery. Official gene symbols were selected as identifiers, with Homo sapiens chosen as the species. Ultimately, the Kyoto Encyclopedia of Genes and Genomes (KEGG) pathway analysis and Gene Ontology (GO) analysis enrichment results were acquired^22–24^.

### Cell culture

293 T cells, human glioma cell lines (U-118 MG, U-87 MG, A172), and the human astrocyte cell line (NHA) were cultured in Dulbecco’s Modified Eagle’s Medium (#SH30243.01, HyClone) supplemented with 10% fetal bovine serum (# WS500T, ABW) and 1% penicillin/streptomycin (# 15140-122, Gibco). Human lung cancer cell lines (PC9 and HCC827) were cultured in RPMI 1640 medium (#C11875500BT, Gibco) with 10% fetal bovine serum and 1% penicillin/streptomycin. All cells were maintained at 37 °C in a humidified atmosphere containing 5% CO₂ to ensure optimal growth conditions.

### Lentiviral packaging and infection

Lentiviral vectors containing shNC, shSMARCAL1#1, and shSMARCAL1#2 were co-transfected with pMDL, pVSVG, and pREV into 293 T cells using Hieff Trans® Liposomal Transfection Reagent (#40802ES08, Yeasen), following the manufacturer’s instructions. Transfected cells were cultured for 48 h, and the supernatant containing viral particles was collected. Cell debris was removed by centrifugation at low speed, followed by filtration through a 0.45 μm sterile filter (#SLHVR33RB, Millipore). For infection, target cells at approximately 40% confluence were incubated with lentiviral particles in the presence of 5 μg/mL Polybrene (#H9268, Sigma-Aldrich). After 24 h, the medium was replaced with fresh culture medium. To establish stable cell lines, cells were selected using Puromycin (#A1113803, Thermo Fisher Scientific) and subsequently used for downstream experiments.

### RNA extraction and real-time quantitative PCR

For total RNA extraction, cells were seeded in 6-multiwell culture plates. The cells were twice washed in PBS once the confluency of the growth plates reached 80–90%. Total RNA was isolated using a total RNA extraction kit (#DP419, TIANGEN) according to the manufacturer’s instructions. All reagents, buffers and containers involving RNA work were RNase-free grade. Using the FastKing RT Kit (with gDNase) (#KR116, TIANGEN), 1 μg of total RNA was reverse-transcribed into cDNA for the real-time quantitative PCR (RT-qPCR) experiment. LightCycler 480 (Roche) was utilized for qPCR tests, and each sample’s cDNA was combined with SuperReal PreMix Plus (SYBR Green) (#FP313, TIANGEN). Using GAPDH as the endogenous control, the expression levels of the treated samples were normalized to the controls and computed using the 2^−ΔΔCT^ formula. The target genes and primers were designed as follows: SMARCAL1 forward 5′-ACACGCAGATCATCGCAGTCAAG-3′ and reverse 5′-AGGCATCCGTTTGGCATCACAG-3′, CD276 forward 5′-GGGCTGTCTGTCTGTCTCATTGC-3′ and reverse 5′- AGCTCCTGCATTCTCCTCCTCAC-3′, GAPDH forward 5′-ACAACTTTGGTATCGTGGAAGG-3′ and reverse 5′-GCCATCACGCCACAGTTTC-3′ (Sangon Biotech).

### Western blotting

The cells were cultured in 6-well tissue culture plates until reaching 80–90% confluence. Following this, the cells were rinsed twice with phosphate-buffered saline (PBS), detached using a cell scraper, and subsequently centrifuged at 15,000 × g at 4 °C. The cell pellets were then washed twice with PBS, resuspended in radioimmunoprecipitation assay (RIPA) lysis buffer (#89,900, Thermo Fisher Scientific) supplemented with a protease inhibitor cocktail, and subjected to ultrasonic treatment on ice. After centrifugation at 15,000 × g at 4 °C for 15 min, the supernatant containing cellular proteins was collected, and the protein concentration was determined using the BCA Protein Detection Kit (#A55860, Thermo Fisher Scientific). Subsequently, 30 μg of protein from each sample was separated by 12% SDS-PAGE and transferred electrophoretically onto a polyvinylidene fluoride (PVDF) membrane. For ease of experimental handling, the membranes were cut into smaller sections prior to antibody hybridization. The membrane was then blocked with 5% skim milk powder in Tris-buffered saline with Tween 20 (TBST) at room temperature for 1 h, followed by overnight incubation at 4 °C with primary antibodies against SMARCAL1 (1:1000, #12,513–1-AP, Proteintech), CD276 (1:1000, #14,453–1-AP, Proteintech), and GAPDH (1:30,000, #60,004–1-IG, Proteintech). After washing, the membrane was incubated with appropriate HRP-conjugated secondary antibodies at room temperature for 60 min. Finally, protein bands were visualized using an enhanced chemiluminescence solution (ECL, New Cell & Molecular Biotech), and band intensities were quantified using Image-J software.

## Result

### SMARCAL1 expression levels and its subcellular localization

**Fig. 1:**
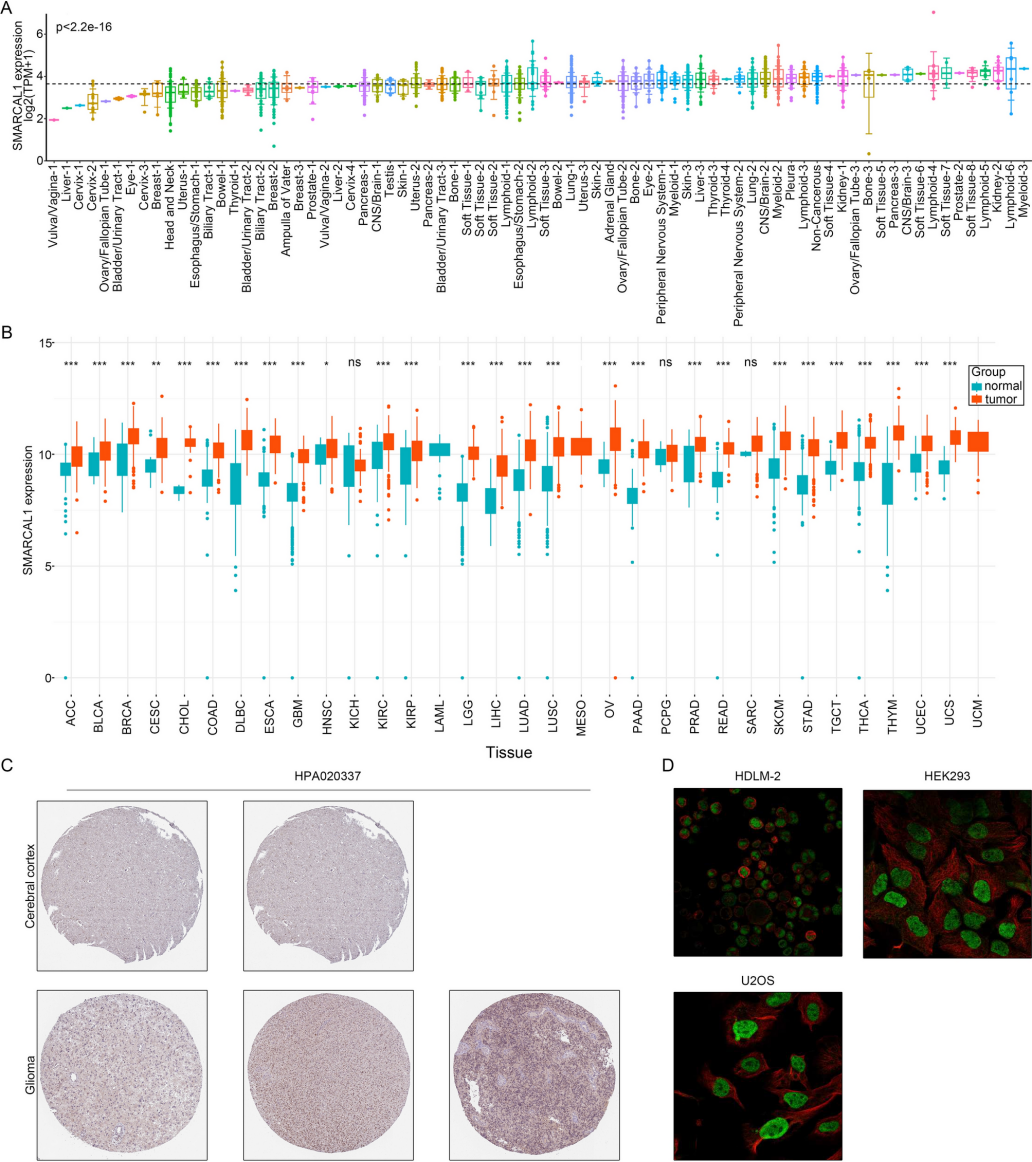
Expression levels and localization of SMARCAL1. (**A**) Expression levels of SMARCAL1 in tumor cell lines based on CCLE datasets. (**B**) SMARCAL1 mRNA expression in TCGA and GTEx datasets comparing cancers to normal tissues. (**C**) The expression of SMARCAL1 in Glioma tissues compared to normal tissues from HPA datasets. (**D**) Subcellular localization of SMARCAL1 in HDLM-2, HEK293, and U2OS cells from HPA datasets. ns, p ≥ 0.05; *p < 0.05; **p < 0.01; ***p < 0.001.

To give a thorough rundown, Additional file 1: Supplementary Table S1 shows the abbreviations for 33 tumors from the TCGA database together with the related association between cell lines and Primary Disease from the CCLE database. Initially, for a comprehensive demonstration of SMARCAL1 expression profiles in tumor and adjacent tissues, we observed elevated levels of SMARCAL1 expression across multiple tumor cell lines, notably in the bowel, uterus, lymphoid, lung, liver, and brain as documented in the CCLE dataset (Fig. 1A). Analysis of the TCGA and GTEx datasets revealed significant upregulation (P < 0.05) of SMARCAL1 in ACC, BLCA, BRCA, CESC, CHOL, DLBC, ESCA, GBM, HNSC, KIRC, KIRP, LGG, LIHC, LUAD, LUSC, OV, PAAD, PRAD, READ, SKCM, STAD, TGCT, THCA, THYM, UCEC, and UCS, compared normal tissue (Fig. 1B). The HPA datasets were employed for comparative analysis of SMARCAL1 protein expression levels across various tumors. Results indicated elevated expression levels of SMARCAL1 in Glioma tissues compared to normal tissues (Fig. 1C), with the protein primarily located within the nucleus (Fig. 1D).

### Association between pan-cancer diagnosis and prognosis and SMARCAL1 expression

**Fig. 2:**
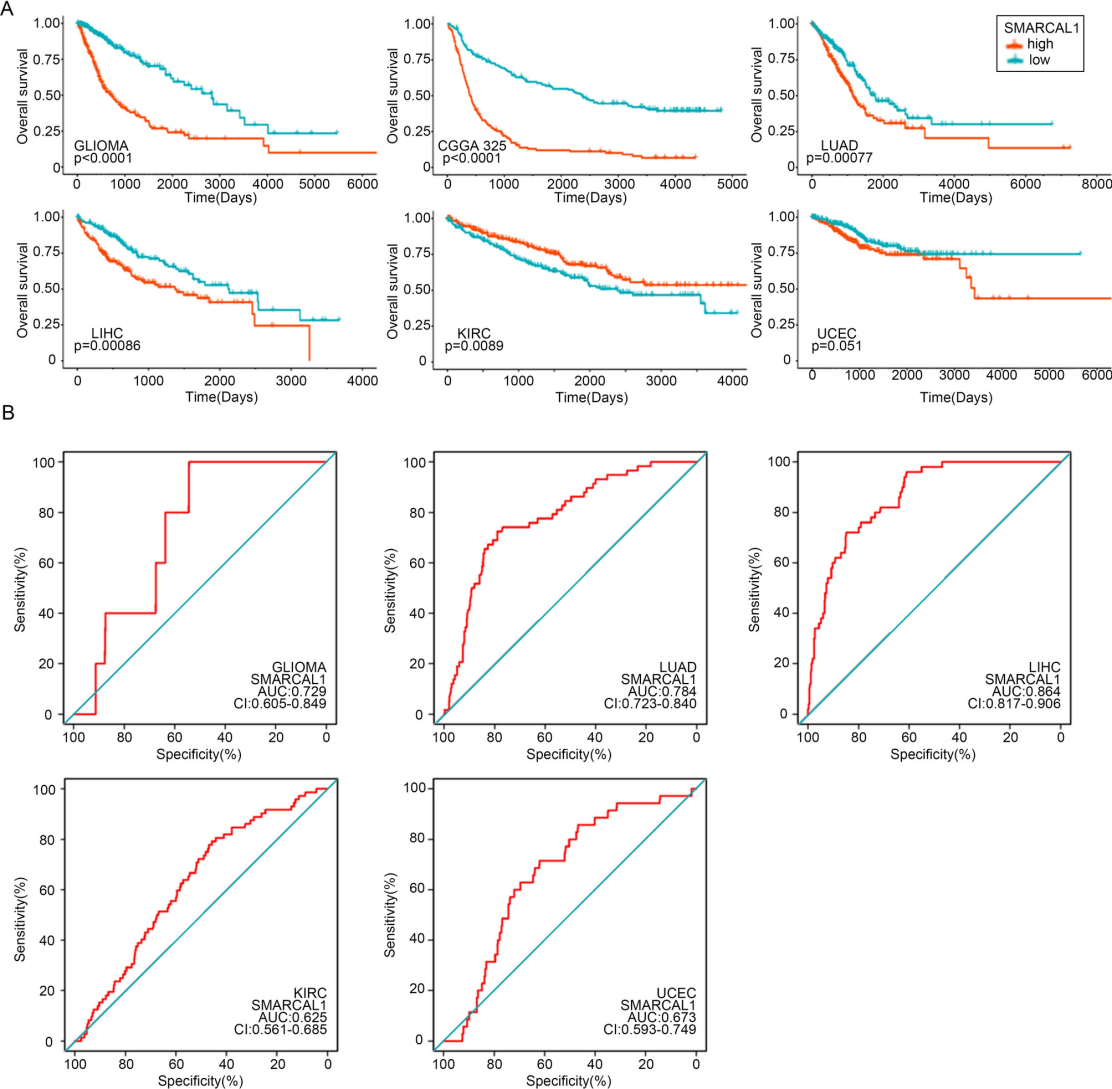
The prognostic role of SMARCAL1 in human pan-cancer. (**A**) OS curves using the Kaplan–Meier approach that contrast high and low expression of SMARCAL1 in various cancer types. (**B**) ROC curve analysis reveals the pan-cancer diagnostic capability of SMARCAL1.

According to the findings of the Cox assessment (Fig. 2A and Additional file 1: Fig. S1), SMARCAL1 is identified as an adverse predictor of overall survival (OS) in Glioma, LUAD, and LIHC (P < 0.05), but exhibits potential as a beneficial factor in KIRC (P < 0.05). Subsequently, the diagnostic performance of SMARCAL1 in Glioma, LUAD, LIHC, KIRC, and UCEC were assessed using ROC curves. SMARCAL1 demonstrated moderate accuracy in predicting Glioma (AUC = 0.729), LUAD (AUC = 0.784), LIHC (AUC = 0.864), KIRC (AUC = 0.625), and UCEC (AUC = 0.673), as illustrated in Fig. 2B. The Cox regression analysis revealed that SMARCAL1 expression was a predictive factor for Glioma that was independent of known prognostic markers such as WHO grade, age at diagnosis, IDH mutation, 1p/19q codeletion, and MGMT promoter methylation. These results showed that in the TCGA and CGGA databases, SMARCAL1 is an independent predictive factor for Glioma (Tables 1, 2 and Additional file 1: Supplementary Table S2). In summary, SMARCAL1 expression holds diagnostic and prognostic significance across various cancers, including Glioma.

**Table 1:** Prognostic factors for Glioma in The Cancer Genome Atlas (TCGA) database: univariate and multivariate study of overall survival (OS).

Variable	Univariate analysis		Multivariate analysis	
	HR(95% CI)	*P*-value	HR(95% CI)	*P*-value
SMARCAL1 expression	6.737(4.283–10.597)	1.52E − 16	1.964(1.132–3.407)	0.016
WHO grade	3.257(1.988–5.336)	2.52E − 38	2.108(1.248–3.559)	0.001
Age	1.075(1.063–1.088)	4.28E − 34	1.054(1.038–1.070)	1.79E − 11
IDH status	0.091(0.064–0.129)	2.18E − 40	0.384(0.219–0.676)	0.001
1p/19q Codel	0.220(0.130–0.375)	2.30E − 08	0.489(0.263–0.909)	0.024
MGMT status	0.312(0.225–0.433)	2.95E − 12	0.889(0.607–1.301)	0.544

**Table 2:** Prognostic factors for Glioma in Chinese Glioma Genome Atlas (CGGA) 325 database: univariate and multivariate study of overall survival (OS).

Variable	Univariate analysis		Multivariate analysis	
	HR(95% CI)	*P*-value	HR(95% CI)	*P*-value
SMARCAL1 expression	1.332(1.259–1.411)	4.92E − 23	1.140(1.0651.221)	1.70E − 04
WHO grade	3.497(2.287–5.348)	3.34E − 27	2.928(1.887–4.542)	2.17E − 09
Age	1.033(1.020–1.046)	4.11E − 07	1.013(1.001–1.026)	0.035
IDH status	0.360(0.272–0.477)	8.87E − 13	1.182(0.839–1.667)	0.339
1p/19q Codel	0.170(0.104–0.277)	1.28E − 12	0.271(0.160–0.458)	1.08E − 06
MGMT status	0.873(0.636–1.100)	0.202		

### Enhanced SMARCAL1 expression in glioma associated with predictive molecular markers of malignancy

**Fig. 3:**
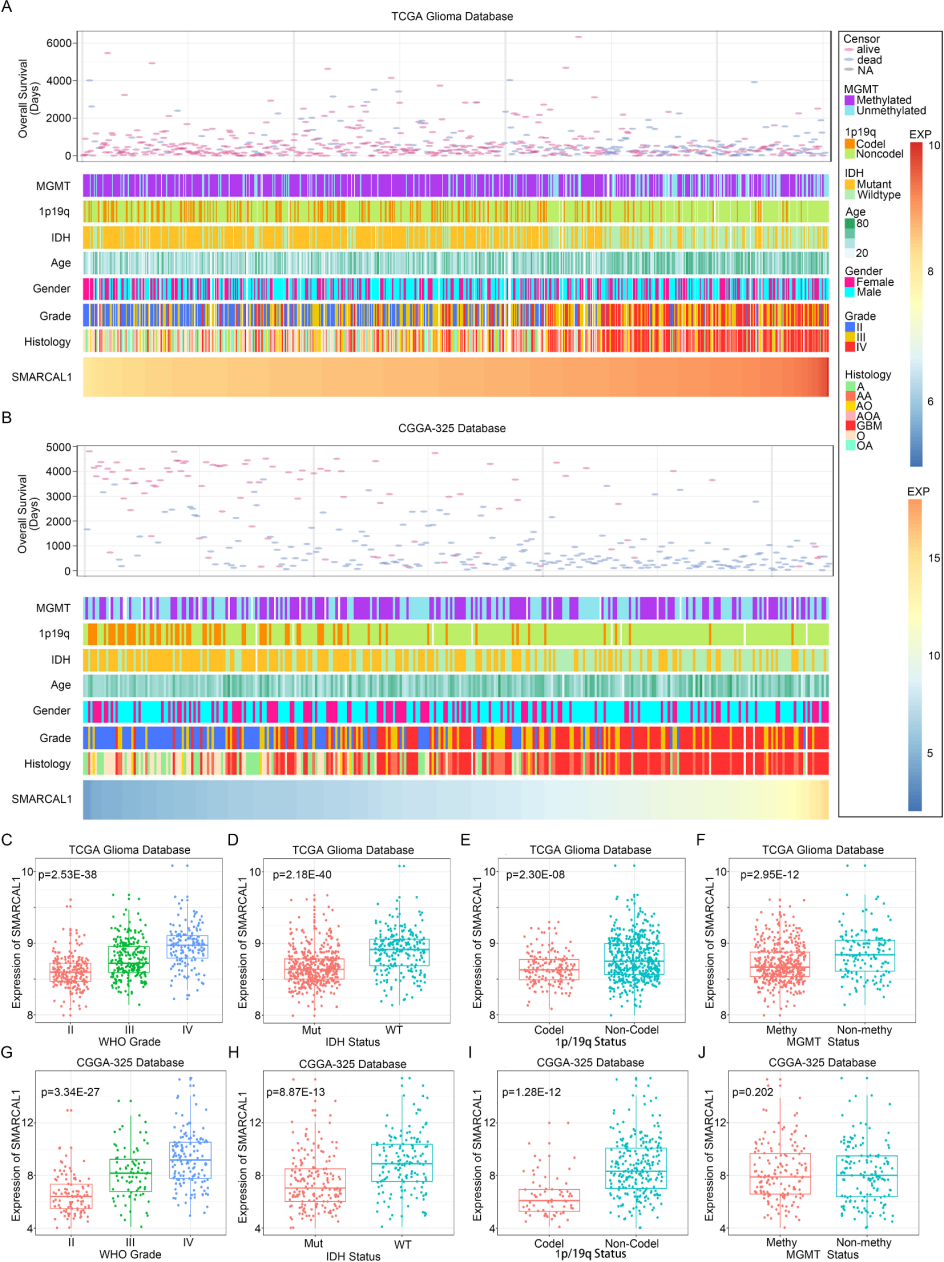
Relationship between the clinicopathological features of Gliomas and SMARCAL1. (**A**) The distribution of clinicopathological characteristics associated with SMARCAL1 in Gliomas within the The Cancer Genome Atlas (TCGA) database. (**B**) The distribution of clinicopathological characteristics associated with SMARCAL1 in Gliomas within the Chinese Glioma Genome Atlas (CGGA) 325 database. (**C**,**G**) The TCGA and CGGA 325 datasets showed a substantial increase in SMARCAL1 in higher-grade Gliomas. One-way ANOVA was used to determine the significance of the difference. (**D**,**H**) The TCGA and CGGA 325 datasets showed a substantial increase in SMARCAL1 in Gliomas without an isocitrate dehydrogenase (IDH) mutation. An unpaired t-test was used to determine the difference’s significance. (**E**,**I**) In the TCGA and CGGA 325 datasets, SMARCAL1 was markedly elevated in Gliomas without 1p/19q codeletion. An unpaired t-test was used to determine the difference’s significance. (**F**,**J**) Increased levels of SMARCAL1 were observed in unmethylated Gliomas with the O6-methylguanine-DNA methyltransferase (MGMT) promoter. Unlike the CGGA 325 database, the TCGA database showed statistical significance for this difference. An unpaired t-test was used to determine the difference’s significance.

Variations in SMARCAL1 expression levels among Glioma patients were found to be linked with distinctive clinical and pathological characteristics. In both the TCGA and CGGA datasets, irregular distributions were seen between increases in SMARCAL1 expression and changes in the IDH mutation status, WHO grade, 1p/19q codeletion status, MGMT promoter methylation status, and histological diagnosis. (Fig. 3A,B and Additional file 1: Fig. S2A). Comparative analyses were conducted across diverse sample cohorts. Particularly noteworthy, within the TCGA database, SMARCAL1 expression exhibited significant increases in higher-grade Gliomas (Fig. 3C) and IDH-wildtype Gliomas (Fig. 3D), as well as in samples lacking 1p/19q codeletion (Fig. 3E). These findings were supported by analyses in the CGGA 325 database (Fig. 3G–I) and CGGA 693 database (Additional file 1: Fig. S3B-D). Moreover, SMARCAL1 expression was markedly higher in samples without MGMT promoter methylation in the TCGA dataset (Fig. 3F) and CGGA 693 dataset (Additional file 1: Fig. S2E). While not statistically significant, a similar tendency in SMARCAL1 expression was noted in the CGGA 325 dataset (Fig. 3J). In summary, our findings indicate that Gliomas characterized by higher malignancy levels tend to exhibit enriched SMARCAL1 expression.

### Epigenetic alterations analysis

**Fig. 4:**
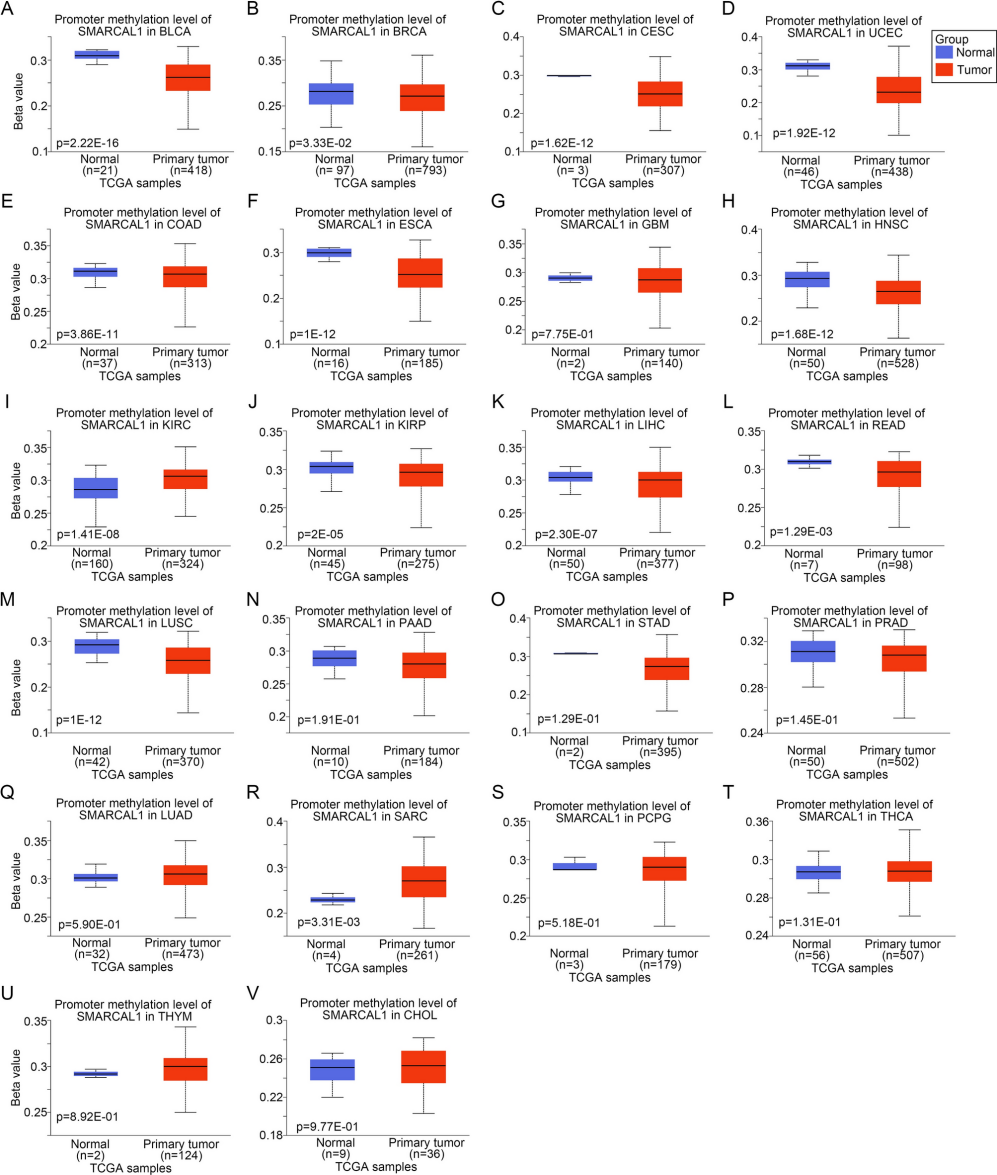
Examining the methylation of DNA in SMARCAL1. (**A**–**V**) SMARCAL1 promoter methylation level in tumor and normal tissues across 22 cancer types from UALCAN.

We investigated 22 different types of cancer tissues as well as normal tissues’ levels of promoter DNA methylation in SMARCAL1. SMARCAL1 exhibited hypomethylation in BLCA, BRCA, CESC, UCEC, COAD, ESCA, GBM, HNSC, PRAD, KIRP, LIHC, READ, LUSC, PAAD, and STAD (Fig. 4A–H and J–P) samples. Conversely, it displayed hypermethylation in KIRC, LUAD, SARC, PCPG, THCA, THYM, and CHOL (Fig. 4I and Q–V) specimens. Although GBM samples showed a trend of hypomethylation, there was no statistically significant difference in SMARCAL1 promoter methylation between tumor and normal groups (Fig. 4G), indicating that other regulatory mechanisms might influence its expression in GBM. SMARCAL1 expression and DNA methylation are closely correlated in a variety of cancer types, according to the reported data.

### Genetic alteration analysis

**Fig. 5:**
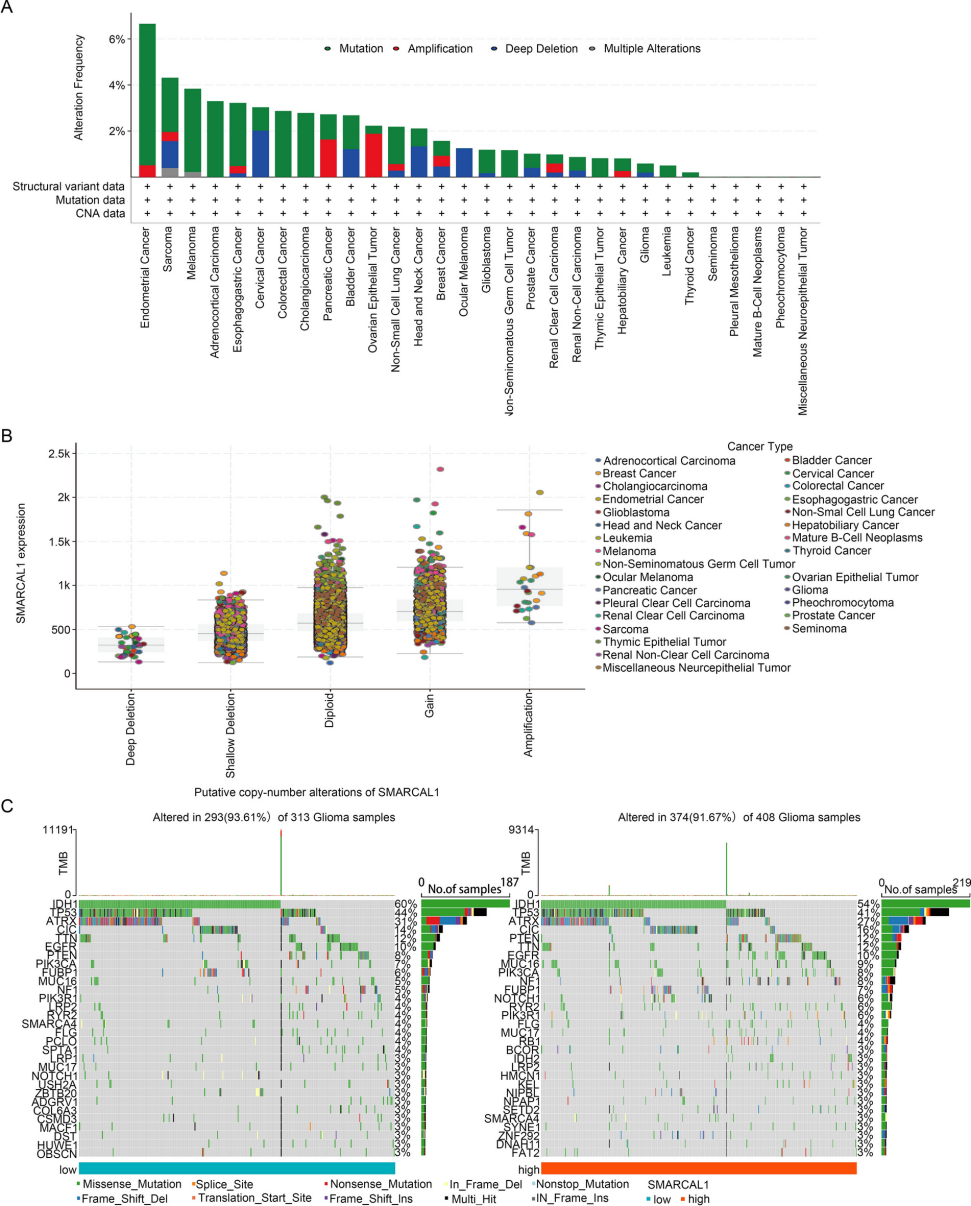
Genetic alteration analysis of SMARCAL1. (**A**) The frequency of changes associated with SMARCAL1 mutation types in various cancer types. (**B**) Pan-cancer tissues’ mRNA expression of SMARCAL1 putative copy-number alteration (CAN). (**C**) The top 30 genes in the The Cancer Genome Atlas (TCGA) database that have the highest frequency of mutations in the low SMARCAL1 expression group and the high SMARCAL1 expression group of Gliomas.

We conducted a comprehensive investigation into pan-cancer genetic alterations in SMARCAL1 utilizing data from the cBioPortal. The mutation rate of SMARCAL1 was examined across 30 different cancer types, as depicted in Fig. 5A. Endometrial Cancer exhibited the highest mutation rate among these types. Predominantly, “mutation” types in copy number variation (CNV) were the most prevalent alterations observed in Glioma. Subsequently, as illustrated in Fig. 5B, we investigated the relationship between SMARCAL1 mRNA expression in pan-cancer tissues and presumed copy-number changes (CNA) in SMARCAL1. The top three types of alterations observed were Shallow Depletion, Diploid, and Gain. In the SMARCAL1 high/low expression cohorts, thirty altered genes were found in the mutation spectrum in Glioma, LUAD, LIHC, KIRC, and UCEC (Fig. 5C and Additional file 1: Fig. S3-4). Notably, among these genes, IDH1, TP53, and ATRX were the top three genes identified in Glioma (Fig. 5C).

### Tumor infiltration analysis

**Fig. 6:**
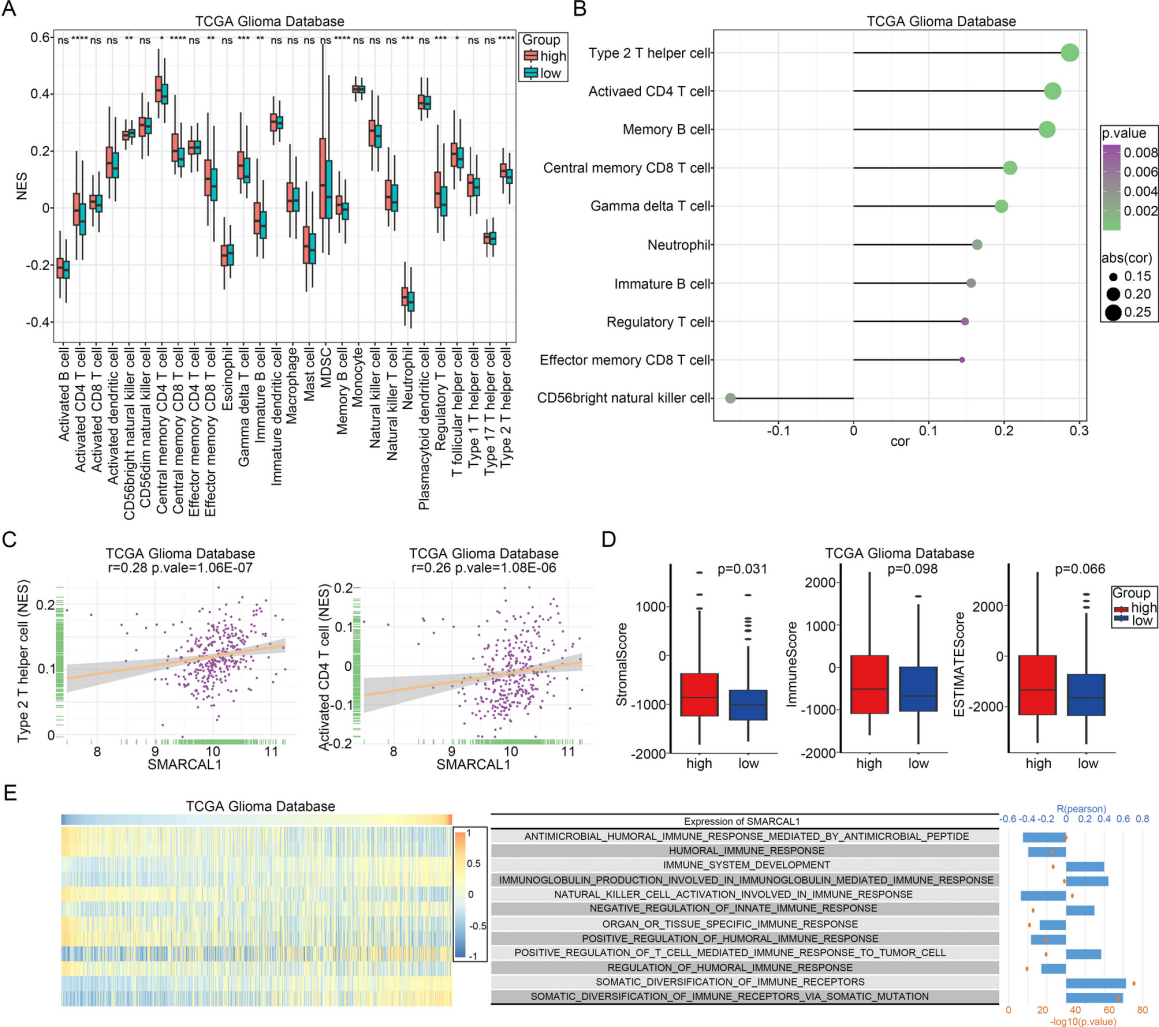
Tumor infiltration analysis of SMARCAL1. (**A**) Relationship between the expression of SMARCAL1 and 28 immune cell types that infiltrate tumors in Gliomas found in The Cancer Genome Atlas (TCGA) database. (**B**) Type 2 T helper cell, Activated CD4 T cell, Memory B cell, Central memory CD8 T cell, Gamma delta T cell, Neutrophil, Immature B cell, Regulatory T cell, and Effector memory CD8 T cell were positively connected with SMARCAL1 expression, while CD56bright natural killer cell was negatively correlated. (**C**) Type 2 T helper cell and Activated CD4 T cell were positively linked with SMARCAL1 expression. (**D**) StromalScore, ImmuneScore, and ESTIMATEScore of SMARCAL1 high and low expression groups of Gliomas in The Cancer Genome Atlas (TCGA) database. (**E**) Each Glioma patient’s SMARCAL1 expression and immunological function enrichment scores were displayed in a heatmap within The Cancer Genome Atlas (TCGA) database. The samples were grouped according to SMARCAL1 expression in ascending order. The correlation analysis’s R- and P-values were displayed in the column and line graphs on the right. ns, p ≥ 0.05; *p < 0.05; **p < 0.01; ***p < 0.001.

**Fig. 7:**
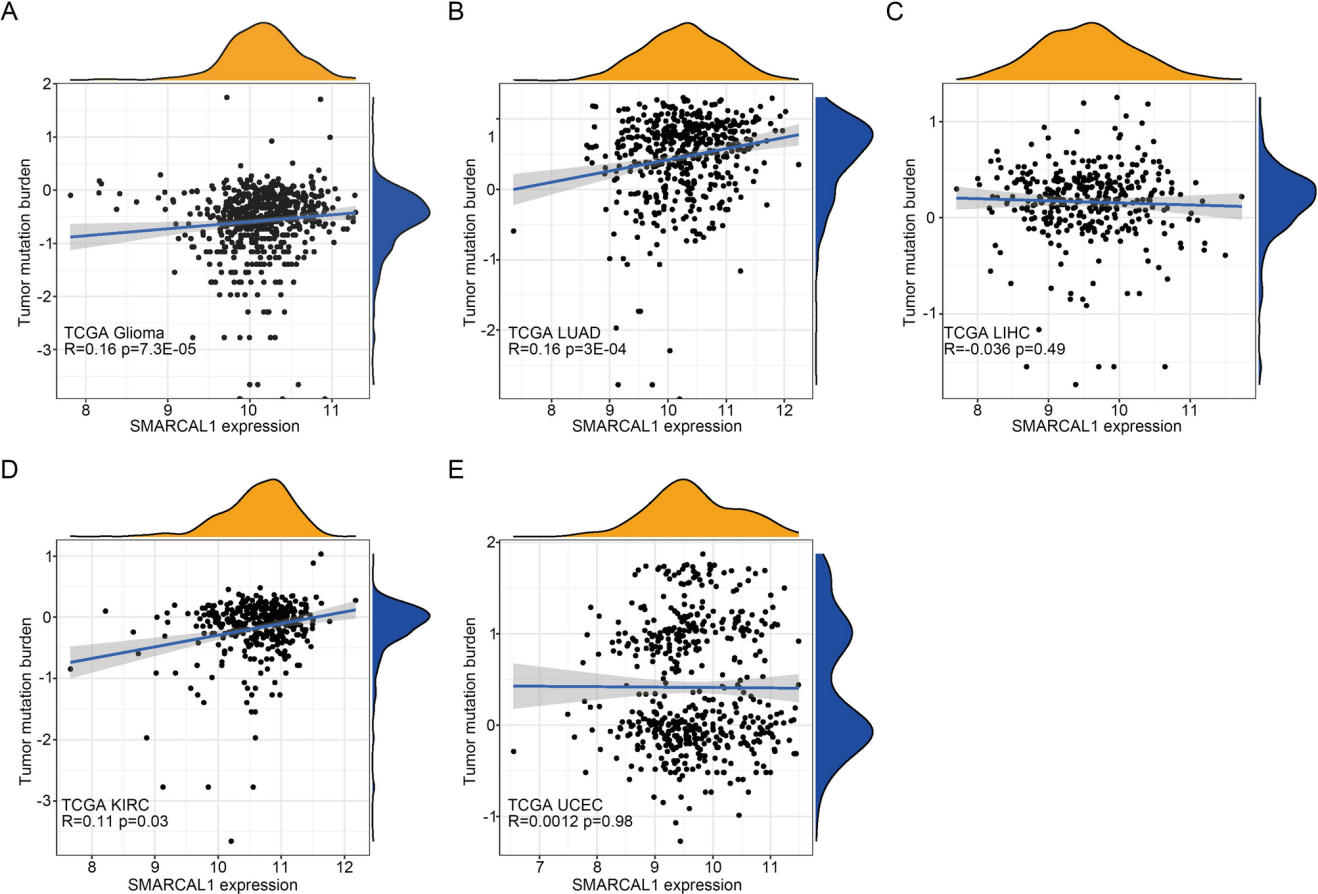
Relationship between SMARCAL1 expression and TMB. (**A**–**E**) SMARCAL1 expression correlation in The Cancer Genome Atlas (TCGA) database with TMB of Glioma, LUAD, LIHC, KIRC, and UCEC.

Tumor-infiltrating lymphocytes are reliable prognostic indicators of cancer survival^25^. Therefore, we investigated the correlation between SMARCAL1 expression and immune infiltration in Glioma, LUAD, LIHC, KIRC, and UCEC (Additional file 1: Supplementary Table S3). As depicted in Fig. 6A and Additional file 1: Fig. S5A-8A, significant relationships were found between SMARCAL1 expression and the degrees of CD4 T cell, CD8 T cell, and T helper cell infiltration in these malignancies. Furthermore, as shown in Fig. 6B and Additional file 1: Fig. S5B-8B, SMARCAL1 demonstrated positive associations with the infiltration levels of Type 2 T helper cells (R = 0.28) and Activated CD4 T cells (R = 0.26) in Glioma (Fig. 6C), Type 2 T helper cells (R = 0.24) in LIHC (Additional file 1: Fig. S6C), Memory B cells (R = 0.33) and Regulatory T cells (R = 0.29) in KIRC (Additional file 1: Fig. S7C), as well as Type 2 T helper cells (R = 0.29) in UCEC (Additional file 1: Fig. S8C). Conversely, negative correlations were observed with the infiltration levels of Eosinophils (R = -0.24) and Mast cells (R = -0.14) in LUAD (Additional file 1: Fig. S5C), Neutrophils (R = -0.34) in LIHC (Additional file 1: Fig. S6C), and Eosinophils (R = -0.31) in UCEC (Additional file 1: Fig. S8C). Higher scores of stromal and immune cells were associated with elevated SMARCAL1 expression in Glioma and KIRC tissues, indicating a strong positive correlation with the ESTIMATE score (Fig. 6D and Additional file 1: Fig. S7D). Conversely, lower scores were observed in LUAD, LIHC, and UCEC specimens (Additional file 1: Fig. S5D, Fig. S6D, and Fig. S8D). Moreover, we utilized gene set variation analysis (GSVA) from the GSEA database C5 BP dataset, which focuses on immune pathways, to evaluate the effect of SMARCAL1 activation on immune pathways. Utilizing data from the TCGA databases, we determined the enrichment score of immune processes. As illustrated in Fig. 6E and Additional file 1: Fig. S5E-8E, SMARCAL1 expression exhibited a negative correlation with HUMORAL_IMMUNE_RESPONSE. Correlations with different immune functions were found by analyzing the link between the enrichment score and SMARCAL1 expression. In conclusion, we investigated the relationship between SMARCAL1 expression and TMB level, followed by calculating their correlation using the Pearson method. The findings showed that SMARCAL1 expression and TMB level were positively correlated in Glioma (R = 0.16, P < 0.001) (Fig. 7A), LUAD (R = 0.16, P < 0.001) (Fig. 7B), and KIRC (R = 0.11, P < 0.05) (Fig. 7D), but not in LIHC (R = -0.036, P = 0.49) (Fig. 7C) and UCEC (R = 0.0012, P = 0.98) (Fig. 7E), where no significant correlation was observed.

### Relationship between immune checkpoint genes and SMARCAL1 expression in pan-cancer

**Fig. 8:**
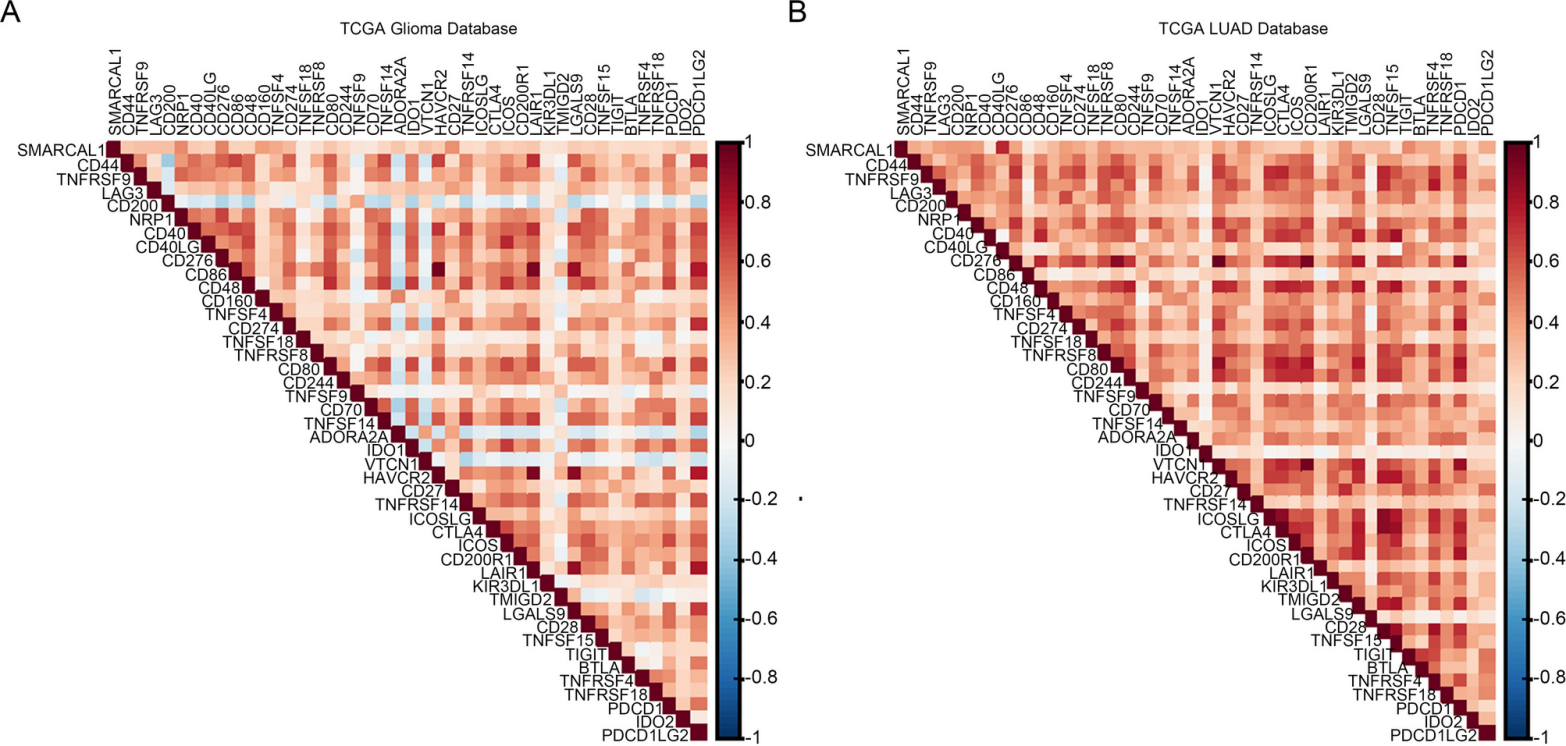
Correlation analysis of SMARACL1 expression with Immune Checkpoints across different cancer types. (**A**) The correlation between SMARACL1 expression and immune checkpoints of Gliomas in the The Cancer Genome Atlas (TCGA) database. (**B**) The correlation between SMARACL1 expression and immune checkpoints of LUAD in the The Cancer Genome Atlas (TCGA) database.

**Fig. 9:**
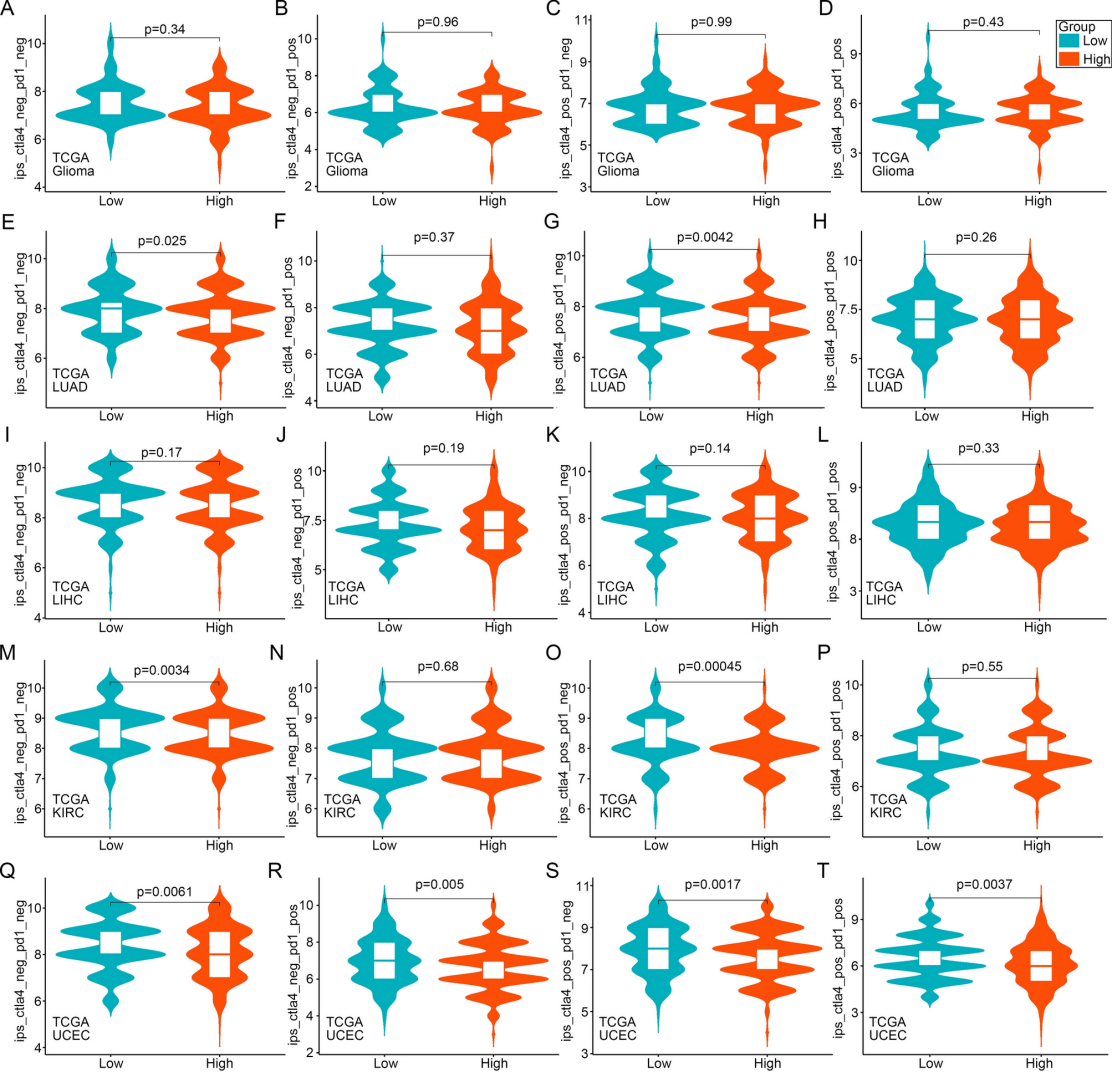
Comparative analysis of ips-ctla4-pd1 in Low and High SMARCAL1 expression groups across various cancer types. (**A**–**D**) Variations in ips-ctla4-pd1 between the Glioma Low-SMARCAL1 and High-SMARCAL1 groups. (**E**–**H**) Variations in ips-ctla4-pd1 between the LUAD Low-SMARCAL1 and High-SMARCAL1 groups. (**I**–**L**) Variations in ips-ctla4-pd1 between the LIHC Low-SMARCAL1 and High-SMARCAL1 groups. (**M**–**P**) Variations in ips-ctla4-pd1 between the KIRC Low-SMARCAL1 and High-SMARCAL1 groups. (**Q**–**T**) Variations in ips-ctla4-pd1 between the UCEC Low-SMARCAL1 and High-SMARCAL1 groups.

In the realm of tumor immunotherapy, immune checkpoints play a pivotal role, significantly influencing the outcomes of immune checkpoint blockade (ICB) treatments^26,27^. Hence, We investigated the connection between immune checkpoint genes and SMARCAL1 expression (Fig. 8, Additional file 1: Fig. S9, and Supplementary Table S4). The results of our investigation showed a strong relationship between the expression of SMARCAL1 and several immune checkpoint genes, including CD276, NRP1, TNFSF4, CD40, CD200, and CD80 in Glioma, LUAD, LIHC, KIRC, and UCEC. Notably, the most relevant immune checkpoint gene is CD276 in Glioma (R = 0.49), LUAD (R = 0.74), LIHC (R = 0.60), KIRC (R = 0.63), and UCEC (R = 0.68). In summary, SMARCAL1 expression exhibited close associations with genes involved in tumor immune modulation. Furthermore, we observed that the scores of ips-ctla4-neg-pd1-neg and ips-ctla4-pos-pd1-neg were notably higher in the low-SMARCAL1 group compared to the high-SMARCAL1 group in LUAD and KIRC (Fig. 9E,G,M,O). Additionally, all four types of ips scores demonstrated significant prominence in UCEC (Fig. 9Q–T). However, there were no significant differences in ips-ctla4-neg-pd1-pos and ips-ctla4-pos-pd1-pos between the two groups in LUAD and KIRC (Fig. 9F,H,N,P), and no discernible differences in the four types of ips scores in Glioma and LIHC (Fig. 9A–D,I–L). These findings suggest that CTLA4 may be more sensitive in the low-SMARCAL1 group in KIRC and LUAD, while both CTLA4 and PD1 may be more sensitive in the low-SMARCAL1 group in UCEC.

### Identification of co-expressed genes and functional analysis of SMARCAL1 expression

**Fig. 10:**
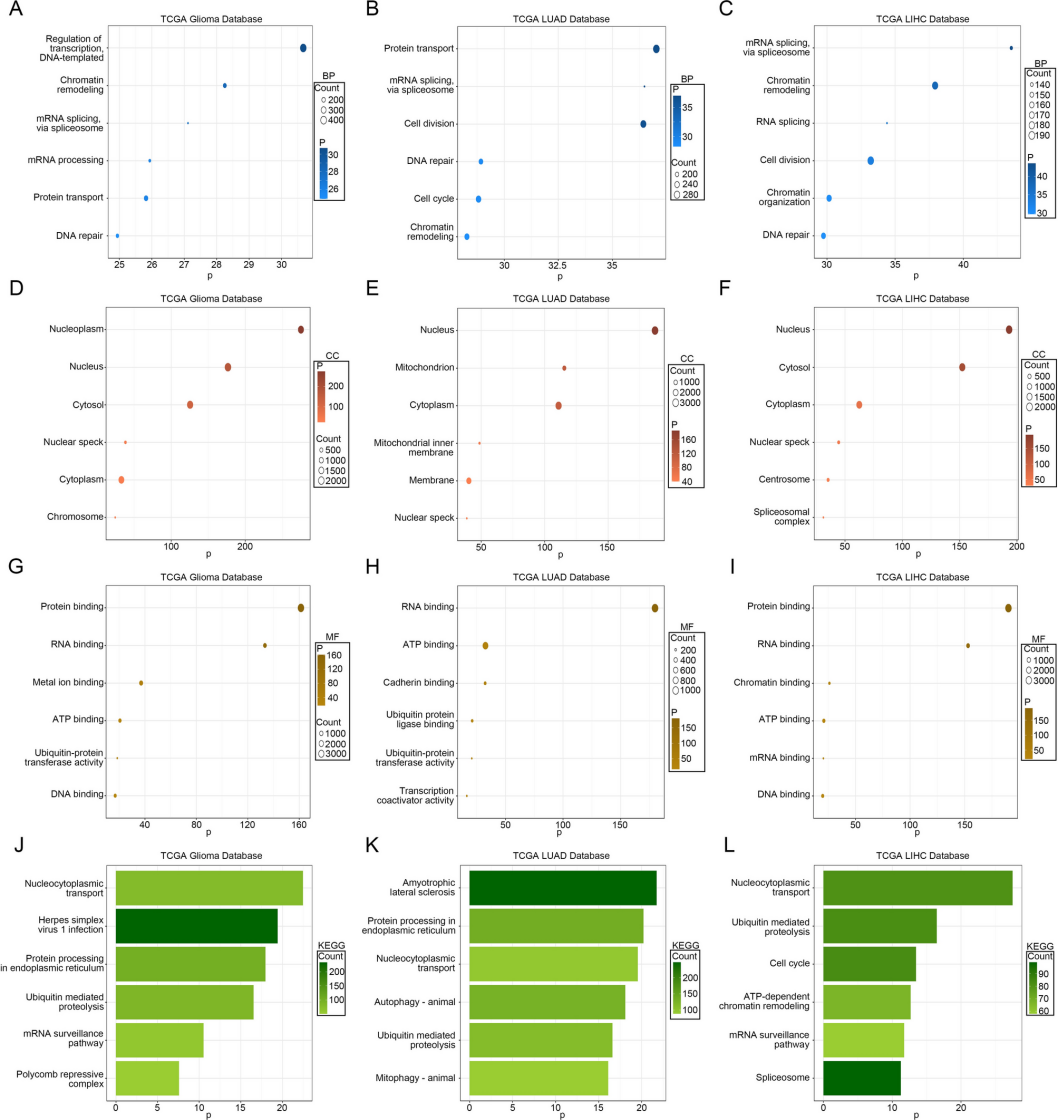
Functional analysis of SMARCAL1 expression across diverse tumor types. (**A**–**C**) Biological processes (BP), (**D**–**F**) cellular components (CC) and (**G**–**I**) molecular functions (MF) are mostly related to SMARCAL1 of Gliomas, LUAD, and LIHC in the The Cancer Genome Atlas (TCGA) database. (**J**–**L**) Kyoto Encyclopedia of Genes and Genomes (KEGG) pathway analysis of SMARCAL11 of Gliomas, LUAD, and LIHC in the The Cancer Genome Atlas (TCGA) database.

To elucidate the biological functions associated with SMARCAL1, we employed Pearson correlation analysis (|R|> 0.5) in the TCGA databases to identify genes most closely correlated with SMARCAL1 expression. Subsequently, GO and KEGG analyses were conducted based on the identified gene sets. In the TCGA database, the biological processes most strongly correlated with SMARCAL1 included chromatin remodeling, mRNA processing, protein transport, DNA repair, and RNA splicing (Fig. 10A–C and Additional file 1: Fig. S10A-B). Additionally, the most associated cellular components of SMARCAL1 were nucleoplasm, cytosol, nucleus, and cytoplasm (Fig. 10D–F and Additional file 1: Fig. S10C-D). Molecular functions primarily involved protein binding, RNA binding, and ATP binding (Fig. 10G–I and Additional file 1: Fig. S10E-F). Moreover, the most closely associated signaling pathway with SMARCAL1 was the nucleocytoplasmic transport pathway (Fig. 10J–L and Additional file 1: Fig. S10G-H). These findings underscore the role of SMARCAL1 expression in preserving genomic stability by engaging in DNA replication and repair processes.

### Pan-cancer sensitivity of SMARCAL1-related drugs

**Fig. 11:**
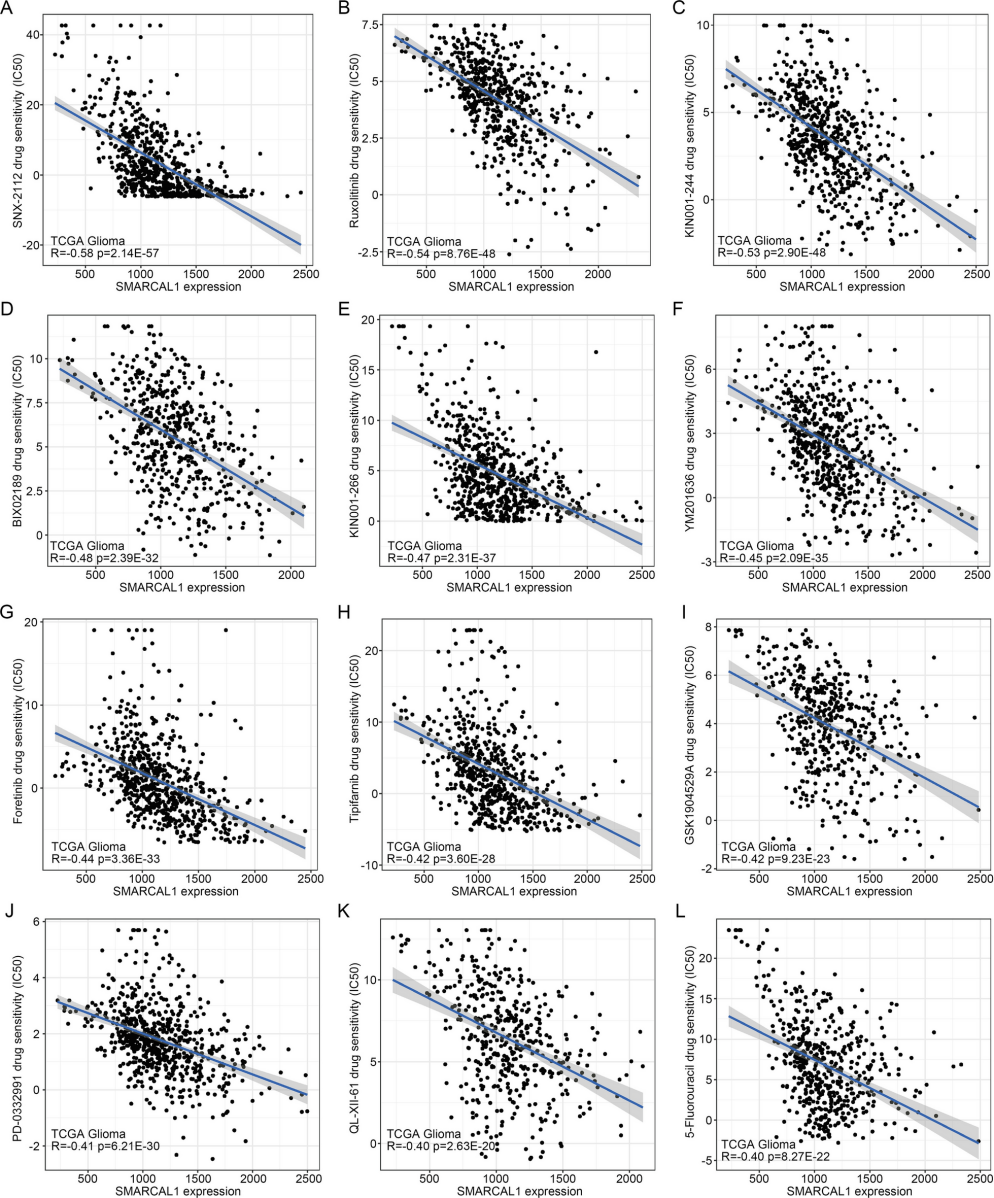
Drug sensitivity analysis of SMARCAL1 expression. The expression of SMARCAL1 was associated with the sensitivity of SNX-2112 (**A**), Ruxolitinib (**B**), KIN001-244 (**C**), BIX02189 (**D**), KIN001-266 (**E**), YM201636 (**F**), Foretinib (**G**), Tipifarnib (**H**), GSK1904529A (**I**), PD-0332991 (**J**), QL-XII-61 (**K**) and 5-Fluorouracil (**L**).

Analysis of the CGP dataset revealed correlations between SMARCAL1 mRNA expression levels and drug sensitivity across various cancers. In Glioma, LUAD, LIHC, KIRC, and UCEC, SMARCAL1 expression exhibited significant correlations with forty-two, thirty-two, twenty-four, fifty, and eighty-three drugs, respectively (|R|> 0.3, p < 0.05, Additional file 1: Supplementary Table S5). Figure 11 illustrates the sensitivity of twelve drugs that are strongly correlated with SMARCAL1 expression in Glioma. Among these correlations, the top three drugs negatively associated with SMARCAL1 expression were SNX-2112 (HSP90 inhibitor, R = -0.58, Fig. 11A), Ruxolitinib (JAK1/2 inhibitor, R = -0.54, Fig. 11B) and KIN001-244 (targeting PDK1, R = -0.53, Fig. 11C) in Glioma; Embelin (XIAP inhibitor, R = -0.70), Roscovitine (CDKs inhibitor, R = -0.62) and BMS-509744 (Itk inhibitor, R = -0.61) in KIRC; MLN4924 (NEDD8-activating Enzyme inhibitor, R = -0.53), Cytarabine (DNA polymerase inhibitor, R = -0.51) and RO-3306 (CDK1 inhibitor, R = -0.51) in LIHC; Mitomycin C (DNA synthesis inhibitor, R = -0.55), Salubrinal (eIF2α dephosphorylation inhibitor, R = -0.54) and HG-5–113-01 (targeting AURKA, R = -0.50) in LUAD; and AKT inhibitor VIII (Akt1, Akt2 and Akt3 inhibitor, R = -0.76), A-443654 (pan-Akt inhibitor, R = -0.75) and Embelin (XIAP inhibitor, R = -0.74) in UCEC. Conversely, drugs positively correlated with SMARCAL1 expression included Navitoclax (Bcl-2 inhibitor, R = 0.40) and Gefitinib (EGFR tyrosine kinase inhibitor, R = 0.34) in Glioma; Phenformin (AMPK activator, R = 0.37) in KIRC; Lapatinib (ErbB-2 and EGFR tyrosine kinase inhibitor, R = 0.34), KU-55933 (ATM inhibitor, R = 0.33), GDC0449 (hedgehog pathway inhibitor, R = 0.32), BEZ235 (PI3K and mTOR kinase inhibitor, R = 0.31) and VX-702 (p38α MAPK inhibitor, R = 0.30) in LIHC; Navitoclax (Bcl-2 inhibitor, R = 0.51) and SB 216,763 (GSK-3 inhibitor, R = 0.42) in LUAD; and Lapatinib (ErbB-2 and EGFR tyrosine kinase inhibitor, R = 0.40) in UCEC. Furthermore, among the drugs associated with three or more of the five tumors were Ruxolitinib (JAK1/2 inhibitor), Tipifarnib (farnesyltransferase inhibitor), GSK1904529A (ATP inhibitor), QL-XII-61 (targeting BMX and BTK), 5-Fluorouracil (thymidylate synthetase inhibitor), BMS-509744 (ATP-competitive Itk inhibitor), Sorafenib (Raf inhibitor), BX-912 (ATP-competitive PDK1 inhibitor), NPK76-II-72–1 (targeting PLK3), Vinorelbine (targeting mitotic), XMD11-85 h (targeting LRRK2 and ERK5), CP466722 (ATM inhibitor), Salubrinal (eIF2α dephosphorylation inhibitor), TL-1–85 (targeting TAK1 and MAP4K2), NG-25 (TAK1 and MAP4K2 inhibitor), PF-562271 (ATP-competitive, FAK and Pyk2 kinase inhibitor) and Pazopanib (VEGFR1, VEGFR2, VEGFR3, PDGFRβ, c-Kit, FGFR1 and c-Fms inhibitor).

### Validation of SMARCAL1 and CD276 in glioma and lung cancer cell lines

**Fig. 12:**
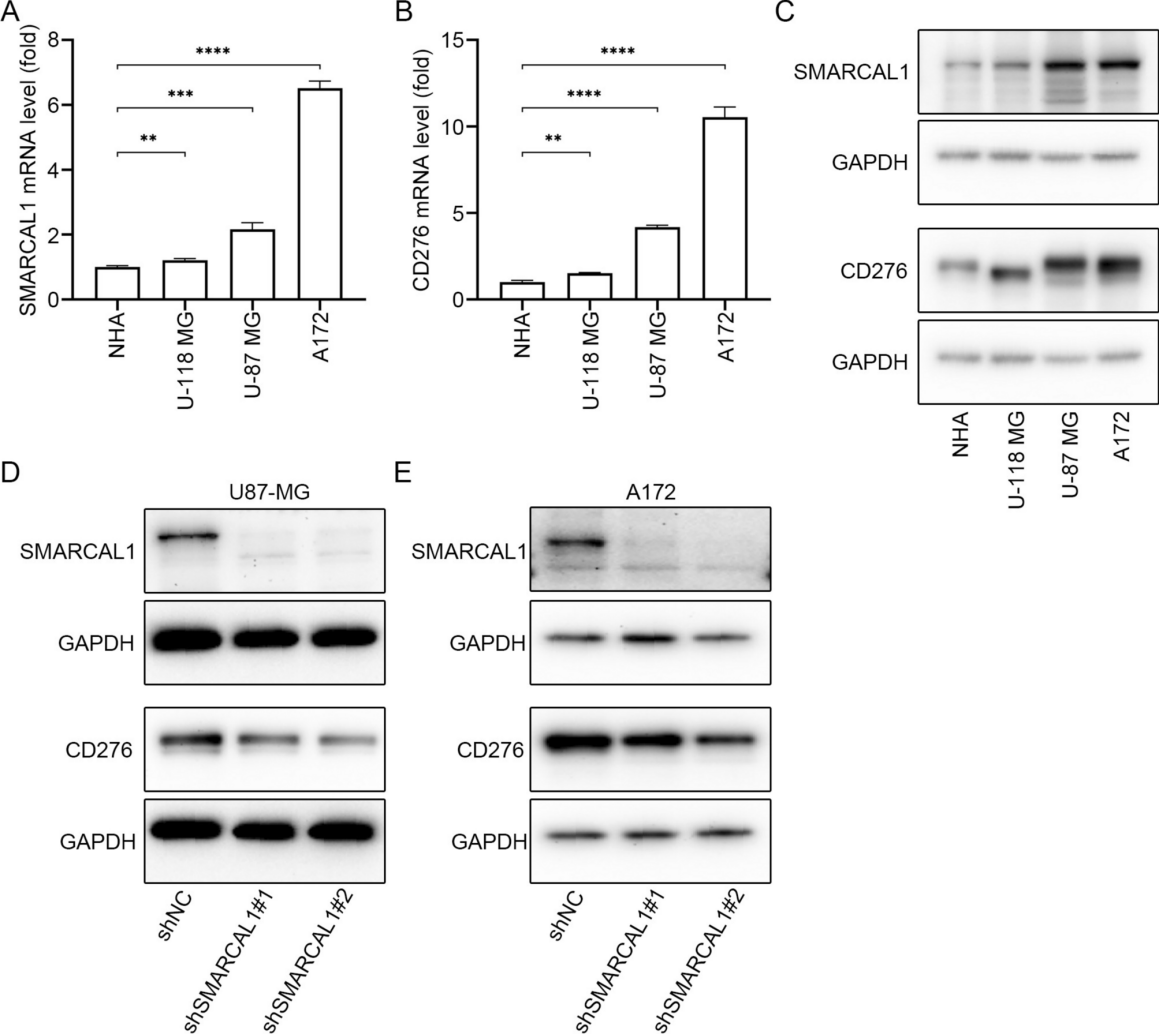
SMARCAL1 and CD276 mRNA and protein expression levels. The levels of SMARCAL1 (**A**) and CD276 (**B**) mRNA expression. The levels of SMARCAL1 and CD276 (**C**) protein expression in Glioma cell lines and astrocyte cell line. The levels of SMARCAL1 and CD276 (**D**,**E**) protein expression after SMARCAL1 knockdown in U87-MG and A172 cell lines, respectively.

We first analyzed the expression levels of SMARCAL1 and CD276 in glioma cell lines (U118-MG, U87-MG, A172) and the normal human astrocyte (NHA) cell line. SMARCAL1 and CD276 mRNA levels were significantly higher in glioma cell lines compared to NHA, as shown in Fig. 12A and B, respectively. At the protein level, both SMARCAL1 and CD276 were also more highly expressed in glioma cell lines than in NHA (Fig. 12C). Next, we assessed SMARCAL1 and CD276 expression in lung cancer cell lines PC9 and HCC827. Interestingly, neither SMARCAL1 nor CD276 was expressed in PC9, while both were detected in HCC827 (Additional file 1: Fig. S11A). To investigate the relationship between SMARCAL1 and CD276, knockdown experiments were performed in glioma cell lines (U118-MG, U87-MG, and A172) and the lung cancer cell line HCC827. In all cases, SMARCAL1 knockdown resulted in a decrease in CD276 protein expression (Fig. 12D,E, and Additional file 1: Fig. S11B-C), suggesting a regulatory role of SMARCAL1 in modulating CD276 expression across different cell lines.

## Discussion

Due to its high incidence and mortality rates, cancer has become a major global public health problem in recent years. Early identification and diagnosis are crucial for improving treatment outcomes^28^. Finding predictive biomarkers for tumors is therefore becoming more and more important for diagnosis, prognosis, and treatment.

In cancer biology, immune regulation is essential because it influences tumor rejection and disease development in different ways depending on circumstances^29,30^. The development of cancer immunotherapies, such as immune checkpoint blockade (ICB), has been facilitated by this understanding^31,32^. The results of immunotherapy for individuals with glioblastoma are still disappointing, despite the fact that different cancer types have shown therapeutic advantages from the treatment^33^. Thus, creating new treatments that take advantage of these pathways and clarifying the molecular underpinnings behind tumor-induced immune suppression are essential for clinical success.

Recent efforts have focused on enhancing anti-tumor immunity by targeting innate immune responses within tumors, with particular attention to the cGAS-STING signaling pathway^34,35^. While genomic instability triggers intrinsic immune responses in cancer, cancer cells often evade these responses by upregulating immune checkpoint modulators, such as PD-L1^36^. According to recent studies, SMARCAL1 limits endogenous DNA damage and prevents signaling that is dependent on cGAS-STING during the growth of cancer cells. Furthermore, it functions in tandem with JUN, a member of the AP-1 family, to preserve the chromatin accessibility of PD-L1 transcriptional regulatory regions, thereby enhancing PD-L1 expression in cancer cells^10^. The findings of this study support growing evidence that suggests SMARCAL1 is a novel potential target for immunotherapy.

The majority of human cancers rely on telomerase for telomere elongation, but approximately 10% utilize a telomerase-independent ALT pathway^37,38^. In order to facilitate homologous recombination-mediated telomere extension, replication stress is necessary for ALT-positive tumors to drive DNA repair mechanisms to telomeres^39^. SMARCAL1 contributes to telomere stability by resolving replication stress and being linked to ALT telomeres^40^.

While SMARCAL1 loss-of-function mutations in telomerase-negative Glioma cells promote selective telomere lengthening and carcinogenesis, its precise function in pan-cancer malignancies is still unknown and has to be clarified^41^. In this study, we used a number of bioinformatics techniques to investigate the possible oncogenic or tumor-suppressive function of SMARCAL1, by assessing the relationship between the expression levels of SMARCAL1 and the following parameters in human pan-cancer cells: overall survival (OS), DNA methylation, tumor mutational burden (TMB), immune infiltration, and classical immune checkpoint genes.

We conducted a pan-cancer analysis of SMARCAL1 across 33 tumor types using the CCLE, TCGA, and GTEx databases. The findings showed that SMARCAL1 was expressed differently in 27 different types of cancers, with 27 tumor types exhibiting higher expression. Furthermore, we investigated the correlation between SMARCAL1 expression and relevant prognostic indicators. Our analysis indicated that higher SMARCAL1 expression posed a significant risk threat in most cancer types. Interestingly, in KIRC tumor tissues, SMARCAL1 mRNA expression levels were higher than in normal tissues, yet significantly correlated with favorable prognosis. We hypothesized that this phenomenon may be attributed to numerous data sources and varying levels of protein expression regulation, which is a primary determinant of cellular function and is influenced by factors beyond mRNA expression^42^.

Additionally, we used mRNA sequencing data from the CGGA and TCGA databases to investigate the expression levels of SMARCAL1 in various pathological subtypes of Glioma. According to our findings, SMARCAL1 is more abundant in malignant Glioma subtypes, which may indicate that it might be used as a prognostic indicator to estimate the risk of developing cancer.

DNA methylation serves as a universal mechanism governing gene expression^43^. Therefore, we looked into the relationship between promoter methylation levels and SMARCAL1 expression. Our findings revealed a significant decrease in promoter methylation levels across most cancer tissues, suggesting a potential oncogenic role of SMARCAL1 in pan-cancer. However, we observed a notable increase in promoter methylation levels in KIRC, consistent with prior reports of elevated SMARCAL1 expression but favorable prognosis in KIRC tumor tissues. Unlike epigenetic alterations, mutations modify the original DNA sequence. Nevertheless, both mutations and epigenetic changes can result in aberrant gene expression^44^. Notably, Endometrial Cancer, characterized by the highest mutation frequency, concomitantly displayed reduced DNA methylation levels. Further investigations are warranted to validate the diagnostic utility of SMARCAL1 and delineate its involvement in the molecular pathogenesis of cancer.

Numerous studies have highlighted a significant correlation between tumorigenesis and the Tumor Microenvironment (TME), an intricate ecosystem surrounding tumors within the body^45^. Understanding the TME facilitates the identification of immune modulators involved in cancer progression and the development of cancer immunotherapies. Tumor-infiltrating immune cells can be manipulated by cancer cells to increase or decrease the efficacy of cancer treatments, which in turn affects the growth of tumors^46,47^. Our study aimed to investigate the connection between the tumor immune microenvironment or immune cells that infiltrate tumors and SMARCAL1 expression. Previous investigations have demonstrated that immune checkpoint genes, such as PD1, PD-L1, and CTLA4, in immunotherapy, are used by malignancies to dampen immune responses^48,49^. Recent studies indicate that SMARCAL1 knockout reduces PD-L1 expression, activates innate immune signaling, and increases the number of CD8 cytotoxic T cells and CD4 T cells^10^. Our results show that UCEC’s low-SMARCAL1 group has larger amounts of CD8 T cells, while other tumor types often have lower levels of CD8 and CD4 T cells.

SMARCAL1 is inversely connected with immune response, despite its functions in DNA integrity preservation and cell cycle regulation. Tumor Mutational Burden (TMB) levels and SMARCAL1 expression in Glioma, LUAD, and KIRC were found to be positively correlated. The production of immunogenic peptides by cancers with elevated TMB levels can modify the effectiveness of immunotherapy^50^. Furthermore, our results indicate a significant correlation between SMARCAL1 expression and most immune checkpoint-related genes. Notably, the most significant correlation is observed with CD276, also known as B7-H3. Despite extensive research, the precise nature of CD276’s impact remains unclear. CD276 exhibits pleiotropic effects on T cells, influencing various aspects of immune regulation^51,52^. It has been implicated in promoting malignant cell behavior and tumor progression through intricate pathways. Further elucidation of CD276’s functional roles is warranted to fully understand its implications in cancer biology and immunotherapy. In our experimental studies, we demonstrated that SMARCAL1 knockdown led to a significant decrease in CD276 expression, further suggesting a regulatory relationship between SMARCAL1 and CD276. This finding highlights the potential importance of SMARCAL1 in immune regulation within the tumor microenvironment, emphasizing its potential role in cancer immunotherapy. Overall, our results show that SMARCAL1 may be important for cancer immunotherapy, and they also emphasize the need for more investigation to clarify its precise modes of action and therapeutic applications. Therefore, SMARCAL1 emerges as a potential target for immunotherapeutic strategies. As SMARCAL1 serves as a prognostic biomarker for cancer, we investigated whether there are any drugs specifically targeting SMARCAL1 in the CGP database. Results revealed Tipifarnib, Sorafenib, and PF-562271 as potential effective inhibitors of SMARCAL1, with most cancers showing sensitivity to them.

To our knowledge, this is the first study to thoroughly examine SMARCAL1’s function in tumor immunology from a pan-cancer standpoint. In addition to offering a preliminary analysis of SMARCAL1’s relationship with immune cell infiltration, immune molecules, and markers of classical immune therapy, our findings shed important light on the potential of SMARCAL1 in cancer immunotherapy. However, limitations persist based on these bioinformatics analyses. Firstly, conflicting results were observed for individual cancers, indicating the need for further investigation into the specific mechanisms underlying these discrepancies. Secondly, sample sizes were small, necessitating testing and validation of SMARCAL1 expression and function in larger sample cohorts. Finally, strong proof from clinical trials is needed for immune cell infiltration in cancer patients, and in vitro or in vivo research is needed to corroborate these findings.

## Conclusion

The multifaceted function of SMARCAL1 in tumor immunology is revealed by our pan-cancer research, indicating that it may be a useful target for cancer immunotherapy. Elevated SMARCAL1 expression across various cancers underscores its significance in tumor progression and immune evasion. The associations identified between SMARCAL1 expression, clinical outcomes, and immune-related factors provide valuable insights into its prognostic and therapeutic implications. To confirm SMARCAL1’s potential as a target for tailored cancer therapies and to clarify the precise processes behind SMARCAL1-mediated immune regulation, more investigation is necessary.

## Supplementary Information

Supplementary Information 1.

Supplementary Information 2.

## Data Availability

The raw data used and/or analyzed during the current study are available from the corresponding author on reasonable request.

## References

[1] Siegel, R. L., Miller, K. D. & Jemal, A. Cancer statistics, 2020. *CA Cancer J. Clin.* **70**(1), 7–30 (2020). 31912902 10.3322/caac.21590

[2] Sung, H. et al. Global Cancer Statistics 2020: GLOBOCAN estimates of incidence and mortality worldwide for 36 cancers in 185 countries. *CA Cancer J. Clin.* **71**(3), 209–249 (2021). 33538338 10.3322/caac.21660

[3] Baig, M. H. et al. Enzyme targeting strategies for prevention and treatment of cancer: Implications for cancer therapy. *Semin. Cancer Biol.* **56**, 1–11 (2019). 29248538 10.1016/j.semcancer.2017.12.003

[4] Balça-Silva, J. et al. Cellular and molecular mechanisms of glioblastoma malignancy: Implications in resistance and therapeutic strategies. *Semin. Cancer Biol.* **58**, 130–141 (2019). 30266571 10.1016/j.semcancer.2018.09.007

[5] Postow, M. A., Callahan, M. K. & Wolchok, J. D. Immune checkpoint blockade in cancer therapy. *J. Clin. Oncol.* **33**(17), 1974–1982 (2015). 25605845 10.1200/JCO.2014.59.4358 PMC4980573

[6] Topalian, S. L. et al. Safety, activity, and immune correlates of anti-PD-1 antibody in cancer. *N. Engl. J. Med.* **366**(26), 2443–2454 (2012). 22658127 10.1056/NEJMoa1200690 PMC3544539

[7] Sharma, P. et al. Primary, adaptive, and acquired resistance to cancer immunotherapy. *Cell* **168**(4), 707–723 (2017). 28187290 10.1016/j.cell.2017.01.017 PMC5391692

[8] Pardoll, D. M. The blockade of immune checkpoints in cancer immunotherapy. *Nat. Rev. Cancer* **12**(4), 252–264 (2012). 22437870 10.1038/nrc3239 PMC4856023

[9] Sharma, P. & Allison, J. P. The future of immune checkpoint therapy. *Science* **348**(6230), 56–61 (2015). 25838373 10.1126/science.aaa8172

[10] Leuzzi, G. et al. SMARCAL1 is a dual regulator of innate immune signaling and PD-L1 expression that promotes tumor immune evasion. *Cell* **187**(4), 861-881.e32 (2024). 38301646 10.1016/j.cell.2024.01.008 PMC10980358

[11] Bétous, R. et al. Substrate-selective repair and restart of replication forks by DNA translocases. *Cell Rep.* **3**(6), 1958–1969 (2013). 23746452 10.1016/j.celrep.2013.05.002 PMC3700663

[12] Ciccia, A. et al. The SIOD disorder protein SMARCAL1 is an RPA-interacting protein involved in replication fork restart. *Genes Dev.* **23**(20), 2415–2425 (2009). 19793862 10.1101/gad.1832309 PMC2764500

[13] Dewar, J. M. & Walter, J. C. Mechanisms of DNA replication termination. *Nat. Rev. Mol. Cell Biol.* **18**(8), 507–516 (2017). 28537574 10.1038/nrm.2017.42 PMC6386472

[14] Nusinow, D. P. et al. Quantitative proteomics of the cancer cell line encyclopedia. *Cell* **180**(2), 387-402.e16 (2020). 31978347 10.1016/j.cell.2019.12.023 PMC7339254

[15] Goldman, M. J. et al. Visualizing and interpreting cancer genomics data via the Xena platform. *Nat. Biotechnol.* **38**(6), 675–678 (2020). 32444850 10.1038/s41587-020-0546-8 PMC7386072

[16] Zhao, Z. et al. Chinese Glioma Genome Atlas (CGGA): A comprehensive resource with functional genomic data from Chinese glioma patients. *Genom. Proteom. Bioinform.* **19**(1), 1–12 (2021). 10.1016/j.gpb.2020.10.005 PMC8498921 33662628

[17] Cerami, E. et al. The cBio cancer genomics portal: An open platform for exploring multidimensional cancer genomics data. *Cancer Discov.* **2**(5), 401–404 (2012). 22588877 10.1158/2159-8290.CD-12-0095 PMC3956037

[18] Chandrashekar, D. S. et al. UALCAN: A portal for facilitating tumor subgroup gene expression and survival analyses. *Neoplasia* **19**(8), 649–658 (2017). 28732212 10.1016/j.neo.2017.05.002 PMC5516091

[19] Chandrashekar, D. S. et al. UALCAN: An update to the integrated cancer data analysis platform. *Neoplasia* **25**, 18–27 (2022). 35078134 10.1016/j.neo.2022.01.001 PMC8788199

[20] Sherman, B. T. et al. DAVID: A web server for functional enrichment analysis and functional annotation of gene lists (2021 update). *Nucleic Acids Res.* **50**(W1), W216-w221 (2022). 35325185 10.1093/nar/gkac194 PMC9252805

[21] da Huang, W., Sherman, B. T. & Lempicki, R. A. Systematic and integrative analysis of large gene lists using DAVID bioinformatics resources. *Nat. Protoc.* **4**(1), 44–57 (2009). 19131956 10.1038/nprot.2008.211

[22] Kanehisa, M. & Goto, S. KEGG: Kyoto encyclopedia of genes and genomes. *Nucleic Acids Res.* **28**(1), 27–30 (2000). 10592173 10.1093/nar/28.1.27 PMC102409

[23] Kanehisa, M. et al. KEGG: Biological systems database as a model of the real world. *Nucleic Acids Res.* **53**(D1), D672-d677 (2025). 39417505 10.1093/nar/gkae909 PMC11701520

[24] Kanehisa, M. Toward understanding the origin and evolution of cellular organisms. *Protein Sci.* **28**(11), 1947–1951 (2019). 31441146 10.1002/pro.3715 PMC6798127

[25] Liu, H. et al. Tumor-infiltrating lymphocytes predict response to chemotherapy in patients with advance non-small cell lung cancer. *Cancer Immunol. Immunother.* **61**(10), 1849–1856 (2012). 22456757 10.1007/s00262-012-1231-7 PMC11029471

[26] Basudan, A. M. The role of immune checkpoint inhibitors in cancer therapy. *Clin. Pract.* **13**(1), 22–40 (2022). 36648843 10.3390/clinpract13010003 PMC9844484

[27] Hu, F. F. et al. Expression profile of immune checkpoint genes and their roles in predicting immunotherapy response. *Brief. Bioinform.*10.1093/bib/bbaa176 (2021). 32814346 10.1093/bib/bbaa176

[28] Bray, F. et al. Global cancer statistics 2022: GLOBOCAN estimates of incidence and mortality worldwide for 36 cancers in 185 countries. *CA Cancer J. Clin.*10.3322/caac.21834 (2024). 38572751 10.3322/caac.21834

[29] Devaud, C. et al. Immune modulation of the tumor microenvironment for enhancing cancer immunotherapy. *Oncoimmunology* **2**(8), e25961 (2013). 24083084 10.4161/onci.25961 PMC3782527

[30] Kim, K. et al. Eradication of metastatic mouse cancers resistant to immune checkpoint blockade by suppression of myeloid-derived cells. *Proc. Natl. Acad. Sci. U. S. A.* **111**(32), 11774–11779 (2014). 25071169 10.1073/pnas.1410626111 PMC4136565

[31] Byun, D. J. et al. Cancer immunotherapy—Immune checkpoint blockade and associated endocrinopathies. *Nat. Rev. Endocrinol.* **13**(4), 195–207 (2017). 28106152 10.1038/nrendo.2016.205 PMC5629093

[32] He, X. & Xu, C. Immune checkpoint signaling and cancer immunotherapy. *Cell Res.* **30**(8), 660–669 (2020). 32467592 10.1038/s41422-020-0343-4 PMC7395714

[33] Yang, M. et al. Immunotherapy for glioblastoma: Current state, challenges, and future perspectives. *Cancers (Basel)* **12**(9), 2334 (2020). 32824974 10.3390/cancers12092334 PMC7565291

[34] Rameshbabu, S. et al. Targeting innate immunity in cancer therapy. *Vaccines (Basel)* **9**(2), 138 (2021). 33572196 10.3390/vaccines9020138 PMC7916062

[35] Zheng, J. et al. Comprehensive elaboration of the cGAS-STING signaling axis in cancer development and immunotherapy. *Mol. Cancer* **19**(1), 133 (2020). 32854711 10.1186/s12943-020-01250-1 PMC7450153

[36] Xu, Y. et al. A tumor-specific super-enhancer drives immune evasion by guiding synchronous expression of PD-L1 and PD-L2. *Cell Rep.* **29**(11), 3435-3447.e4 (2019). 31825827 10.1016/j.celrep.2019.10.093

[37] Shay, J. W. & Wright, W. E. Telomeres and telomerase: Three decades of progress. *Nat. Rev. Genet.* **20**(5), 299–309 (2019). 30760854 10.1038/s41576-019-0099-1

[38] Yadav, T. et al. TERRA and RAD51AP1 promote alternative lengthening of telomeres through an R- to D-loop switch. *Mol. Cell* **82**(21), 3985-4000.e4 (2022). 36265486 10.1016/j.molcel.2022.09.026 PMC9637728

[39] Walsh, K. M. et al. Telomere maintenance and the etiology of adult glioma. *Neuro Oncol.* **17**(11), 1445–1452 (2015). 26014050 10.1093/neuonc/nov082 PMC4648301

[40] Diplas, B. H. et al. The genomic landscape of TERT promoter wildtype-IDH wildtype glioblastoma. *Nat. Commun.* **9**(1), 2087 (2018). 29802247 10.1038/s41467-018-04448-6 PMC5970234

[41] Liu, H. et al. Cancer-associated SMARCAL1 loss-of-function mutations promote alternative lengthening of telomeres and tumorigenesis in telomerase-negative glioblastoma cells. *Neuro Oncol.* **25**(9), 1563–1575 (2023). 36689342 10.1093/neuonc/noad022 PMC10484176

[42] Muniz, L., Nicolas, E. & Trouche, D. RNA polymerase II speed: A key player in controlling and adapting transcriptome composition. *Embo J.* **40**(15), e105740 (2021). 34254686 10.15252/embj.2020105740 PMC8327950

[43] Kim, M. & Costello, J. DNA methylation: An epigenetic mark of cellular memory. *Exp. Mol. Med.* **49**(4), e322 (2017). 28450738 10.1038/emm.2017.10 PMC6130213

[44] Choi, J. D. & Lee, J. S. Interplay between epigenetics and genetics in cancer. *Genomics Inform.* **11**(4), 164–173 (2013). 24465226 10.5808/GI.2013.11.4.164 PMC3897842

[45] Janji, B., Berchem, G. & Chouaib, S. Targeting autophagy in the tumor microenvironment: New challenges and opportunities for regulating tumor immunity. *Front. Immunol.* **9**, 887 (2018). 29922284 10.3389/fimmu.2018.00887 PMC5996896

[46] Ferrone, C. & Dranoff, G. Dual roles for immunity in gastrointestinal cancers. *J. Clin. Oncol.* **28**(26), 4045–4051 (2010). 20644090 10.1200/JCO.2010.27.9992 PMC4872327

[47] Wang, J. et al. Crosstalk between cancer and immune cells: Role of tumor-associated macrophages in the tumor microenvironment. *Cancer Med.* **8**(10), 4709–4721 (2019). 31222971 10.1002/cam4.2327 PMC6712467

[48] Zhang, Q. et al. Inhibition of ATM increases interferon signaling and sensitizes pancreatic cancer to immune checkpoint blockade therapy. *Cancer Res.* **79**(15), 3940–3951 (2019). 31101760 10.1158/0008-5472.CAN-19-0761 PMC6684166

[49] Sheng, W. et al. LSD1 ablation stimulates anti-tumor immunity and enables checkpoint blockade. *Cell* **174**(3), 549-563.e19 (2018). 29937226 10.1016/j.cell.2018.05.052 PMC6063761

[50] Fumet, J. D. et al. Tumour mutational burden as a biomarker for immunotherapy: Current data and emerging concepts. *Eur. J. Cancer* **131**, 40–50 (2020). 32278982 10.1016/j.ejca.2020.02.038 PMC9473693

[51] Zhao, B. et al. Immune checkpoint of B7–H3 in cancer: From immunology to clinical immunotherapy. *J. Hematol. Oncol.* **15**(1), 153 (2022). 36284349 10.1186/s13045-022-01364-7 PMC9597993

[52] Cheng, M. et al. CD276-dependent efferocytosis by tumor-associated macrophages promotes immune evasion in bladder cancer. *Nat. Commun.* **15**(1), 2818 (2024). 38561369 10.1038/s41467-024-46735-5 PMC10985117

