# Supplementary material for: Pan-cancer analysis reveals SMARCAL1 expression is associated with immune cell infiltration and poor prognosis in various cancers
Source: Sci Rep. 2025 Feb 24;15:6591. doi: 10.1038/s41598-025-88955-9 (PMC11850860; doi:10.1038/s41598-025-88955-9)
Supplement: Supplementary file 1 — Supplementary Information 1. [file 41598_2025_88955_MOESM1_ESM.pdf]

Additional file 1 Supplementary Fig. S1

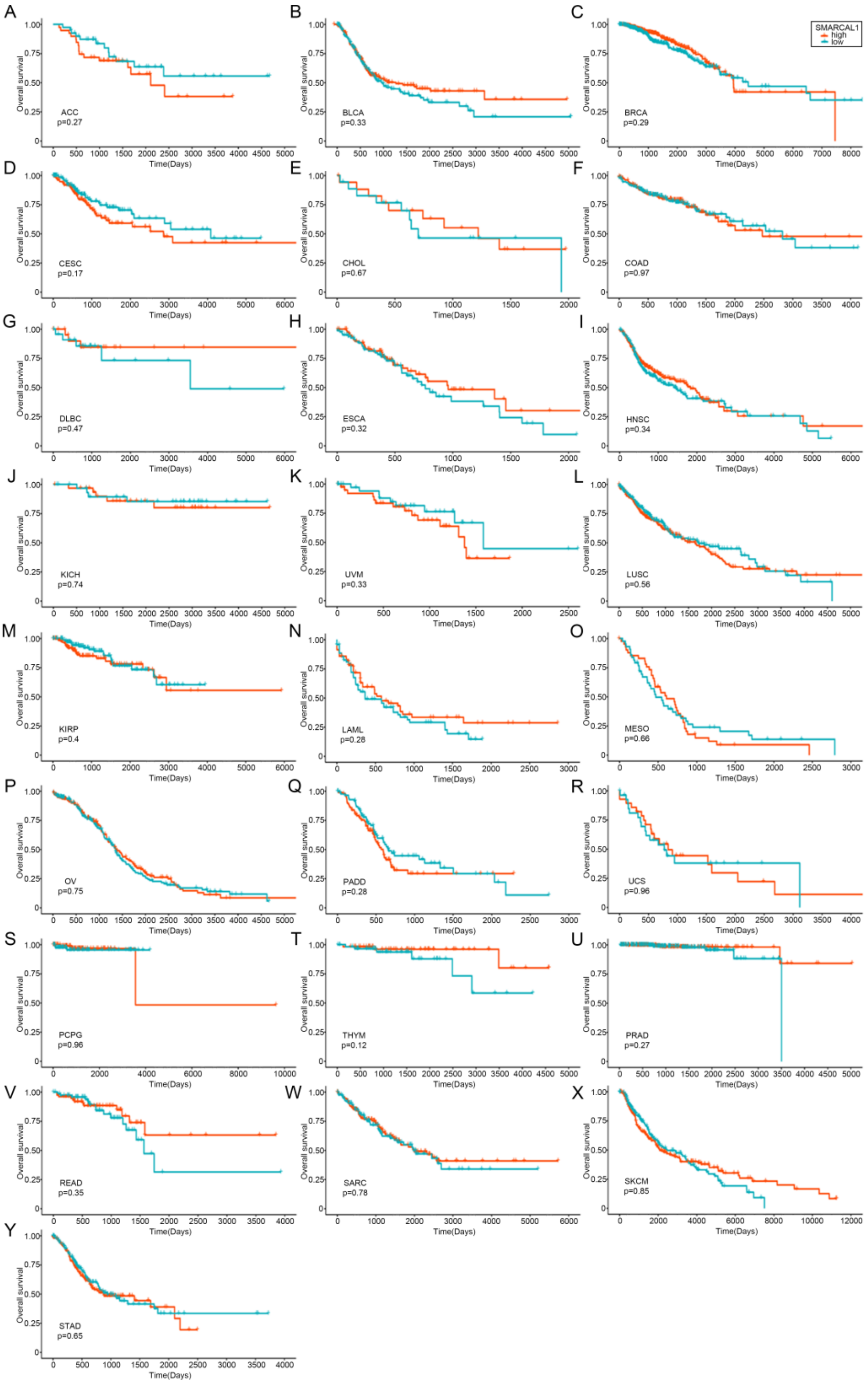

Fig. S1. The prognostic role of SMARCAL1 in human pan-cancer. (A) OS curves using the Kaplan-Meier approach that contrast high and low expression of SMARCAL1 in ACC, BLCA, BRCA, CESC, CHOL, COAD, DLBC, ESCA, HNSC, KICH, UVM, LUSC, KIRP, LAML, MESO, OV, PADD, UCS, PCPG, THYM,

PRAD, READ, SARC, SKCM, and STAD.

Additional file 1 Supplementary Fig. S2

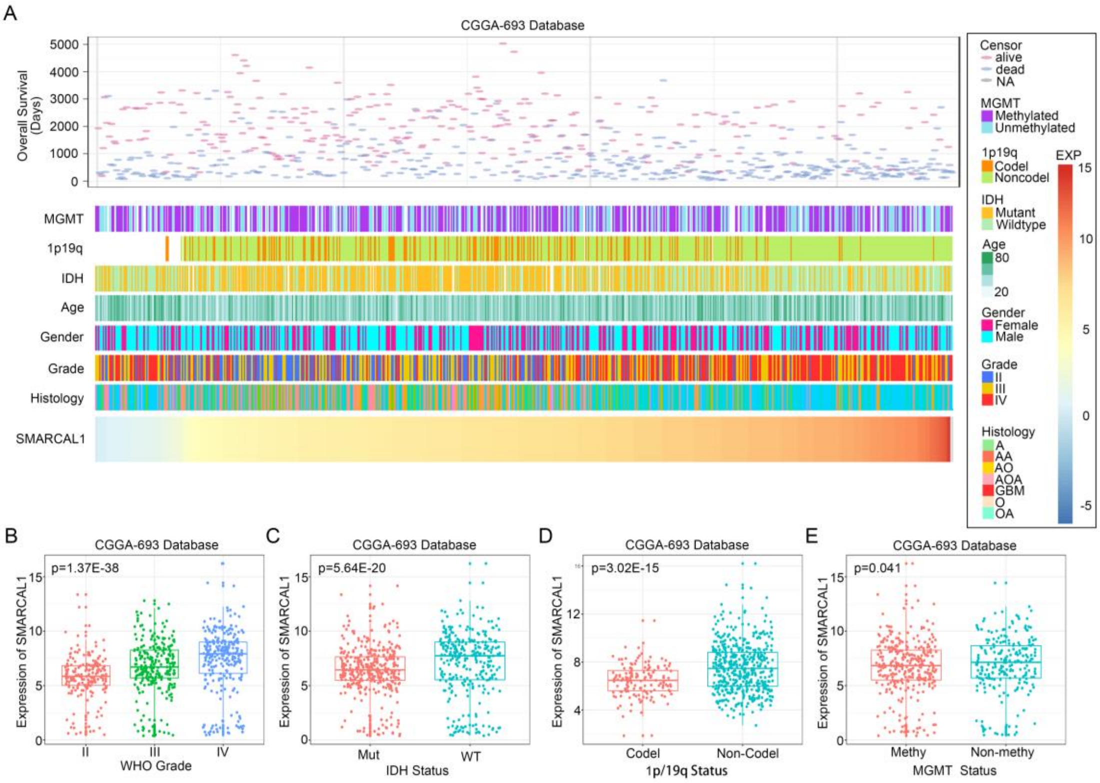

Fig. S2. Relationship between the clinicopathological features of Gliomas and SMARCAL1. (A) The distribution of clinicopathological characteristics associated with SMARCAL1 in Gliomas within the Chinese Glioma Genome Atlas (CGGA) 693 database. (B) The CGGA 693 dataset showed a substantial increase in SMARCAL1 in higher-grade Gliomas. One-way ANOVA was used to determine the significance of the difference. (C) The CGGA 693 dataset showed a substantial increase in SMARCAL1 in Gliomas without an isocitrate dehydrogenase (IDH) mutation. An unpaired t-test was used to determine the difference's significance. (D) In the CGGA 693 dataset, SMARCAL1 was markedly elevated in Gliomas without 1p/19q codeletion. An unpaired t-test was used to determine the difference's significance. (E) Increased levels of SMARCAL1 were observed in unmethylated Gliomas with the O6-methylguanine-DNA methyltransferase (MGMT) promoter in the CGGA 693 dataset. An unpaired t-test was used to determine the difference's significance.

Additional file 1 Supplementary Fig. S3

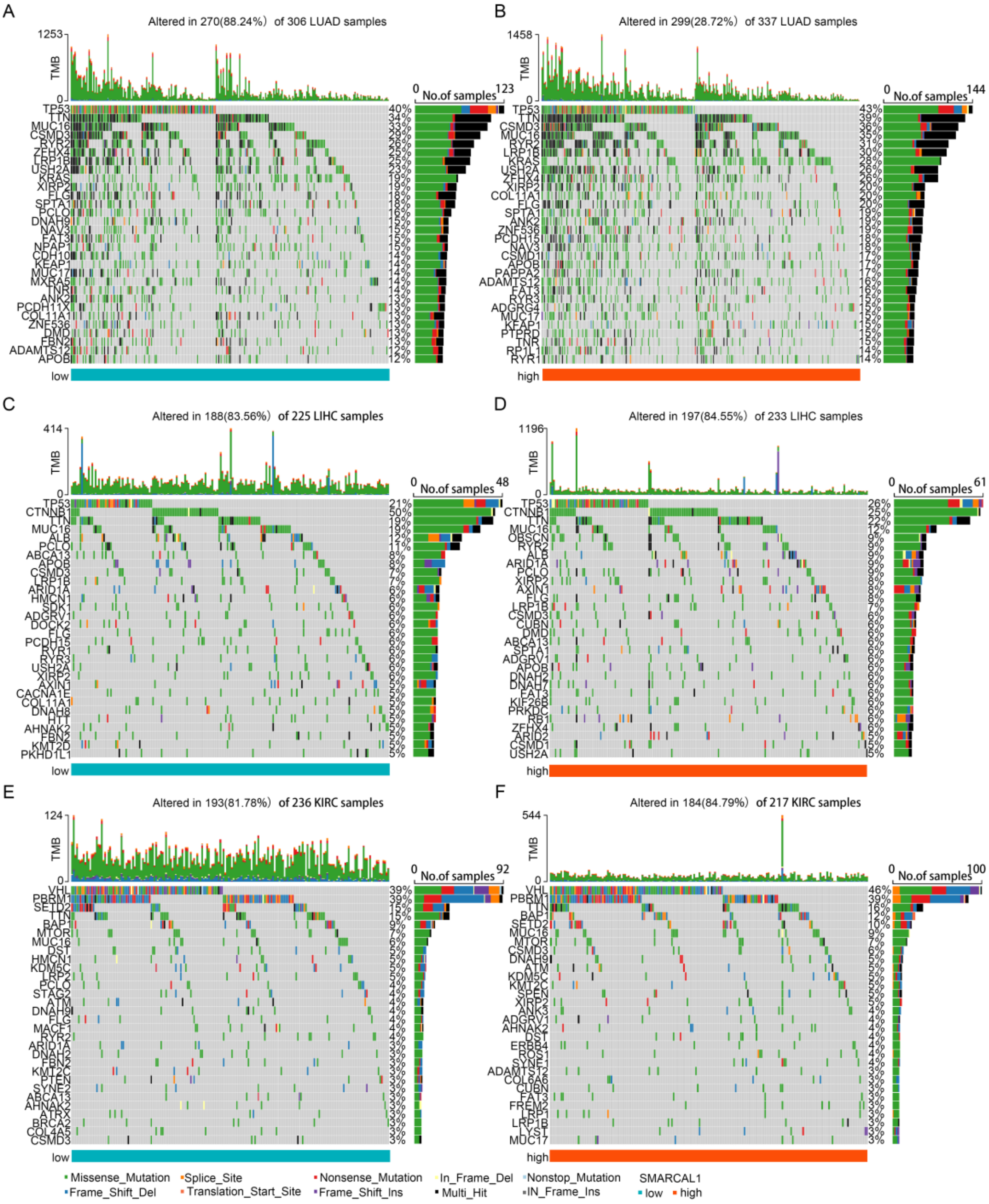

Fig. S3. Genetic alteration analysis of SMARCAL1. (A-B) The top 30 genes in The Cancer Genome Atlas (TCGA) database that have the highest frequency of mutations in the low SMARCAL1 expression group and the high SMARCAL1 expression group of LUAD. (C-D) The top 30 genes in The Cancer Genome Atlas (TCGA) database that have the highest frequency of mutations in the low SMARCAL1 expression group and the high SMARCAL1 expression group of LIHC. (E-F) The top 30 genes in The Cancer Genome

Atlas (TCGA) database that have the highest frequency of mutations in the low SMARCAL1 expression group and the high SMARCAL1 expression group of KIRC.

Additional file 1 Supplementary Fig. S4

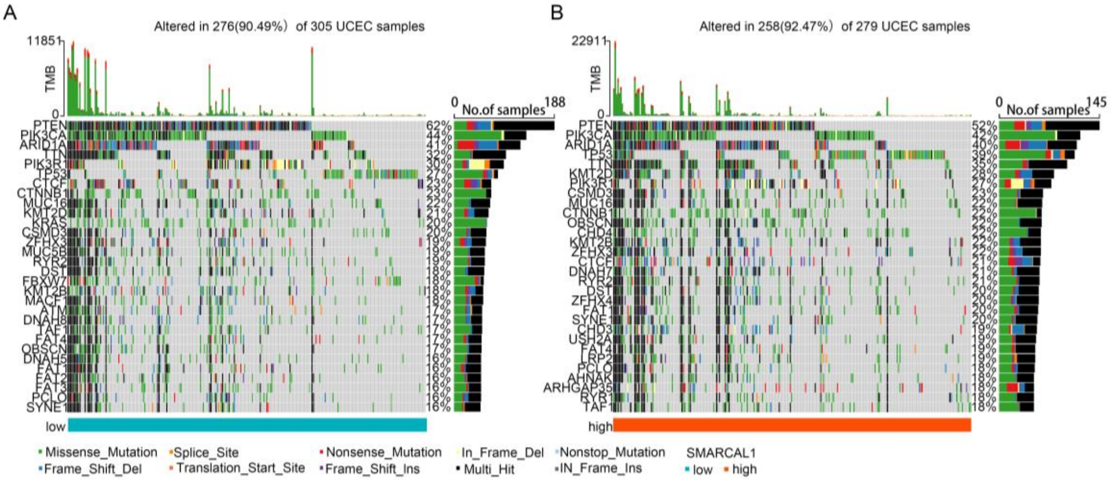

Fig. S4. Genetic alteration analysis of SMARCAL1. (A-B) The top 30 genes in The Cancer Genome Atlas (TCGA) database that have the highest frequency of mutations in the low SMARCAL1 expression group and the high SMARCAL1 expression group of UCEC.

Additional file 1 Supplementary Fig. S5

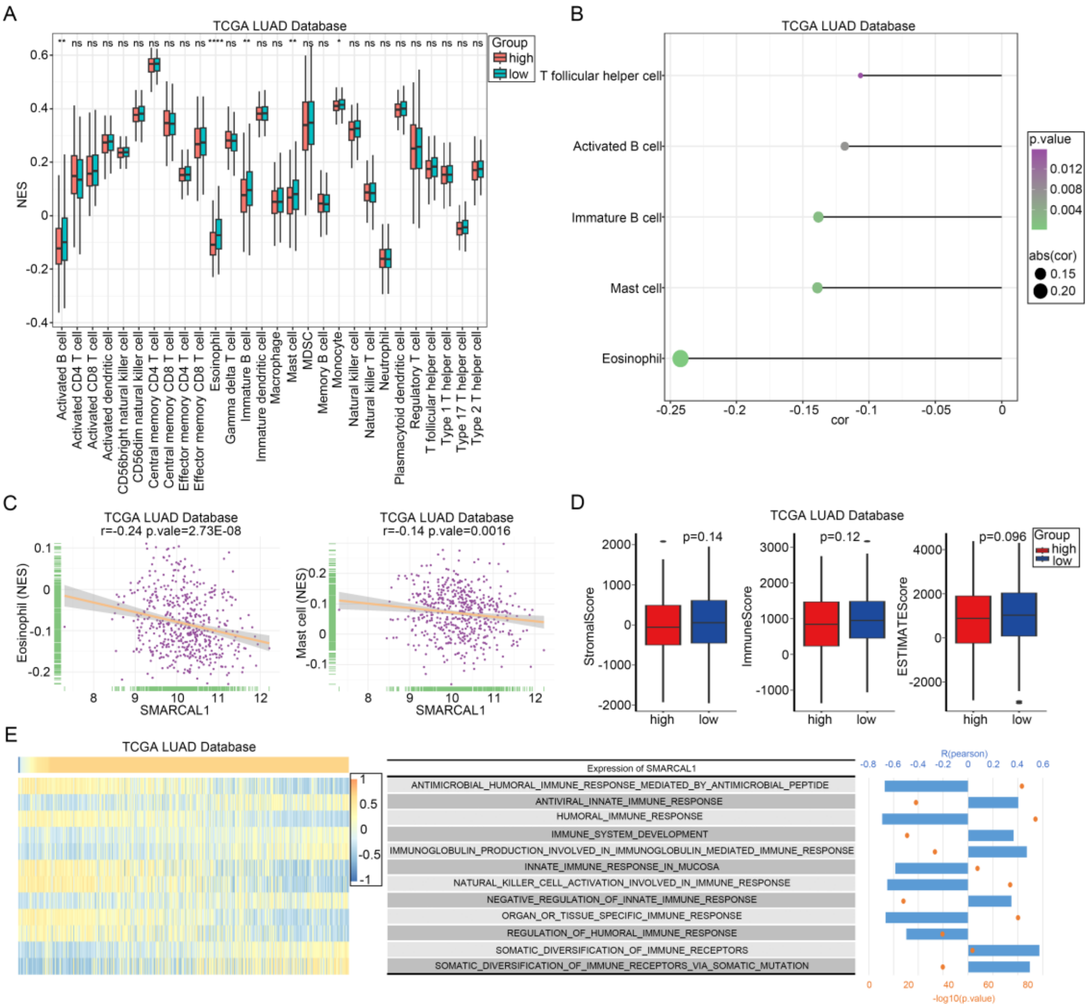

Fig. S5. Tumor infiltration analysis of SMARCAL1. (A) Relationship between the expression of SMARCAL1 and 28 immune cell types that infiltrate tumors in LUAD found in The Cancer Genome Atlas (TCGA) database. (B) T follicular helper cell, Activated B cell, Immature B cell, Mast cell, and Eosinophil were negatively correlated. (C) Eosinophil and Mast cell were negatively linked with SMARCAL1 expression. (D) StromalScore, ImmuneScore, and ESTIMATEScore of SMARCAL1 high and low expression groups of Gliomas in The Cancer Genome Atlas (TCGA) database. (E) Each LUAD patient's SMARCAL1 expression and immunological function enrichment scores were displayed in a heatmap within The Cancer Genome Atlas (TCGA) database. The samples were grouped according to

SMARCAL1 expression in ascending order. The correlation analysis's R- and P-values were displayed in the column and line graphs on the right. ns, $p \geq 0.05$; *$p < 0.05$; **$p < 0.01$; ***$p < 0.001$.

Additional file 1 Supplementary Fig. S6

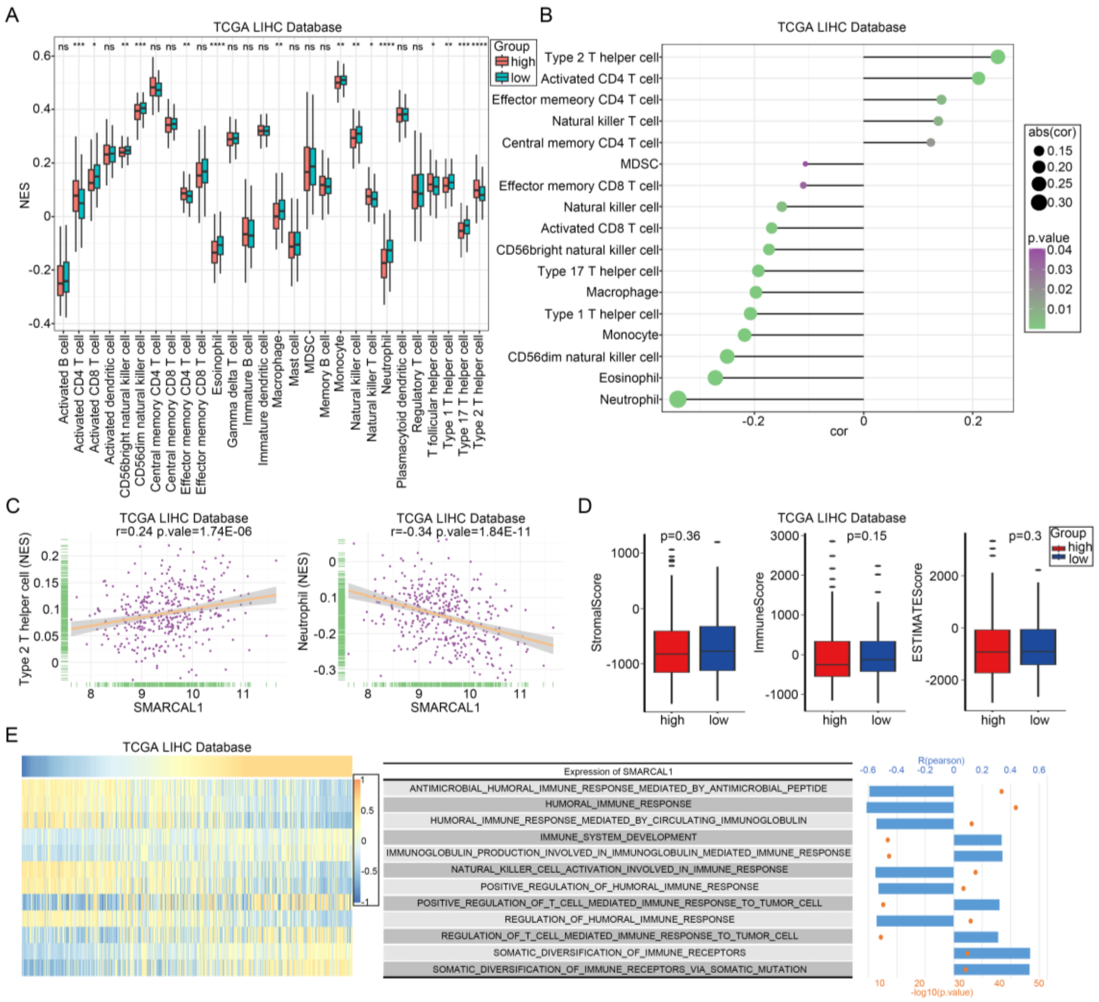

Fig. S6. Tumor infiltration analysis of SMARCAL1. (A) Relationship between the expression of SMARCAL1 and 28 immune cell types that infiltrate tumors in LIHC found in The Cancer Genome Atlas (TCGA) database. (B) Type 2 T helper cell, Activated CD4 T cell, Effector memory CD4 T cell, Nature killer T cell, and Central memory CD4 T cell were positively connected with SMARCAL1 expression, while MDSC, Effector memory CD8 T cell, Natural killer cell, Activated CD8 T cell, CD56bright natural killer cell, Type 17 T helper cell, Macrophage, Type 1 T helper cell, Monocyte, CD56dim natural killer cell, Eosinophil, and Neutrophil were negatively correlated. (C) Type 2 T helper cell was positively linked with SMARCAL1 expression, while Neutrophil cell was negatively correlated. (D) StromalScore, ImmuneScore, and ESTIMATEScore of SMARCAL1 high and low expression groups of LIHC in The

Cancer Genome Atlas (TCGA) database. (E) Each LIHC patient's SMARCAL1 expression and immunological function enrichment scores were displayed in a heatmap within The Cancer Genome Atlas (TCGA) database. The samples were grouped according to SMARCAL1 expression in ascending order. The correlation analysis's R- and P-values were displayed in the column and line graphs on the right. ns, $p \geq 0.05$; *$p < 0.05$; **$p < 0.01$; ***$p < 0.001$.

Additional file 1 Supplementary Fig. S7

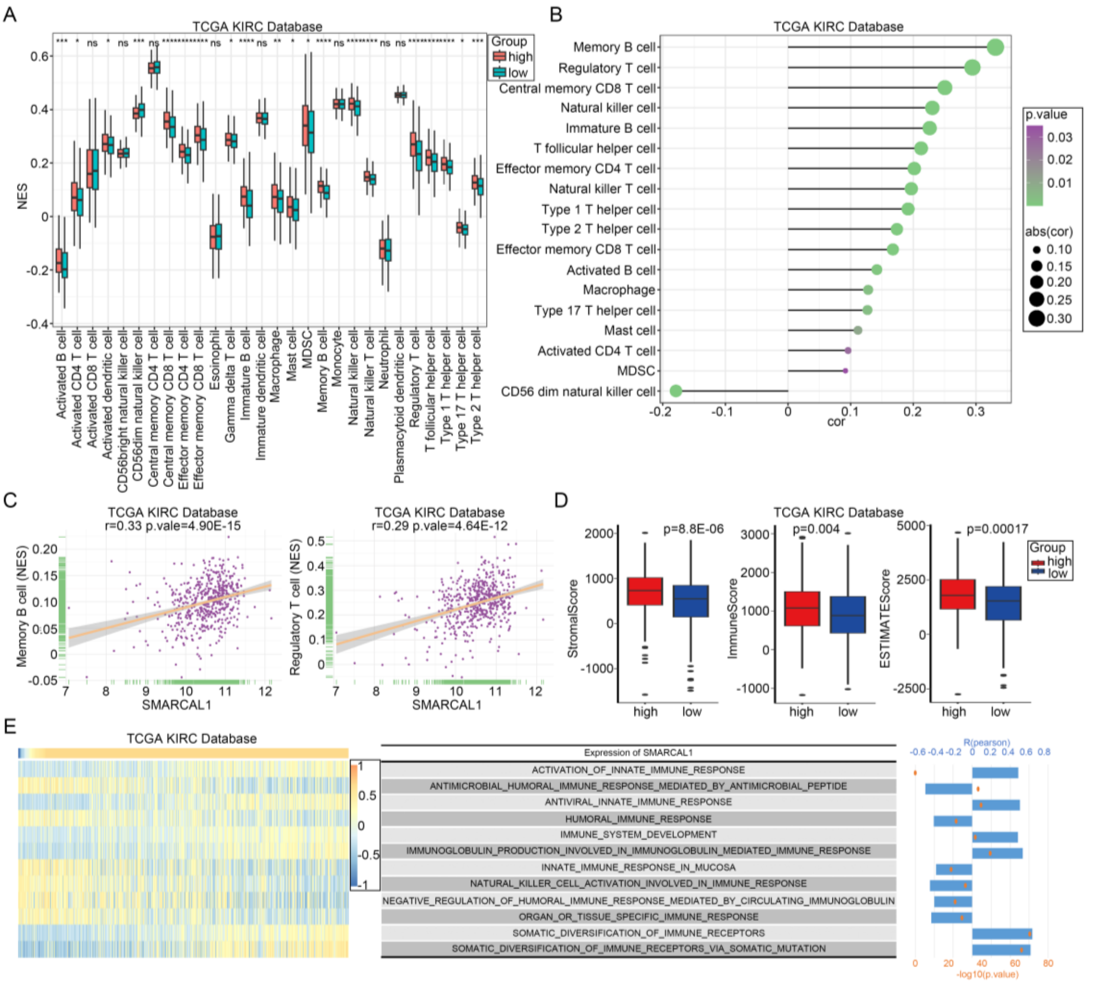

Fig. S7. Tumor infiltration analysis of SMARCAL1. (A) Relationship between the expression of SMARCAL1 and 28 immune cell types that infiltrate tumors in KIRC found in The Cancer Genome Atlas (TCGA) database. (B) Memory B cell, Regulatory T cell, Central memory CD8 T cell, Natural killer cell, Immature B cell, T follicular helper cell, Effector memory CD4 T cell, Natural killer cell, Type 1 T helper cell, Type 2 T helper cell, Effector memory CD8 T cell, Activated B cell, Macrophage, Type 17 T helper cell, Mast cell, Activated CD4 T cell, and MDSC were positively connected with SMARCAL1 expression, while CD56 dim natural killer cell was negatively correlated. (C) Memory B cell and Regulatory T cell were positively linked with SMARCAL1 expression. (D) StromalScore, ImmuneScore, and ESTIMATEScore of SMARCAL1 high and low expression groups of KIRC in The Cancer Genome Atlas (TCGA) database. (E) Each KIRC patient's SMARCAL1 expression and immunological function enrichment scores were

displayed in a heatmap within The Cancer Genome Atlas (TCGA) database. The samples were grouped according to SMARCAL1 expression in ascending order. The correlation analysis's R- and P-values were displayed in the column and line graphs on the right. ns, $p \geq 0.05$; *$p < 0.05$; **$p < 0.01$; ***$p < 0.001$.

Additional file 1 Supplementary Fig. S8

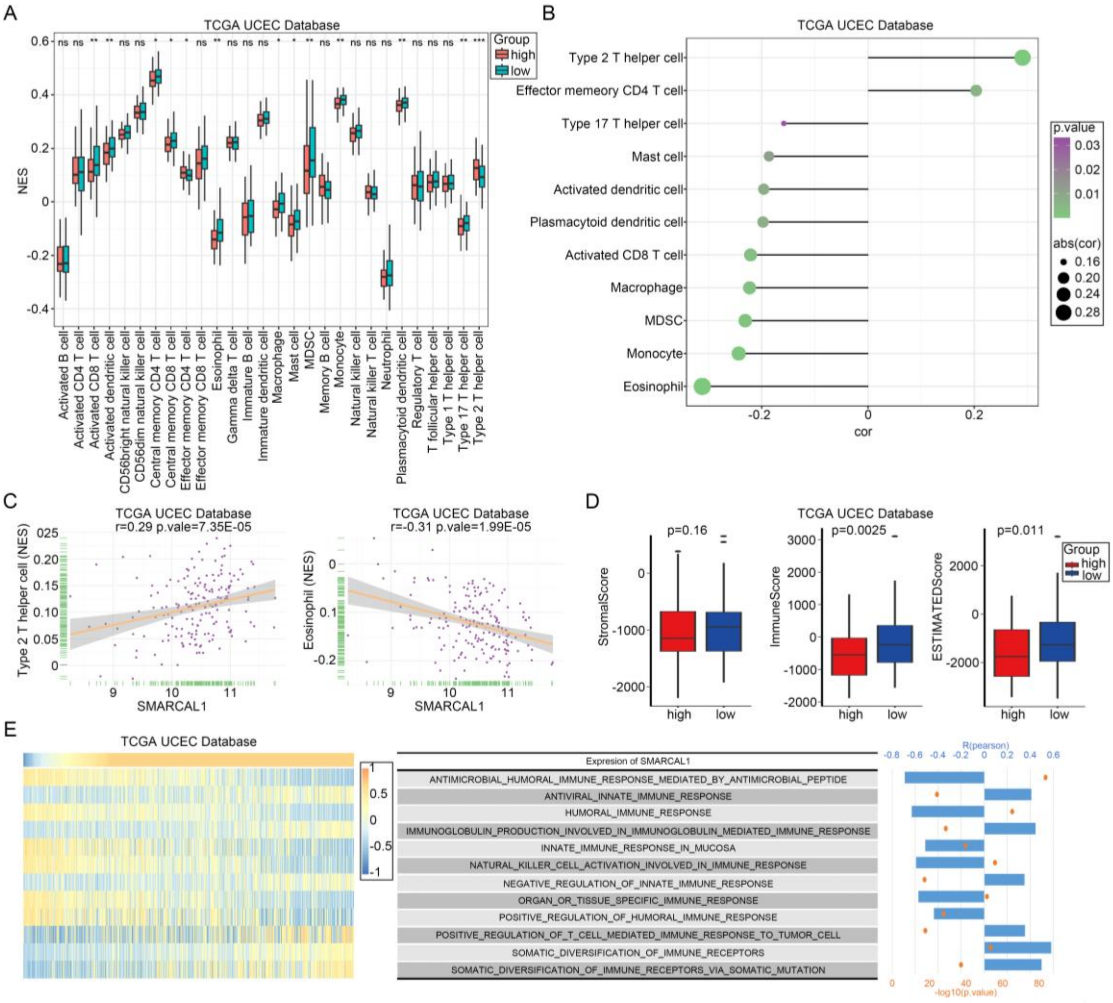

Fig. S8. Tumor infiltration analysis of SMARCAL1. (A) Relationship between the expression of SMARCAL1 and 28 immune cell types that infiltrate tumors in UCEC found in The Cancer Genome Atlas (TCGA) database. (B) Type 2 T helper cell and Effector memory CD4 T cell were positively connected with SMARCAL1 expression, while Type 17 T helper cell, Mast cell, Activated dendritic cell, Plasmacytoid dendritic cell, Activated CD8 T cell, Macrophage, MDSC, Monocyte, and Eosinophil were negatively correlated. (C) Type 2 T helper cell was positively linked with SMARCAL1 expression, while Eosinophil was negatively correlated. (D) StromalScore, ImmuneScore, and ESTIMATEScore of SMARCAL1 high and low expression groups of UCEC in The Cancer Genome Atlas (TCGA) database. (E) Each UCEC patient's SMARCAL1 expression and immunological function enrichment scores were

displayed in a heatmap within The Cancer Genome Atlas (TCGA) database. The samples were grouped according to SMARCAL1 expression in ascending order. The correlation analysis's R- and P-values were displayed in the column and line graphs on the right. ns, $p \geq 0.05$; $^{*}p < 0.05$; $^{**}p < 0.01$; $^{***}p < 0.001$.

Additional file 1 Supplementary Fig. S9

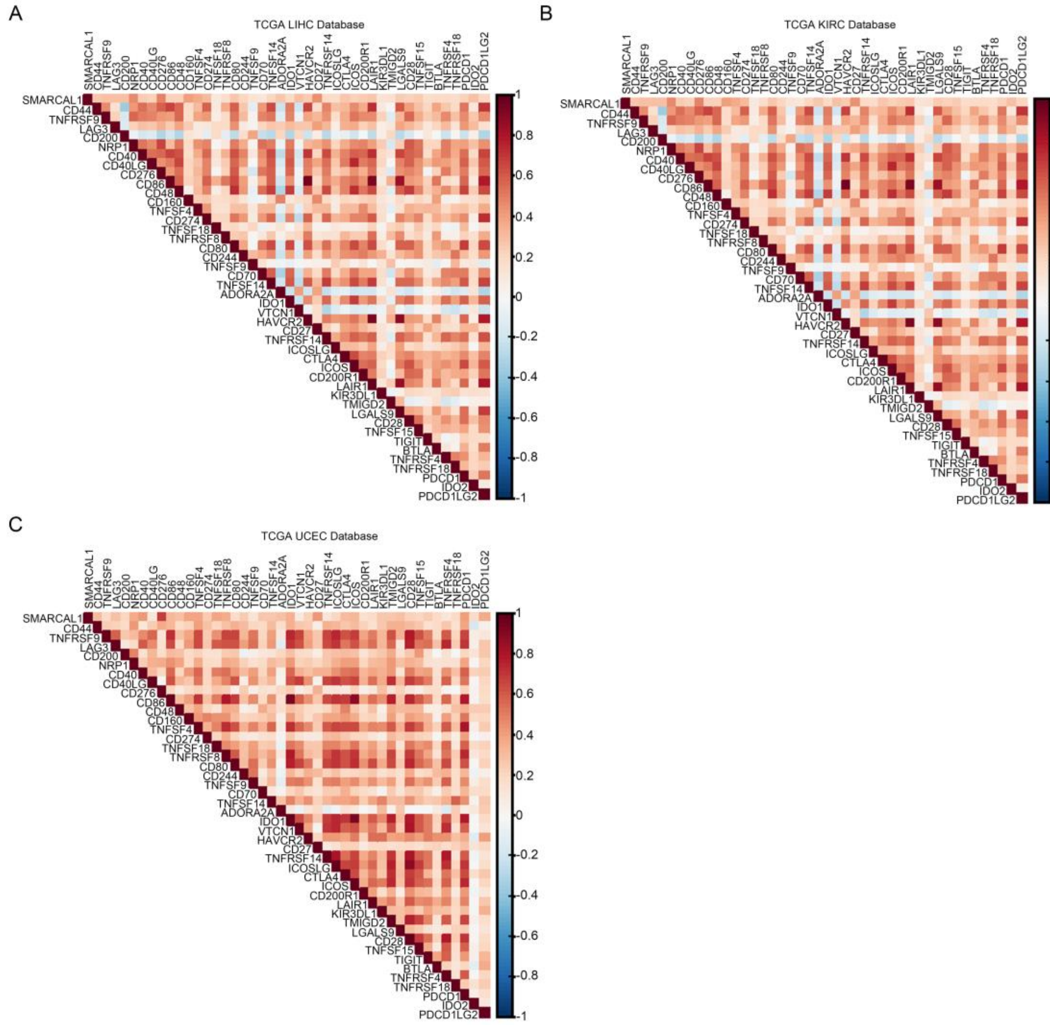

Fig. S9. Correlation analysis of SMARACL1 expression with Immune Checkpoints across different cancer types. (A) The correlation between SMARACL1 expression and immune checkpoints of LIHC in The Cancer Genome Atlas (TCGA) database. (B) The correlation between SMARACL1 expression and immune checkpoints of KIRC in The Cancer Genome Atlas (TCGA) database. (C) The correlation between SMARACL1 expression and immune checkpoints of UCEC in The Cancer Genome Atlas (TCGA) database.

Additional file 1 Supplementary Fig. S10

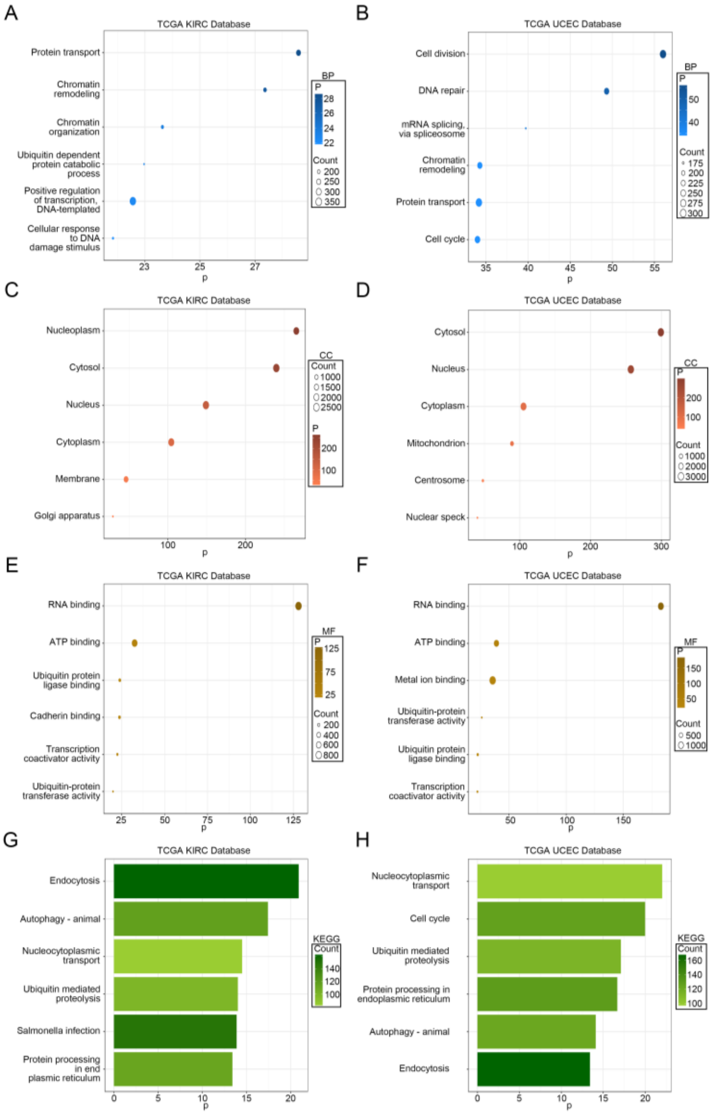

Fig. S10. Functional analysis of SMARCAL1 expression across diverse tumor types. (A-B) Biological processes (BP), (C-D) cellular components (CC), and (E-F) molecular functions (MF) are mostly related to SMARCAL1 of KIRC and UCEC in The Cancer Genome Atlas (TCGA) database. (G-H) Kyoto Encyclopedia of Genes and Genomes (KEGG) pathway analysis of SMARCAL11 of KIRC and UCEC in The Cancer Genome Atlas (TCGA) database.

Additional file 1 Supplementary Fig. S11

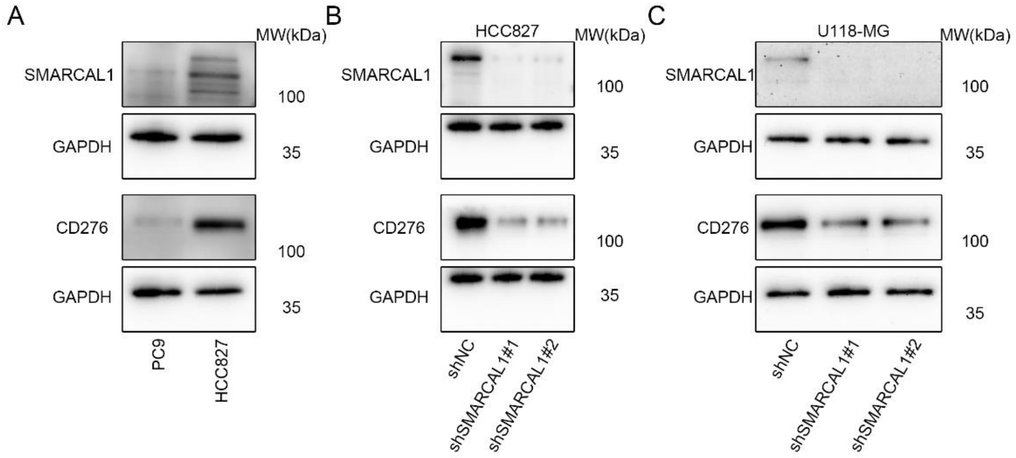

Fig. S11. SMARCAL1 and CD276 protein expression levels. The levels of SMARCAL1 and CD276 (A) protein expression in Lung Cancer cell lines. The levels of SMARCAL1 and CD276 (B-C) protein expression after SMARCAL1 knockdown in HCC827 and U118-MG cell lines, respectively.

Additional file 1 Supplementary Table S1

The correspondence between Cancer species and Abbreviations in TCGA		
ID	Cancer species	Abbreviations
1	Adrenocortical Cancer	ACC
2	Bladder Cancer	BLCA
3	Breast Cancer	BRCA
4	Cervical Cancer	CESC
5	Bile Duct Cancer	CHOL
6	Colon Cancer	COAD
7	Large B-cell Lymphoma	DLBC
8	Esophageal Cancer	ESCA
9	Glioblastoma	GBM
10	Head and Neck Cancer	HNSC
11	Kidney Chromophobe	KICH
12	Kidney Clear Cell Carcinoma	KIRC
13	Kidney Papillary Cell Carcinoma	KIRP
14	Acute Myeloid Leukemia	LAML
15	Lower Grade Glioma	LGG
16	Liver Cancer	LIHC
17	Lung Adenocarcinoma	LUAD
18	Lung Squamous Cell Carcinoma	LUSC
19	Mesothelioma	MESO
20	Ovarian Cancer	OV
21	Pancreatic Cancer	PAAD
22	Pheochromocytoma & Paraganglioma	PCPG
23	Prostate Cancer	PRAD
24	Rectal Cancer	READ
25	Sarcoma	SARC
26	Melanoma	SKCM
27	Stomach Cancer	STAD
28	Testicular Cancer	TGCT
29	Thyroid Cancer	THCA
30	Thymoma (THYM)	THYM
31	Endometrioid Cancer	UCEC
32	Uterine Carcinosarcoma	UCS
33	Ocular melanomas	UVM

The correspondence between Primary Disease and Lineage in CCLE		
ID	Primary Disease	Lineage
1	Squamous Cell Carcinoma of the Vulva/Vagina	Vulva/Vagina-1
2	Hepatocellular Carcinoma plus Intrahepatic Cholangiocarcinoma	Liver-1
3	Mixed Cervical Carcinoma	Cervix-1
4	Cervical Squamous Cell Carcinoma	Cervix-2
5	Ovarian Cancer, Other	Ovary/Fallopian Tube-1
6	Bladder Squamous Cell Carcinoma	Bladder/Urinary Tract-1
7	Retinoblastoma	Eye-1
8	Cervical Adenocarcinoma	Cervix-3
9	Breast Ductal Carcinoma In Situ	Breast-1
10	Head and Neck Squamous Cell Carcinoma	Head and Neck
11	Gestational Trophoblastic Disease	Uterus-1
12	Esophageal Squamous Cell Carcinoma	Esophagus/Stomach-1
13	Intracholecystic Papillary Neoplasm	Biliary Tract-1
14	Colorectal Adenocarcinoma	Bowel-1
15	Poorly Differentiated Thyroid Cancer	Thyroid-1
16	Urethral Cancer	Bladder/Urinary Tract-2
17	Intraductal Papillary Neoplasm of the Bile Duct	Biliary Tract-2
18	Invasive Breast Carcinoma	Breast-2
19	Ampullary Carcinoma	Ampulla of Vater
20	Breast Neoplasm, NOS	Breast-3
21	Prostate Adenocarcinoma	Prostate-1
22	Mucosal Melanoma of the Vulva/Vagina	Vulva/Vagina-2
23	Hepatoblastoma	Liver-2
24	Glassy Cell Carcinoma of the Cervix	Cervix-4
25	Pancreatic Adenocarcinoma	Pancreas-1
26	Embryonal Tumor	CNS/Brain-1
27	Non-Seminomatous Germ Cell Tumor	Testis
28	Cutaneous Squamous Cell Carcinoma	Skin-1
29	Endometrial Carcinoma	Uterus-2
30	Adenosquamous Carcinoma of the Pancreas	Pancreas-2
31	Bladder Urothelial Carcinoma	Bladder/Urinary Tract-3
32	Ewing Sarcoma	Bone-1
33	Undifferentiated Pleomorphic Sarcoma/Malignant Fibrous Histiocytoma/High-Grade Spindle Cell Sarcoma	Soft Tissue-1

34	Liposarcoma	Soft Tissue-2
35	Anaplastic Thyroid Cancer	Thyroid-2
36	Mature B-Cell Neoplasms	Lymphoid-1
37	Esophagogastric Adenocarcinoma	Esophagus/Stomach-2
38	Mature T and NK Neoplasms	Lymphoid-2
39	Rhabdomyosarcoma	Soft Tissue-3
40	Small Bowel Cancer	Bowel-2
41	Non-Small Cell Lung Cancer	Lung-1
42	Uterine Sarcoma/Mesenchymal	Uterus-3
43	Merkel Cell Carcinoma	Skin-2
44	Adrenocortical Carcinoma	Adrenal Gland
45	Ovarian Epithelial Tumor	Ovary/Fallopian Tube-2
46	Osteosarcoma	Bone-2
47	Ocular Melanoma	Eye-2
48	Neuroblastoma	Peripheral Nervous System-1
49	Myeloproliferative Neoplasms	Myeloid-1
50	Melanoma	Skin-3
51	Hepatocellular Carcinoma	Liver-3
52	Well-Differentiated Thyroid Cancer	Thyroid-3
53	Medullary Thyroid Cancer	Thyroid-4
54	Nerve Sheath Tumor	Peripheral Nervous System-2
55	Lung Neuroendocrine Tumor	Lung-2
56	Diffuse Glioma	CNS/Brain-2
57	Acute Myeloid Leukemia	Myeloid-2
58	Pleural Mesothelioma	Pleura
59	B-Lymphoblastic Leukemia/Lymphoma	Lymphoid-3
60	Non-Cancerous	Non-Cancerous
61	Sarcoma, NOS	Soft Tissue-4
62	Renal Cell Carcinoma	Kidney-1
63	Ovarian Germ Cell Tumor	Ovary/Fallopian Tube-3
64	Chondrosarcoma	Bone-3
65	Epithelioid Sarcoma	Soft Tissue-5
66	Pancreatic Neuroendocrine Tumor	Pancreas-3
67	Meningothelial Tumor	CNS/Brain-3
68	Fibrosarcoma	Soft Tissue-6
69	T-Lymphoblastic Leukemia/Lymphoma	Lymphoid-4
70	Leiomyosarcoma	Soft Tissue-7
71	Prostate Small Cell Carcinoma	Prostate-2

72	Synovial Sarcoma	Soft Tissue-8
73	Hodgkin Lymphoma	Lymphoid-5
74	Rhabdoid Cancer	Kidney-2
75	Non-Hodgkin Lymphoma	Lymphoid-6
76	Myelodysplastic Syndromes	Myeloid-3

Additional file 1 Supplementary Table S2

Prognostic factors for Glioma in Chinese Glioma Genome Atlas (CGGA) 693 database: univariate and multivariate study of overall survival (OS)

	Univariate analysis		Multivariate analysis	
Variable	HR(95% CI)	*P*-value	HR(95% CI)	*P*-value
SMARCAL1 expression	1.139(1.091-1.189)	3.25E-09	1.086(1.019-1.157)	0.011
WHO grade	2.545 (1.846-3.508)	1.37E-38	2.510(1.670-3.771)	2.17E-09
Age	1.027(1.018-1.035)	7.87E-10	1.008(0.999-1.017)	0.080
IDH status	0.380(0.309-0.468)	5.64E-20	0.677(0.504-0.909)	0.010
1p/19q Codel	0.268(0.193-0.372)	3.02E-15	0.460(0.309-0.685)	0.0001
MGMT status	0.795(0.639-0.990)	0.041	0.894(0.703-1.157)	0.364

Additional file 1 Supplementary Table S3

The correlation between SMARCAL1 and various immune cells in UCEC			
Gene	Cell	Cor	P.value
SMARCAL1	Type 2 T helper cell	0.291118058	7.34674E-05
SMARCAL1	Effector memeory CD4 T cell	0.203606812	0.006117465
SMARCAL1	Memory B cell	0.120745931	0.106396536
SMARCAL1	Activated CD4 T cell	0.0370414	0.621539398
SMARCAL1	Gamma delta T cell	0.007660758	0.918704837
SMARCAL1	Regulatory T cell	0.004793375	0.949079942
SMARCAL1	Natural killer T cell	-0.000607017	0.99354738
SMARCAL1	Immature dendritic cell	-0.032522726	0.664714765
SMARCAL1	Immature B cell	-0.044320458	0.554675096
SMARCAL1	Natural killer cell	-0.055399029	0.460122354
SMARCAL1	Activated B cell	-0.069704048	0.352477593
SMARCAL1	CD56dim natural killer cell	-0.074476847	0.32040243
SMARCAL1	Neutrophil	-0.077833958	0.299012428
SMARCAL1	Type 1 T helper cell	-0.087559602	0.242485218
SMARCAL1	Effector memeory CD8 T cell	-0.103068367	0.168560711
SMARCAL1	T follicular helper cell	-0.109117958	0.14480485
SMARCAL1	Central memory CD4 T cell	-0.111819697	0.135055976
SMARCAL1	CD56bright natural killer cell	-0.121833417	0.10325919
SMARCAL1	Central memory CD8 T cell	-0.136982087	0.066704875
SMARCAL1	Type 17 T helper cell	-0.15867728	0.033374757
SMARCAL1	Mast cell	-0.186612399	0.012131384
SMARCAL1	Activated dendritic cell	-0.196432696	0.008220899
SMARCAL1	Plasmacytoid dendritic cell	-0.197793854	0.007778347
SMARCAL1	Activated CD8 T cell	-0.221373885	0.002821958
SMARCAL1	Macrophage	-0.22320831	0.002596325
SMARCAL1	MDSC	-0.23135571	0.001778989
SMARCAL1	Monocyte	-0.243584527	0.000984093
SMARCAL1	Eosinophil	-0.31220006	1.98513E-05

Additional file 1 Supplementary Table S4

The correlation between SMARCAL1 and Immune Checkpoint Genes in Glioma			
Gene1	Gene2	Cor	P.value
SMARCAL1	CD276	0.491690941	3.90E-42
SMARCAL1	NRP1	0.462209752	8.08E-37
SMARCAL1	CD27	0.452083239	4.15E-35
SMARCAL1	CD160	0.401026622	2.58E-27
SMARCAL1	TNFSF4	0.365394031	1.27E-22
SMARCAL1	CD274	0.305331502	6.05E-16
SMARCAL1	VTCN1	0.295132338	5.94E-15
SMARCAL1	IDO2	0.28924663	2.13E-14
SMARCAL1	TNFRSF9	0.288735768	2.38E-14
SMARCAL1	CD200	0.288167873	2.68E-14
SMARCAL1	LAG3	0.287757564	2.93E-14
SMARCAL1	CD44	0.272971284	6.25E-13
SMARCAL1	CD80	0.272563412	6.79E-13
SMARCAL1	ADORA2A	0.266256125	2.36E-12
SMARCAL1	LAIR1	0.262472044	4.92E-12
SMARCAL1	CTLA4	0.260868247	6.69E-12
SMARCAL1	CD40	0.256151533	1.63E-11
SMARCAL1	LGALS9	0.251724146	3.71E-11
SMARCAL1	TNFSF15	0.249670599	5.40E-11
SMARCAL1	HAVCR2	0.243699718	1.58E-10
SMARCAL1	CD200R1	0.242147519	2.08E-10
SMARCAL1	CD40LG	0.23892309	3.65E-10
SMARCAL1	CD86	0.238121442	4.19E-10
SMARCAL1	CD28	0.234665403	7.58E-10
SMARCAL1	ICOS	0.232405185	1.11E-09
SMARCAL1	CD48	0.223120431	5.14E-09
SMARCAL1	PDCD1	0.22055444	7.75E-09
SMARCAL1	TNFSF14	0.2170305	1.35E-08
SMARCAL1	CD244	0.205433225	7.92E-08
SMARCAL1	PDCD1LG2	0.201336357	1.44E-07
SMARCAL1	IDO1	0.195566233	3.29E-07
SMARCAL1	TNFRSF14	0.191571966	5.75E-07
SMARCAL1	BTLA	0.186136182	1.20E-06
SMARCAL1	TNFRSF4	0.180787553	2.44E-06
SMARCAL1	TIGIT	0.179411145	2.91E-06
SMARCAL1	ICOSLG	0.174462309	5.48E-06
SMARCAL1	TNFRSF18	0.172147893	7.31E-06
SMARCAL1	TNFRSF8	0.163959792	1.97E-05
SMARCAL1	TMIGD2	0.159747659	3.22E-05

SMARCAL1	TNFSF9	0.128509365	0.000848401
SMARCAL1	KIR3DL1	0.123284903	0.001375272
SMARCAL1	CD70	0.103944556	0.007042497
SMARCAL1	TNFSF18	0.097374467	0.011614268

The correlation between SMARCAL1 and Immune Checkpoint Genes in LUAD			
Gene1	Gene2	Cor	P.value
SMARCAL1	CD276	0.748718601	7.79E-94
SMARCAL1	TNFRSF4	0.474458519	2.52E-30
SMARCAL1	LGALS9	0.443002405	3.25E-26
SMARCAL1	TNFRSF14	0.440908596	5.90E-26
SMARCAL1	TNFRSF8	0.435156492	2.97E-25
SMARCAL1	NRP1	0.433359963	4.89E-25
SMARCAL1	CD200	0.422841822	8.52E-24
SMARCAL1	LAG3	0.412817595	1.18E-22
SMARCAL1	TNFSF4	0.40933661	2.89E-22
SMARCAL1	CD40	0.406896916	5.38E-22
SMARCAL1	CD86	0.396705149	6.78E-21
SMARCAL1	HAVCR2	0.390587872	2.98E-20
SMARCAL1	LAIR1	0.373591055	1.55E-18
SMARCAL1	TNFRSF18	0.369033456	4.31E-18
SMARCAL1	CD44	0.365989256	8.44E-18
SMARCAL1	CD274	0.364439916	1.19E-17
SMARCAL1	TNFRSF25	0.363730943	1.38E-17
SMARCAL1	PDCD1	0.362406331	1.85E-17
SMARCAL1	PDCD1LG2	0.359566186	3.41E-17
SMARCAL1	CD80	0.346661912	5.13E-16
SMARCAL1	TNFSF9	0.336184765	4.24E-15
SMARCAL1	ICOSLG	0.33024426	1.35E-14
SMARCAL1	CD70	0.329431647	1.58E-14
SMARCAL1	CD244	0.318609944	1.23E-13
SMARCAL1	CD160	0.315977708	1.99E-13
SMARCAL1	TIGIT	0.314333304	2.69E-13
SMARCAL1	ADORA2A	0.309869444	6.03E-13
SMARCAL1	TNFRSF9	0.304388254	1.60E-12
SMARCAL1	TNFSF15	0.30361018	1.83E-12
SMARCAL1	CD28	0.296138134	6.64E-12
SMARCAL1	IDO1	0.289184573	2.13E-11
SMARCAL1	TMIGD2	0.287935663	2.62E-11
SMARCAL1	CD27	0.284411238	4.66E-11
SMARCAL1	TNFSF18	0.282406481	6.45E-11
SMARCAL1	TNFSF14	0.277092569	1.50E-10

SMARCAL1	CTLA4	0.273749608	2.54E-10
SMARCAL1	ICOS	0.259847656	2.08E-09
SMARCAL1	CD200R1	0.255087769	4.15E-09
SMARCAL1	CD48	0.225506031	2.26E-07
SMARCAL1	BTNL2	0.218799885	5.19E-07
SMARCAL1	IDO2	0.20972054	1.54E-06
SMARCAL1	VTCN1	0.204613528	2.78E-06
SMARCAL1	KIR3DL1	0.200943876	4.21E-06
SMARCAL1	BTLA	0.148652191	0.000705642
SMARCAL1	CD40LG	0.133009816	0.002465554
SMARCAL1	HHLA2	0.0960371	0.029161462

The correlation between SMARCAL1 and Immune Checkpoint Genes in LIHC			
Gene1	Gene2	Cor	P.value
SMARCAL1	CD276	0.60929055	4.49E-39
SMARCAL1	NRP1	0.561383804	3.42E-32
SMARCAL1	TNFSF15	0.389182085	7.27E-15
SMARCAL1	CD200	0.383729254	1.84E-14
SMARCAL1	TNFSF4	0.381070688	2.87E-14
SMARCAL1	VTCN1	0.363619388	4.86E-13
SMARCAL1	CD200R1	0.354064037	2.13E-12
SMARCAL1	TNFRSF4	0.352969112	2.52E-12
SMARCAL1	CD80	0.352512278	2.70E-12
SMARCAL1	TNFSF18	0.352390431	2.75E-12
SMARCAL1	LGALS9	0.339908382	1.74E-11
SMARCAL1	CD86	0.330313152	6.81E-11
SMARCAL1	TNFRSF9	0.30922072	1.16E-09
SMARCAL1	HAVCR2	0.302579535	2.71E-09
SMARCAL1	LAIR1	0.291906751	1.01E-08
SMARCAL1	ICOSLG	0.291151143	1.11E-08
SMARCAL1	TNFRSF8	0.285800628	2.10E-08
SMARCAL1	CD48	0.259482513	4.02E-07
SMARCAL1	CD44	0.256823394	5.32E-07
SMARCAL1	CD27	0.24132877	2.57E-06
SMARCAL1	TNFSF9	0.238661132	3.34E-06
SMARCAL1	HHLA2	0.228756004	8.57E-06
SMARCAL1	CD160	0.227439489	9.68E-06
SMARCAL1	CD274	0.225071138	1.20E-05
SMARCAL1	TNFRSF18	0.223348551	1.41E-05
SMARCAL1	TIGIT	0.210554457	4.35E-05
SMARCAL1	TNFRSF14	0.210148342	4.51E-05
SMARCAL1	ICOS	0.202343422	8.68E-05

SMARCAL1	TNFRSF25	0.198417332	0.000119471
SMARCAL1	CD70	0.196140718	0.000143426
SMARCAL1	CD28	0.188946992	0.0002521
SMARCAL1	PDCD1LG2	0.181390948	0.00044606
SMARCAL1	CTLA4	0.179864793	0.000499206
SMARCAL1	PDCD1	0.168849306	0.001095553
SMARCAL1	IDO1	0.166977124	0.001246394
SMARCAL1	CD244	0.165454707	0.001382889
SMARCAL1	KIR3DL1	0.162146565	0.001728029
SMARCAL1	LAG3	0.156718133	0.00246876
SMARCAL1	CD40LG	0.156097065	0.002569806
SMARCAL1	BTNL2	0.151848849	0.003368237
SMARCAL1	BTLA	0.124926692	0.016058779
SMARCAL1	CD40	0.121336485	0.019394346
SMARCAL1	IDO2	-0.195502589	0.000150909

The correlation between SMARCAL1 and Immune Checkpoint Genes in KIRC			
Gene1	Gene2	Cor	P.value
SMARCAL1	CD276	0.633549083	3.71E-61
SMARCAL1	NRP1	0.582909837	7.92E-50
SMARCAL1	TNFSF4	0.550564556	1.43E-43
SMARCAL1	PDCD1LG2	0.543753825	2.44E-42
SMARCAL1	CD200	0.51961183	3.42E-38
SMARCAL1	CD86	0.504855243	8.13E-36
SMARCAL1	LAIR1	0.501482297	2.73E-35
SMARCAL1	CD40	0.495919998	1.96E-34
SMARCAL1	LGALS9	0.492859433	5.72E-34
SMARCAL1	CD48	0.489953613	1.56E-33
SMARCAL1	HAVCR2	0.460695538	2.32E-29
SMARCAL1	TNFRSF8	0.450744161	4.95E-28
SMARCAL1	CD244	0.441744533	7.24E-27
SMARCAL1	CD28	0.437291491	2.65E-26
SMARCAL1	TNFSF18	0.435047321	5.06E-26
SMARCAL1	CD40LG	0.413930463	1.77E-23
SMARCAL1	CD200R1	0.405048946	1.84E-22
SMARCAL1	CD274	0.383947108	3.64E-20
SMARCAL1	CD80	0.382491746	5.17E-20
SMARCAL1	TNFSF9	0.379585498	1.04E-19
SMARCAL1	CD27	0.369919108	9.96E-19
SMARCAL1	TNFSF15	0.35582975	2.36E-17
SMARCAL1	BTLA	0.354431317	3.20E-17
SMARCAL1	TIGIT	0.351486791	6.06E-17

SMARCAL1	ICOS	0.329921325	5.33E-15
SMARCAL1	HHLA2	0.325092106	1.38E-14
SMARCAL1	IDO1	0.319805275	3.86E-14
SMARCAL1	CD160	0.318193169	5.26E-14
SMARCAL1	TNFRSF9	0.309749295	2.57E-13
SMARCAL1	CD70	0.305270851	5.85E-13
SMARCAL1	TNFRSF4	0.291439142	6.78E-12
SMARCAL1	TNFRSF14	0.285208578	1.96E-11
SMARCAL1	CD44	0.280347916	4.41E-11
SMARCAL1	KIR3DL1	0.275295018	1.01E-10
SMARCAL1	TNFSF14	0.256537981	1.86E-09
SMARCAL1	PDCD1	0.255917624	2.04E-09
SMARCAL1	ADORA2A	0.253693904	2.84E-09
SMARCAL1	CTLA4	0.25267985	3.30E-09
SMARCAL1	ICOSLG	0.242666479	1.39E-08
SMARCAL1	LAG3	0.242013168	1.53E-08
SMARCAL1	TMIGD2	0.203239541	2.24E-06
SMARCAL1	BTNL2	0.174971762	4.88E-05
SMARCAL1	TNFRSF25	0.174195037	5.27E-05
SMARCAL1	TNFRSF18	0.105728183	0.014604448
SMARCAL1	IDO2	0.086357764	0.046286959

The correlation between SMARCAL1 and Immune Checkpoint Genes in UCEC			
Gene1	Gene2	Cor	P.value
SMARCAL1	CD276	0.689096227	5.64E-78
SMARCAL1	ICOSLG	0.433075613	2.53E-26
SMARCAL1	CD40	0.426299253	1.79E-25
SMARCAL1	PDCD1LG2	0.417796136	1.95E-24
SMARCAL1	TNFSF4	0.396046596	6.53E-22
SMARCAL1	CD274	0.388884934	4.04E-21
SMARCAL1	TNFRSF8	0.381345821	2.62E-20
SMARCAL1	TNFRSF25	0.371586353	2.75E-19
SMARCAL1	NRP1	0.365984785	1.02E-18
SMARCAL1	CD80	0.365911783	1.04E-18
SMARCAL1	HAVCR2	0.35803423	6.31E-18
SMARCAL1	CD86	0.338315072	4.64E-16
SMARCAL1	LAIR1	0.328492866	3.53E-15
SMARCAL1	CD200R1	0.317682272	3.03E-14
SMARCAL1	CD44	0.311823038	9.38E-14
SMARCAL1	TNFSF15	0.297689768	1.29E-12
SMARCAL1	VTCN1	0.294808964	2.17E-12

SMARCAL1	LAG3	0.293756937	2.61E-12
SMARCAL1	CD160	0.285147807	1.18E-11
SMARCAL1	LGALS9	0.276889605	4.76E-11
SMARCAL1	IDO1	0.273583986	8.22E-11
SMARCAL1	CD28	0.270355172	1.39E-10
SMARCAL1	ADORA2A	0.269236941	1.67E-10
SMARCAL1	TNFSF9	0.257148655	1.11E-09
SMARCAL1	TNFSF18	0.245926857	5.97E-09
SMARCAL1	TNFRSF4	0.243576642	8.40E-09
SMARCAL1	TNFRSF14	0.231611739	4.52E-08
SMARCAL1	TIGIT	0.226199955	9.40E-08
SMARCAL1	BTLA	0.20778782	9.92E-07
SMARCAL1	ICOS	0.203892438	1.59E-06
SMARCAL1	CD200	0.203738059	1.62E-06
SMARCAL1	TMIGD2	0.195510805	4.26E-06
SMARCAL1	IDO2	0.194192597	4.96E-06
SMARCAL1	BTNL2	0.189384389	8.53E-06
SMARCAL1	TNFRSF9	0.180565856	2.23E-05
SMARCAL1	TNFRSF18	0.180036812	2.36E-05
SMARCAL1	CD244	0.175330763	3.86E-05
SMARCAL1	KIR3DL1	0.143093799	0.000807696
SMARCAL1	CD70	0.122189207	0.004280735
SMARCAL1	CTLA4	0.117449474	0.006049306
SMARCAL1	CD40LG	0.110709604	0.009693845
SMARCAL1	PDCD1	0.106250571	0.013072867
SMARCAL1	CD27	0.095264595	0.026153286

Additional file 1 Supplementary Table S5

The Correlation between SMARCAL1 and Drugs Sensitivity in Glioma			
Gene	Drug	Cor	P.value
SMARCAL1	SNX-2112	-0.580091056	2.14484E-57
SMARCAL1	Ruxolitinib	-0.542691335	8.7578E-48
SMARCAL1	KIN001-244	-0.535519767	2.90425E-48
SMARCAL1	BIX02189	-0.481138788	2.38981E-32
SMARCAL1	KIN001-266	-0.471310295	2.31139E-37
SMARCAL1	YM201636	-0.459709904	2.09309E-35
SMARCAL1	Foretinib	-0.446380337	3.36599E-33
SMARCAL1	Tipifarnib	-0.428988172	3.60828E-28
SMARCAL1	GSK1904529A	-0.420530541	9.23705E-23
SMARCAL1	PD-0332991	-0.419172175	6.21275E-30
SMARCAL1	QL-XII-61	-0.409461121	2.63869E-20
SMARCAL1	5-Fluorouracil	-0.408836516	8.27884E-22
SMARCAL1	BMS-509744	-0.391696337	4.50434E-23
SMARCAL1	Sorafenib	-0.387464812	5.7725E-20
SMARCAL1	PFI-1	-0.386287573	8.27852E-20
SMARCAL1	Y-39983	-0.383656845	3.21889E-21
SMARCAL1	BHG712	-0.38338326	1.39365E-19
SMARCAL1	BX-912	-0.380233549	2.29178E-19
SMARCAL1	NPK76-II-72-1	-0.379216545	4.18261E-21
SMARCAL1	I-BET-762	-0.378130103	1.15841E-19
SMARCAL1	Vinorelbine	-0.377044367	4.29378E-24
SMARCAL1	XL-184	-0.376587954	9.54866E-21
SMARCAL1	CX-5461	-0.375556404	2.00249E-17
SMARCAL1	THZ-2-102-1	-0.371466362	2.21611E-17
SMARCAL1	QL-XI-92	-0.365666487	5.44547E-18
SMARCAL1	MPS-1-IN-1	-0.361479848	4.11201E-18
SMARCAL1	XMD13-2	-0.356602639	4.75045E-16
SMARCAL1	XMD11-85h	-0.347514382	4.76673E-14
SMARCAL1	CP466722	-0.346334941	1.06803E-19
SMARCAL1	Salubrinal	-0.340826312	3.29349E-16
SMARCAL1	XMD8-92	-0.339163429	3.42776E-16
SMARCAL1	Temsirolimus	-0.336112711	3.48911E-19
SMARCAL1	TL-1-85	-0.335347244	2.56884E-14
SMARCAL1	NG-25	-0.333935978	1.47075E-15
SMARCAL1	PF-562271	-0.319484817	4.60473E-15
SMARCAL1	YK 4-279	-0.316486267	4.47501E-17
SMARCAL1	CAY10603	-0.313158052	3.6438E-16
SMARCAL1	Masitinib	-0.309927472	4.31225E-15
SMARCAL1	AG-014699	-0.306698991	1.06122E-12

SMARCAL1	Pazopanib	-0.306566782	2.17513E-13
SMARCAL1	Gefitinib	0.345800086	2.96976E-20
SMARCAL1	Navitoclax	0.400157748	1.55127E-20

The Correlation between SMARCAL1 and Drugs Sensitivity in LUAD			
Gene	Drug	Cor	P.value
SMARCAL1	Navitoclax	0.519722459	4.7007E-23
SMARCAL1	SB 216763	0.426924156	6.86819E-22
SMARCAL1	XMD11-85h	-0.307327057	1.29073E-10
SMARCAL1	RO-3306	-0.308669557	7.47529E-13
SMARCAL1	Cetuximab	-0.320216454	3.05349E-13
SMARCAL1	AZD7762	-0.323046487	1.94123E-12
SMARCAL1	GW 441756	-0.324788196	4.41314E-13
SMARCAL1	AZ628	-0.332278066	1.92756E-10
SMARCAL1	Linifanib	-0.334407046	2.68603E-13
SMARCAL1	FH535	-0.338286359	4.57537E-15
SMARCAL1	BX-795	-0.343262644	2.06819E-15
SMARCAL1	XMD8-92	-0.34366228	1.47326E-14
SMARCAL1	MPS-1-IN-1	-0.350938408	8.30948E-15
SMARCAL1	JNJ-26854165	-0.35104713	5.63399E-16
SMARCAL1	Roscovitine	-0.351747587	5.97633E-16
SMARCAL1	PFI-1	-0.374337705	4.76965E-17
SMARCAL1	Embelin	-0.374680802	1.52544E-18
SMARCAL1	YK 4-279	-0.386883599	7.19163E-20
SMARCAL1	Bleomycin	-0.38826519	1.69614E-17
SMARCAL1	Doxorubicin	-0.390578928	2.22767E-19
SMARCAL1	QL-XII-61	-0.397930132	6.42042E-18
SMARCAL1	FMK	-0.411569795	1.34107E-20
SMARCAL1	FTI-277	-0.417796622	8.12405E-21
SMARCAL1	BAY 61-3606	-0.42194122	2.63939E-23
SMARCAL1	MLN4924	-0.471436543	1.40076E-23
SMARCAL1	Lisitinib	-0.472353477	1.35617E-29
SMARCAL1	QL-VIII-58	-0.476307545	2.76369E-29
SMARCAL1	AMG-706	-0.480309638	1.11666E-30
SMARCAL1	AKT inhibitor VIII	-0.489049316	6.50998E-29
SMARCAL1	HG-5-113-01	-0.502624429	1.1988E-31
SMARCAL1	Salubrinal	-0.549783154	2.01878E-39
SMARCAL1	Mitomycin C	-0.556140324	4.15273E-39

The Correlation between SMARCAL1 and Drugs Sensitivity in LIHC			
Gene	Drug	Cor	P.value
SMARCAL1	Sorafenib	-0.453149194	2.41326E-16
SMARCAL1	Lapatinib	0.346795834	1.67164E-08
SMARCAL1	Doxorubicin	-0.307339887	5.01854E-09
SMARCAL1	Vinorelbine	-0.31779343	4.19573E-10
SMARCAL1	AP-24534	-0.361834115	1.38399E-10
SMARCAL1	JNK-9L	-0.426573725	7.75219E-18
SMARCAL1	PF-562271	-0.417242123	2.47817E-15
SMARCAL1	HG-6-64-1	-0.30169977	5.761E-08
SMARCAL1	GSK-650394	-0.320508163	3.01492E-09
SMARCAL1	AUY922	-0.457966859	1.23653E-20
SMARCAL1	Epothilone B	-0.346387245	1.91516E-11
SMARCAL1	Tipifarnib	-0.504016398	3.05475E-23
SMARCAL1	AS601245	-0.321942685	2.15348E-10
SMARCAL1	Linifanib	-0.321708825	3.86927E-09
SMARCAL1	KIN001-266	-0.330395976	8.03398E-11
SMARCAL1	Cytarabine	-0.513202143	2.57895E-26
SMARCAL1	VX-702	0.309586157	3.90071E-07
SMARCAL1	KU-55933	0.338406861	5.52618E-09
SMARCAL1	GDC0449	0.323217381	2.44176E-07
SMARCAL1	RO-3306	-0.512224669	3.32056E-26
SMARCAL1	BEZ235	0.310879693	1.21387E-09
SMARCAL1	XMD11-85h	-0.331461316	6.0761E-09
SMARCAL1	QL-XII-61	-0.324208198	1.20159E-08
SMARCAL1	MLN4924	-0.53715489	4.05022E-29

The Correlation between SMARCAL1 and Drugs Sensitivity in KIRC			
Gene	Drug	Cor	P.value
SMARCAL1	Embelin	-0.70266681	1.59461E-80
SMARCAL1	Roscovitine	-0.62016741	5.35579E-55
SMARCAL1	BMS-509744	-0.613138929	1.92289E-45
SMARCAL1	Tipifarnib	-0.566570933	2.84641E-42
SMARCAL1	PHA-665752	-0.561013226	2.49364E-39
SMARCAL1	WH-4-023	-0.553872553	2.48375E-30
SMARCAL1	FTI-277	-0.516569672	4.96793E-34
SMARCAL1	Bexarotene	-0.516164385	5.49307E-35
SMARCAL1	Genentech Cpd 10	-0.500329093	1.6077E-31
SMARCAL1	Ruxolitinib	-0.491047273	1.83117E-31
SMARCAL1	CGP-60474	-0.486068194	5.91004E-33
SMARCAL1	GSK1904529A	-0.479451063	3.94874E-27

SMARCAL1	JNK-9L	-0.479362761	5.63334E-32
SMARCAL1	AP-24534	-0.475037078	3.1022E-23
SMARCAL1	HG-6-64-1	-0.47391519	2.67446E-27
SMARCAL1	TGX221	-0.472890039	1.01826E-30
SMARCAL1	5-Fluorouracil	-0.46444035	1.73631E-23
SMARCAL1	Pazopanib	-0.458958036	2.45277E-22
SMARCAL1	GNF-2	-0.456794527	4.83418E-25
SMARCAL1	Sorafenib	-0.454323705	1.83808E-21
SMARCAL1	Imatinib	-0.443806558	3.85147E-23
SMARCAL1	BAY 61-3606	-0.436253457	9.33061E-26
SMARCAL1	NG-25	-0.416305962	1.26604E-20
SMARCAL1	AS601245	-0.415048941	1.31014E-23
SMARCAL1	Epothilone B	-0.412644435	7.031E-23
SMARCAL1	TL-1-85	-0.411647395	6.80684E-19
SMARCAL1	Etoposide	-0.409841921	2.91015E-20
SMARCAL1	PF-562271	-0.404058952	6.93856E-20
SMARCAL1	Midostaurin	-0.403898566	5.44997E-18
SMARCAL1	LFM-A13	-0.39525682	7.46267E-21
SMARCAL1	Obatoclax Mesylate	-0.389320518	1.12328E-18
SMARCAL1	Bleomycin	-0.384703907	5.87206E-15
SMARCAL1	Salubrinal	-0.381624492	4.38691E-17
SMARCAL1	FH535	-0.378150731	8.12139E-19
SMARCAL1	AKT inhibitor VIII	-0.372866467	1.52637E-17
SMARCAL1	CMK	-0.365484792	2.80358E-17
SMARCAL1	Sunitinib	-0.363801933	2.8242E-14
SMARCAL1	BX-912	-0.363720997	2.30685E-14
SMARCAL1	GSK-650394	-0.356516131	7.31175E-16
SMARCAL1	A-770041	-0.339396127	7.34113E-12
SMARCAL1	LAQ824	-0.339143778	8.19924E-16
SMARCAL1	CP466722	-0.337828298	9.26331E-15
SMARCAL1	Gemcitabine	-0.333239619	3.28458E-13
SMARCAL1	FR-180204	-0.330286743	2.88127E-14
SMARCAL1	NPK76-II-72-1	-0.326119655	2.21322E-13
SMARCAL1	Lisitinib	-0.323211694	2.64594E-14
SMARCAL1	JW-7-52-1	-0.312778089	1.90349E-13
SMARCAL1	Parthenolide	-0.310940131	4.96573E-10
SMARCAL1	JQ12	-0.300350396	7.67938E-12
SMARCAL1	Phenformin	0.372850032	2.07463E-17

The Correlation between SMARCAL1 and Drugs Sensitivity in UCEC			
Gene	Drug	Cor	P.value
SMARCAL1	AKT inhibitor VIII	-0.765569705	8.39732E-94

SMARCAL1	A-443654	-0.752468888	1.5094E-100
SMARCAL1	Embelin	-0.744462709	1.88824E-96
SMARCAL1	JNK-9L	-0.716837374	4.31411E-87
SMARCAL1	BI-2536	-0.708782536	2.46908E-84
SMARCAL1	Paclitaxel	-0.697720977	1.0698E-80
SMARCAL1	BMS-509744	-0.683966777	6.7451E-64
SMARCAL1	HG-6-64-1	-0.680622999	2.15844E-75
SMARCAL1	Doxorubicin	-0.658225306	2.21985E-65
SMARCAL1	Vinorelbine	-0.657472992	9.22104E-69
SMARCAL1	CP466722	-0.656459426	9.51993E-68
SMARCAL1	LAQ824	-0.650809019	5.83591E-67
SMARCAL1	JQ12	-0.647908874	5.06262E-60
SMARCAL1	LFM-A13	-0.640320907	1.58789E-63
SMARCAL1	OSU-03012	-0.635463347	7.22779E-63
SMARCAL1	FH535	-0.635021586	1.61297E-61
SMARCAL1	Tipifarnib	-0.63493062	2.13766E-53
SMARCAL1	FR-180204	-0.626969703	1.77208E-58
SMARCAL1	GW843682X	-0.622505438	8.62471E-60
SMARCAL1	Sorafenib	-0.620428068	9.66387E-48
SMARCAL1	Bexarotene	-0.611182784	1.48369E-53
SMARCAL1	Epothilone B	-0.608379736	7.22155E-55
SMARCAL1	TL-1-85	-0.605612643	1.44379E-51
SMARCAL1	PF-562271	-0.602164291	8.64264E-49
SMARCAL1	Pazopanib	-0.601624719	1.01085E-51
SMARCAL1	Etoposide	-0.589929989	5.65624E-43
SMARCAL1	AS605240	-0.589084433	3.75142E-45
SMARCAL1	BX-912	-0.580062413	3.19831E-45
SMARCAL1	Genentech Cpd 10	-0.579586416	2.38566E-48
SMARCAL1	FTI-277	-0.575489648	9.86867E-45
SMARCAL1	BAY 61-3606	-0.571824102	2.69213E-48
SMARCAL1	NG-25	-0.567494381	4.60061E-45
SMARCAL1	AUY922	-0.566343722	1.50131E-47
SMARCAL1	PHA-665752	-0.565388815	2.45691E-44
SMARCAL1	AP-24534	-0.561657557	2.82271E-38
SMARCAL1	VX-680	-0.558192904	2.01788E-40
SMARCAL1	GSK-650394	-0.556140529	1.12877E-39
SMARCAL1	Imatinib	-0.555228499	1.59577E-39
SMARCAL1	GSK1904529A	-0.555069514	3.55538E-39
SMARCAL1	BMS345541	-0.549931448	2.12428E-44
SMARCAL1	5-Fluorouracil	-0.549929415	4.86401E-35
SMARCAL1	BMS-754807	-0.546452407	2.00693E-40
SMARCAL1	Ruxolitinib	-0.544548029	6.07909E-43
SMARCAL1	NPK76-II-72-1	-0.54061426	1.55284E-38

SMARCAL1	Midostaurin	-0.530660018	7.17945E-37
SMARCAL1	AT-7519	-0.529559864	1.42233E-39
SMARCAL1	Sunitinib	-0.529482948	9.17185E-38
SMARCAL1	Roscovitine	-0.526207418	2.21818E-37
SMARCAL1	Lisitinib	-0.523550362	1.77999E-39
SMARCAL1	Mitomycin C	-0.522547044	4.95379E-36
SMARCAL1	Salubrinal	-0.518525081	1.36273E-34
SMARCAL1	Obatoclax Mesylate	-0.515421025	8.54996E-34
SMARCAL1	VX-11e	-0.514414617	1.90621E-33
SMARCAL1	STF-62247	-0.508162152	6.66298E-35
SMARCAL1	PAC-1	-0.479663094	3.26166E-27
SMARCAL1	KIN001-135	-0.479380766	1.71048E-30
SMARCAL1	WZ3105	-0.477770372	3.52261E-31
SMARCAL1	GSK1070916	-0.467921213	2.37323E-26
SMARCAL1	QS11	-0.463178956	9.81785E-26
SMARCAL1	AS601245	-0.462815419	2.77686E-30
SMARCAL1	ZSTK474	-0.461734952	1.48414E-29
SMARCAL1	Tubastatin A	-0.446584297	3.18193E-22
SMARCAL1	Zibotentan	-0.433614729	2.40319E-26
SMARCAL1	Gemcitabine	-0.423964857	1.03859E-21
SMARCAL1	FMK	-0.41533264	1.31683E-21
SMARCAL1	Ispinesib Mesylate	-0.41255619	4.31232E-22
SMARCAL1	Bleomycin	-0.412376842	4.56532E-20
SMARCAL1	NSC-207895	-0.406861724	2.6103E-22
SMARCAL1	Shikonin	-0.401196777	2.67049E-22
SMARCAL1	XL-184	-0.397051999	8.48042E-22
SMARCAL1	CGP-60474	-0.395929523	6.73381E-22
SMARCAL1	CGP-082996	-0.39077853	8.4629E-20
SMARCAL1	Pyrimethamine	-0.389485535	6.04689E-19
SMARCAL1	WZ-1-84	-0.387455882	1.31724E-19
SMARCAL1	TAK-715	-0.36435578	1.73192E-17
SMARCAL1	IPA-3	-0.358323848	1.45251E-15
SMARCAL1	CMK	-0.353929545	2.07567E-17
SMARCAL1	WH-4-023	-0.347813341	2.88457E-15
SMARCAL1	DMOG	-0.34606837	1.92925E-16
SMARCAL1	CP724714	-0.330690992	2.23736E-14
SMARCAL1	Thapsigargin	-0.325378032	6.61654E-15
SMARCAL1	GNF-2	-0.314743939	2.36733E-13
SMARCAL1	Lapatinib	0.408372012	2.82076E-20

Full-length Blots/Gels

Figure12C

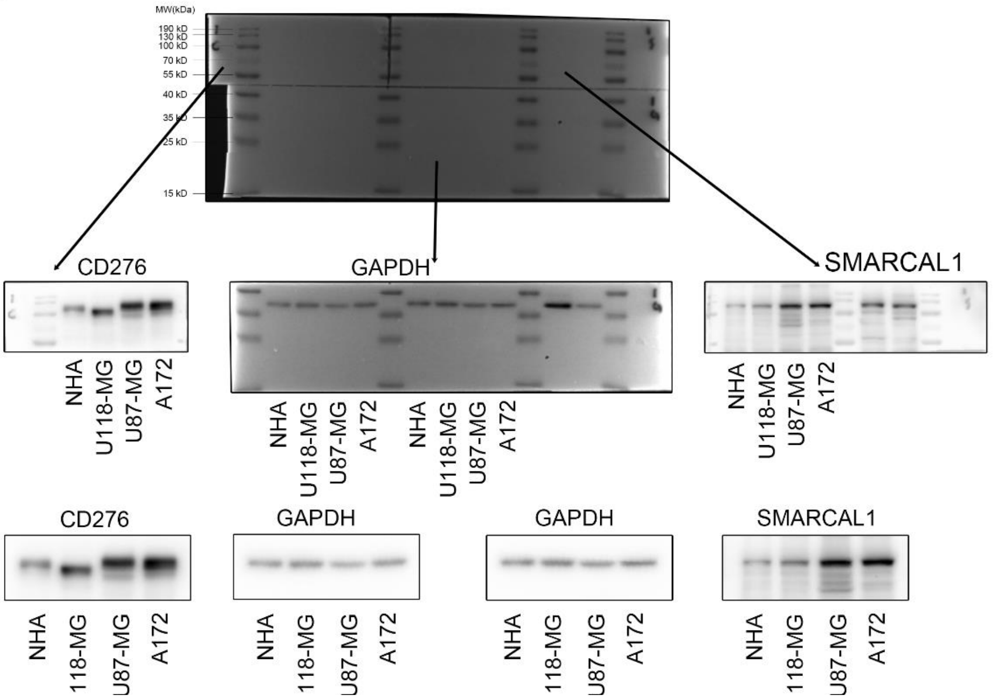

Figure 12D

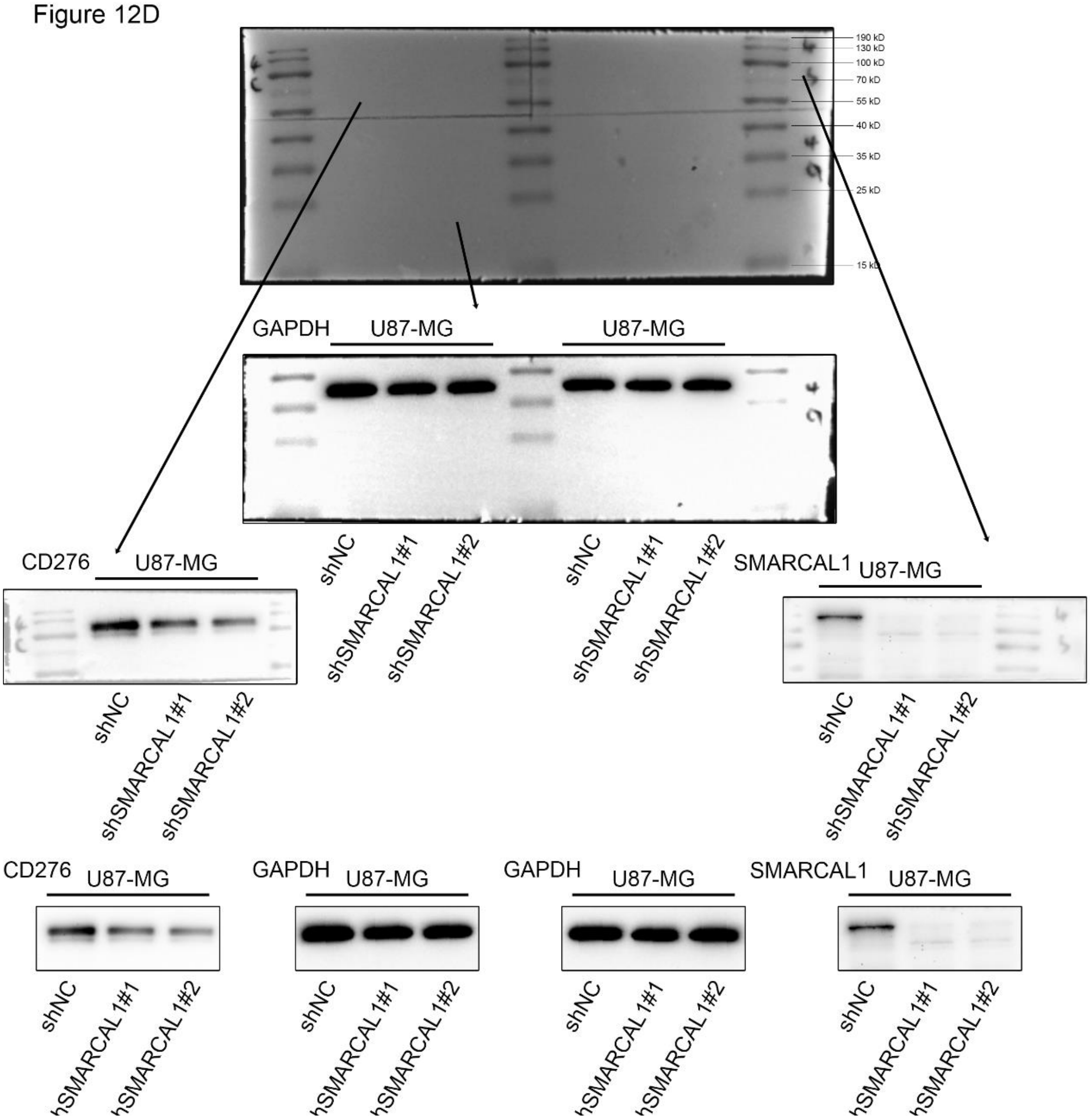
190 kD
130 kD
100 kD
70 kD
55 kD
40 kD
35 kD
25 kD
15 kD
GAPDH
U87-MG
U87-MG
shNC
shSMARCAL1#1
shSMARCAL1#2
shNC
shSMARCAL1#1
shSMARCAL1#2
CD276
U87-MG
shNC
shSMARCAL1#1
shSMARCAL1#2
SMARCAL1
U87-MG
shNC
shSMARCAL1#1
shSMARCAL1#2
CD276
U87-MG
shNC
GAPDH
U87-MG
shNC
GAPDH
U87-MG
shNC
SMARCAL1
U87-MG
shNC

Figure 12E

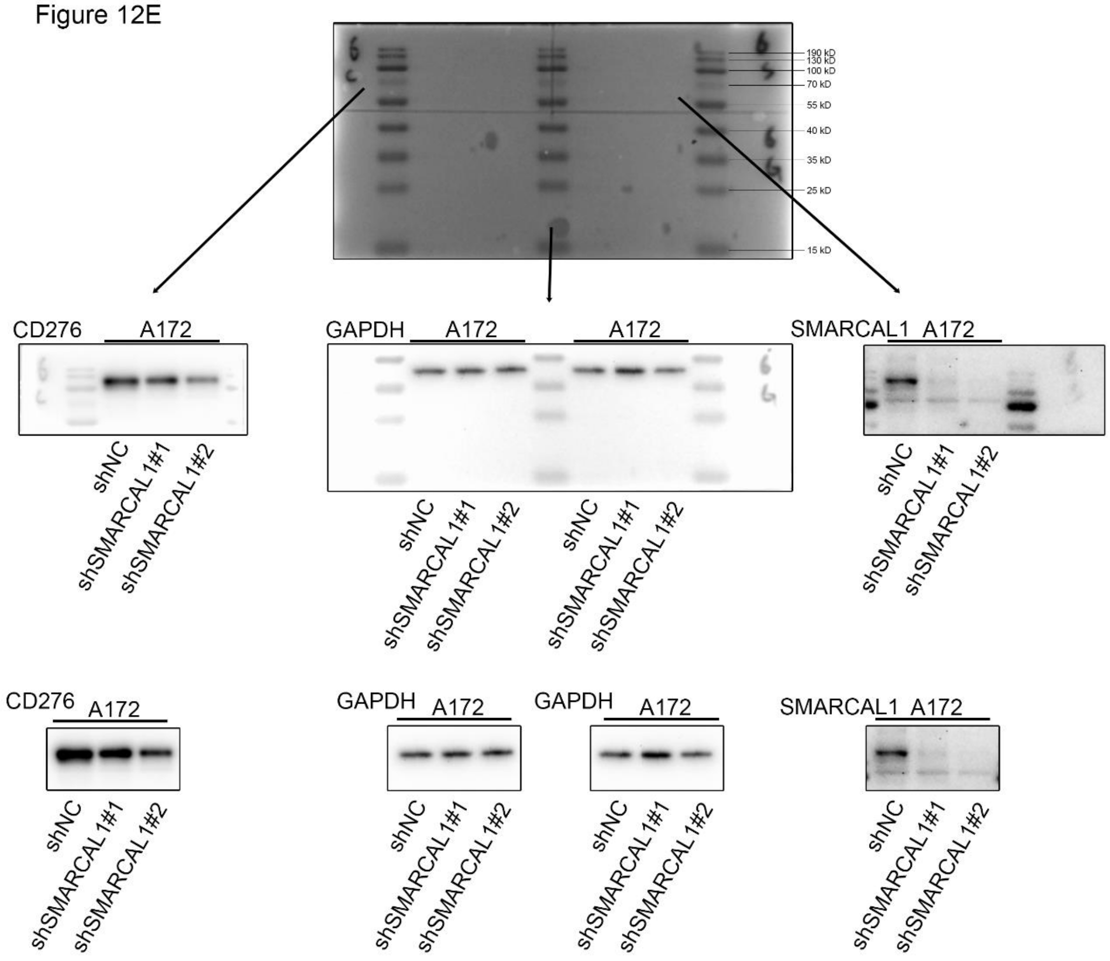
190 kD
130 kD
100 kD
70 kD
55 kD
40 kD
35 kD
25 kD
15 kD
CD276
A172
shNC
shSMARCAL1#1
shSMARCAL1#2
GAPDH
A172
A172
shNC
shSMARCAL1#1
shSMARCAL1#2
shNC
shSMARCAL1#1
shSMARCAL1#2
SMARCAL1
A172
shNC
shSMARCAL1#1
shSMARCAL1#2
CD276
A172
shNC
shSMARCAL1#1
shSMARCAL1#2
GAPDH
A172
shNC
shSMARCAL1#1
shSMARCAL1#2
GAPDH
A172
shNC
shSMARCAL1#1
shSMARCAL1#2
SMARCAL1
A172
shNC
shSMARCAL1#1
shSMARCAL1#2

Figure S11A

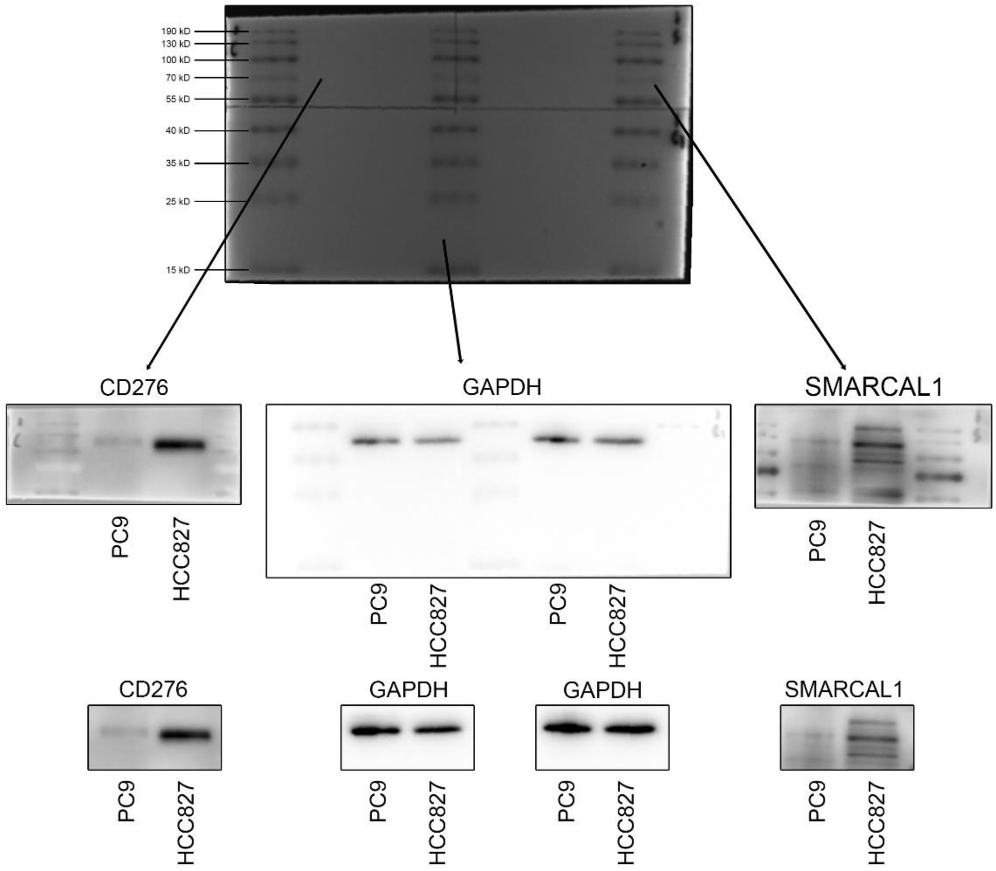
190 kD
130 kD
100 kD
70 kD
55 kD
40 kD
35 kD
25 kD
15 kD
CD276
GAPDH
SMARCAL1
PC9
HCC827
PC9
HCC827
PC9
HCC827
PC9
HCC827
CD276
GAPDH
GAPDH
SMARCAL1
PC9
HCC827
PC9
HCC827
PC9
HCC827
PC9
HCC827

Figure S11B

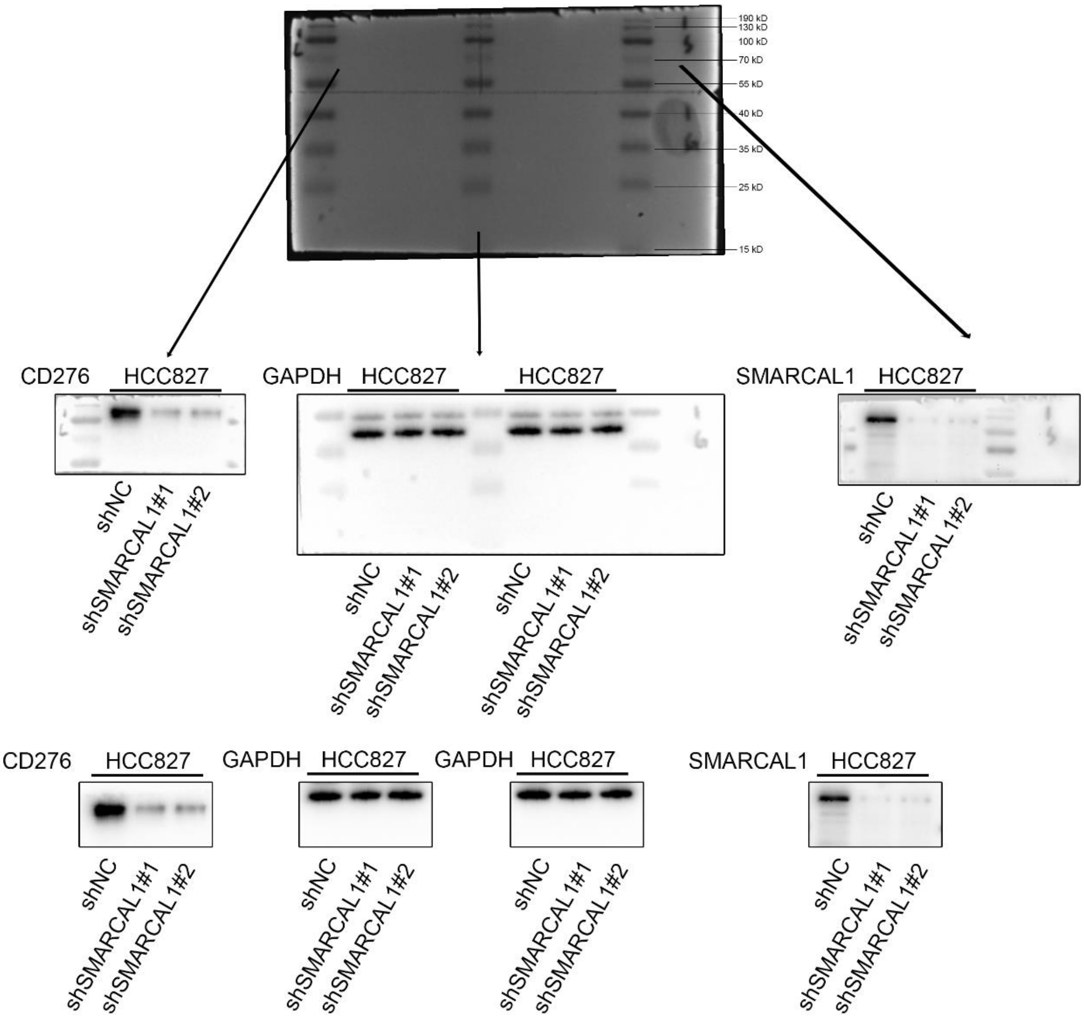
190 kD
130 kD
100 kD
70 kD
55 kD
40 kD
35 kD
25 kD
15 kD
CD276
HCC827
shNC
shSMARCAL1#1
shSMARCAL1#2
GAPDH
HCC827
HCC827
shNC
shSMARCAL1#1
shSMARCAL1#2
shNC
shSMARCAL1#1
shSMARCAL1#2
SMARCAL1
HCC827
shNC
shSMARCAL1#1
shSMARCAL1#2
CD276
HCC827
shNC
shSMARCAL1#1
shSMARCAL1#2
GAPDH
HCC827
shNC
shSMARCAL1#1
shSMARCAL1#2
GAPDH
HCC827
shNC
shSMARCAL1#1
shSMARCAL1#2
SMARCAL1
HCC827
shNC
shSMARCAL1#1
shSMARCAL1#2

Figure S11C

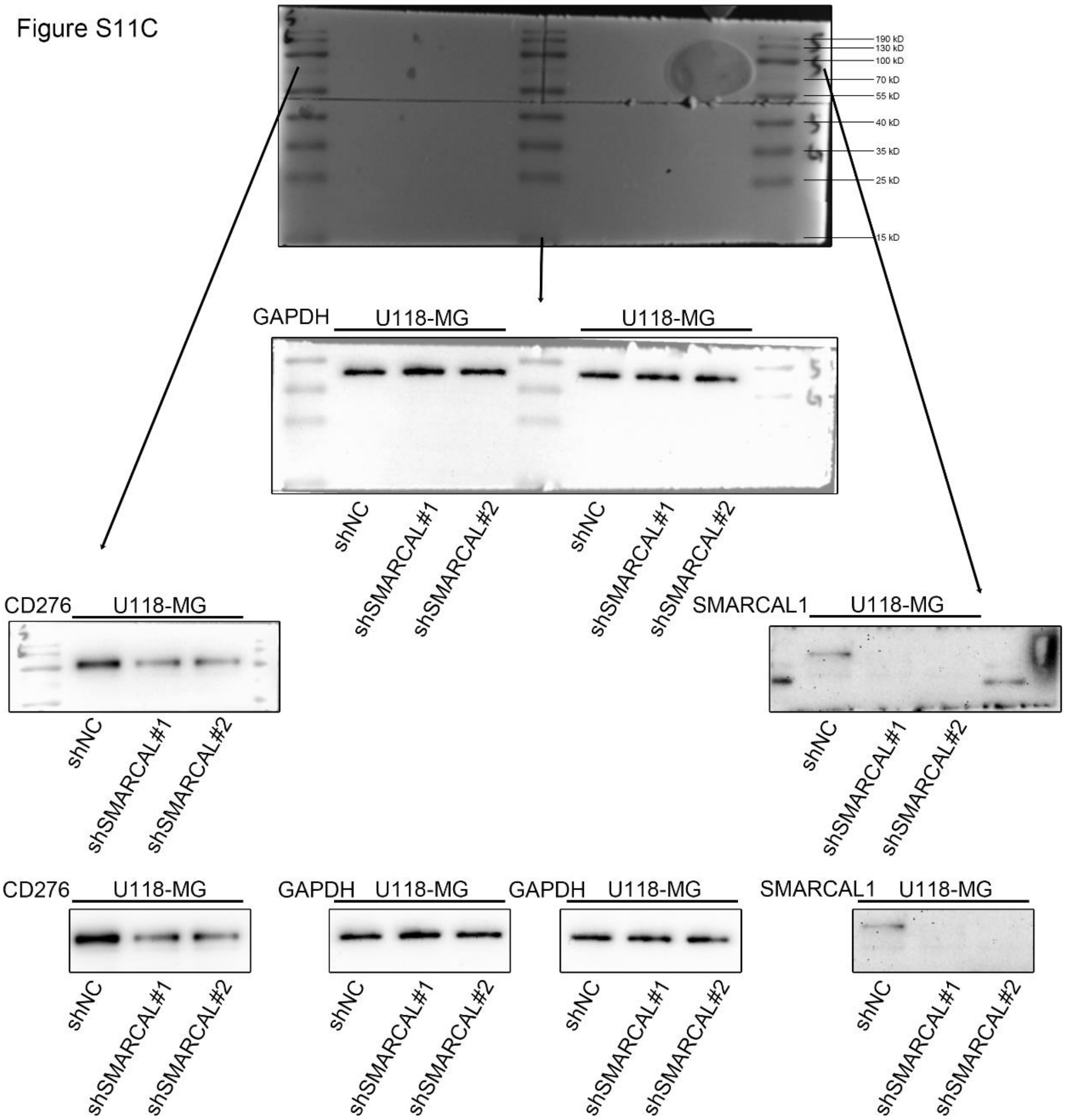

190 kD
130 kD
100 kD
70 kD
55 kD
40 kD
35 kD
25 kD
15 kD
GAPDH
U118-MG
U118-MG
shNC
shSMARCAL#1
shSMARCAL#2
shNC
shSMARCAL#1
shSMARCAL#2
CD276
U118-MG
shNC
shSMARCAL#1
shSMARCAL#2
SMARCAL1
U118-MG
shNC
shSMARCAL#1
shSMARCAL#2
CD276
U118-MG
shNC
shSMARCAL#1
shSMARCAL#2
GAPDH
U118-MG
shNC
shSMARCAL#1
shSMARCAL#2
GAPDH
U118-MG
shNC
shSMARCAL#1
shSMARCAL#2
SMARCAL1
U118-MG
shNC
shSMARCAL#1
shSMARCAL#2
