# Supplementary material for: Pan-cancer analysis reveals SMARCAL1 expression is associated with immune cell infiltration and poor prognosis in various cancers
Source: Sci Rep. 2025 Feb 24;15:6591. doi: 10.1038/s41598-025-88955-9 (PMC11850860; doi:10.1038/s41598-025-88955-9)
Supplement: Supplementary file 2 — Supplementary Information 2. [file 41598_2025_88955_MOESM2_ESM.pdf]

Unprocessed source data underlying all blots, Related to Figures 12 and S11

Figure12C

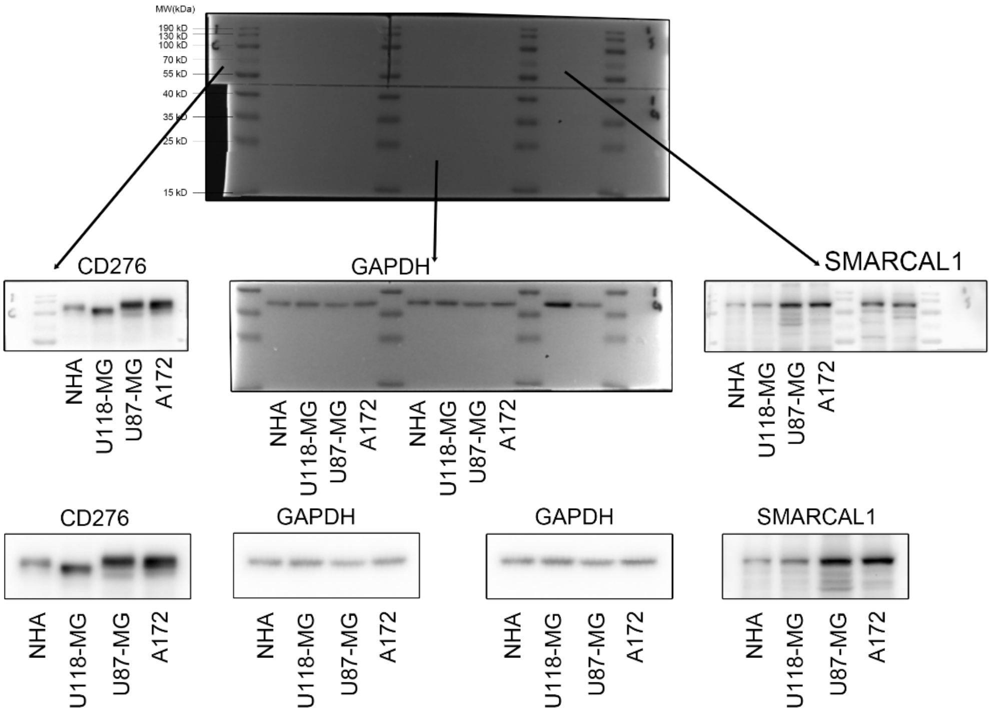

Figure 12D

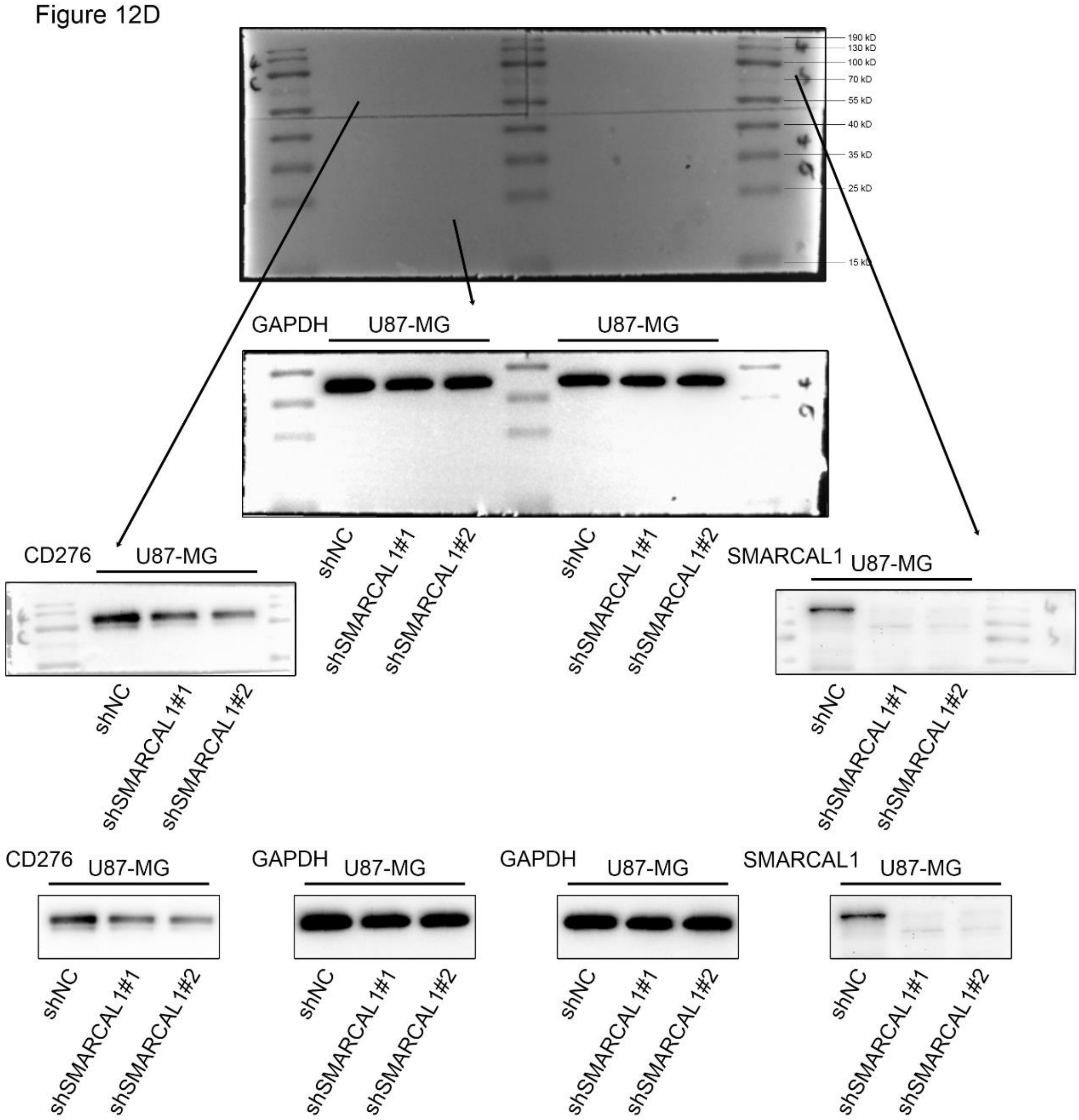
190 kD
130 kD
100 kD
70 kD
55 kD
40 kD
35 kD
25 kD
15 kD
GAPDH
U87-MG
U87-MG
shNC
shSMARCAL1#1
shSMARCAL1#2
shNC
shSMARCAL1#1
shSMARCAL1#2
CD276
U87-MG
shNC
shSMARCAL1#1
shSMARCAL1#2
SMARCAL1
U87-MG
shNC
shSMARCAL1#1
shSMARCAL1#2
CD276
U87-MG
shNC
shSMARCAL1#1
shSMARCAL1#2
GAPDH
U87-MG
shNC
shSMARCAL1#1
shSMARCAL1#2
GAPDH
U87-MG
shNC
shSMARCAL1#1
shSMARCAL1#2
SMARCAL1
U87-MG
shNC
shSMARCAL1#1
shSMARCAL1#2

Figure 12E

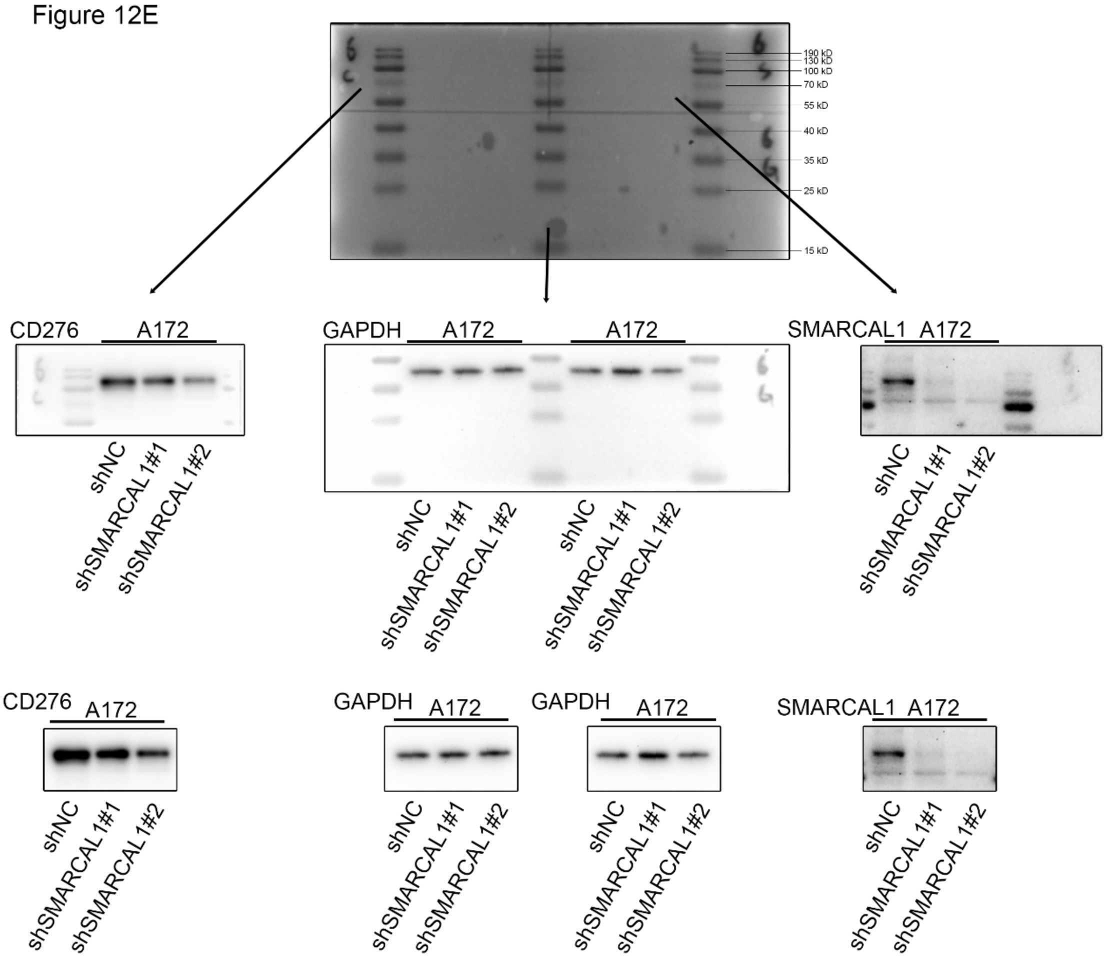

190 kD
130 kD
100 kD
70 kD
55 kD
40 kD
35 kD
25 kD
15 kD
CD276
A172
shNC
shSMARCAL1#1
shSMARCAL1#2
GAPDH
A172
A172
shNC
shSMARCAL1#1
shSMARCAL1#2
shNC
shSMARCAL1#1
shSMARCAL1#2
SMARCAL1
A172
shNC
shSMARCAL1#1
shSMARCAL1#2
CD276
A172
shNC
shSMARCAL1#1
shSMARCAL1#2
GAPDH
A172
shNC
shSMARCAL1#1
shSMARCAL1#2
GAPDH
A172
shNC
shSMARCAL1#1
shSMARCAL1#2
SMARCAL1
A172
shNC
shSMARCAL1#1
shSMARCAL1#2

Figure S11A

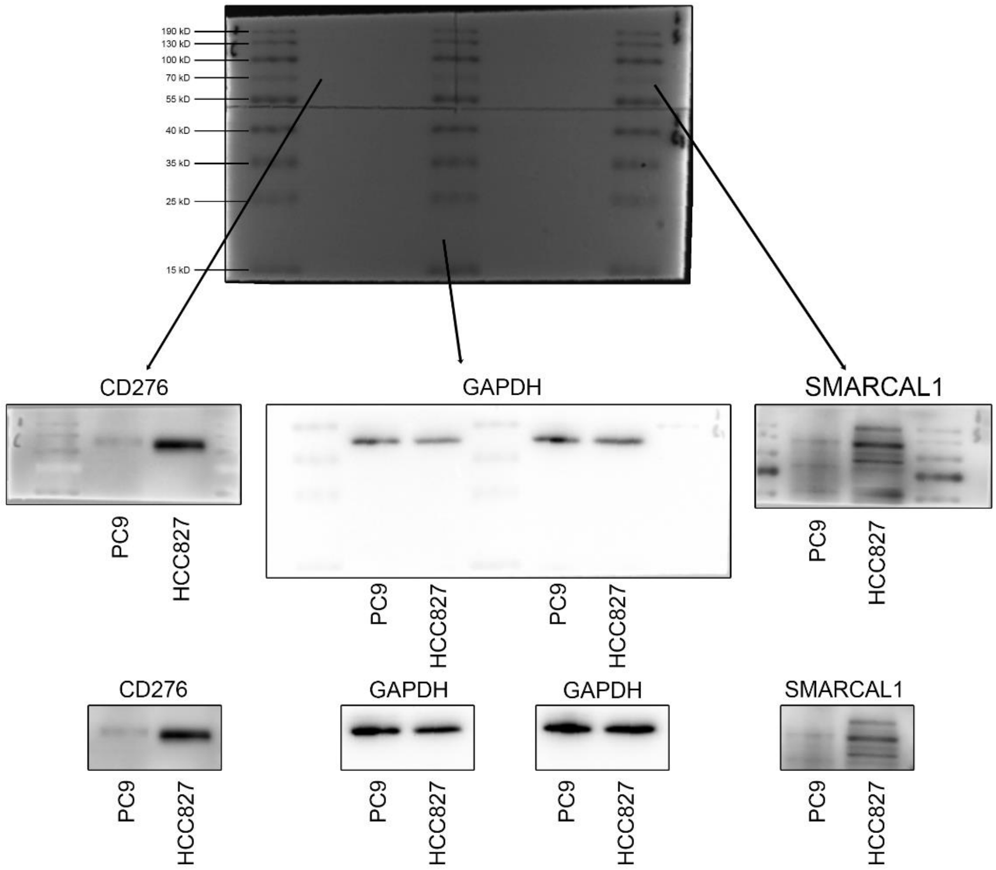
190 kD
130 kD
100 kD
70 kD
55 kD
40 kD
35 kD
25 kD
15 kD
CD276
GAPDH
SMARCAL1
PC9
HCC827
PC9
HCC827
PC9
HCC827
PC9
HCC827
CD276
GAPDH
GAPDH
SMARCAL1
PC9
HCC827
PC9
HCC827
PC9
HCC827
PC9
HCC827

Figure S11B

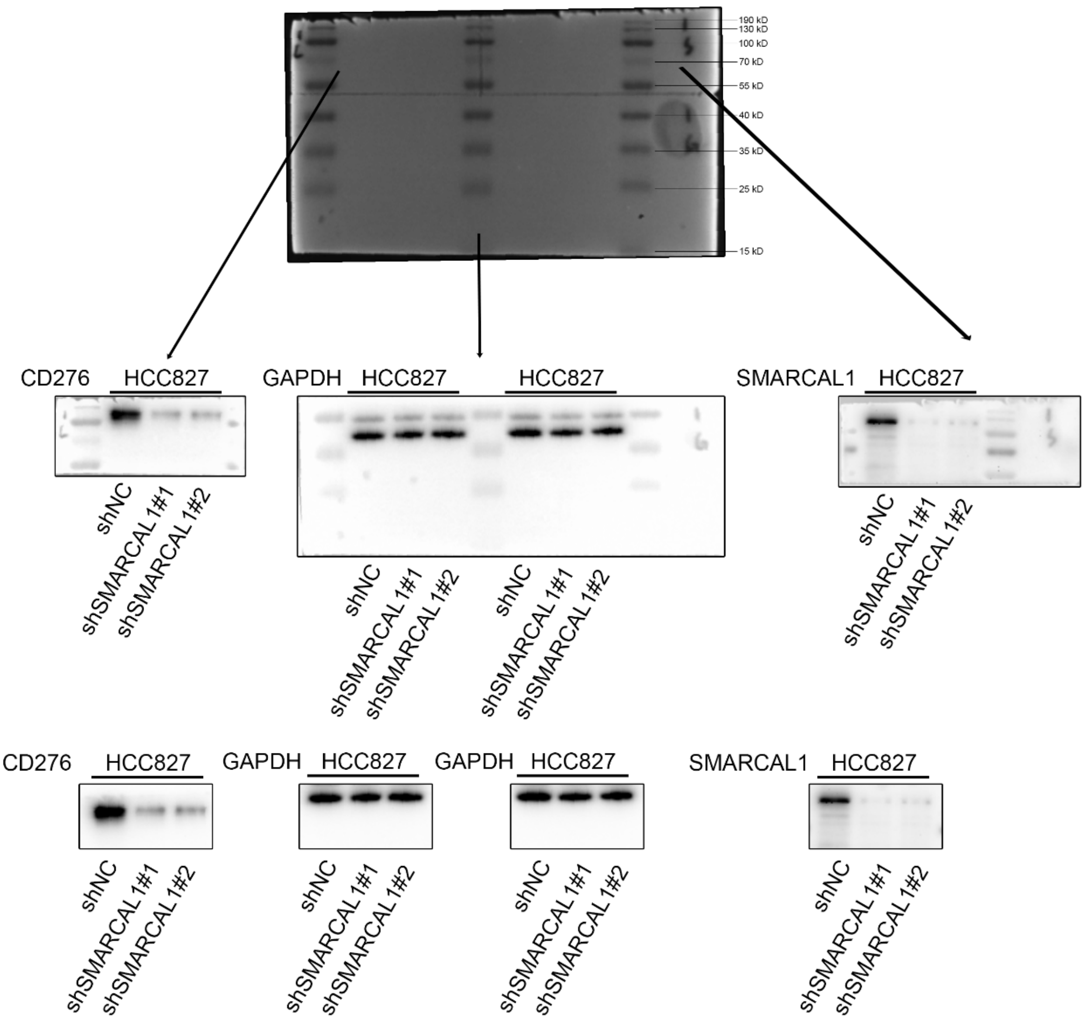
190 kD
130 kD
100 kD
70 kD
55 kD
40 kD
35 kD
25 kD
15 kD
CD276
HCC827
shNC
shSMARCAL1#1
shSMARCAL1#2
GAPDH
HCC827
HCC827
shNC
shSMARCAL1#1
shSMARCAL1#2
shNC
shSMARCAL1#1
shSMARCAL1#2
SMARCAL1
HCC827
shNC
shSMARCAL1#1
shSMARCAL1#2
CD276
HCC827
GAPDH
HCC827
GAPDH
HCC827
SMARCAL1
HCC827
shNC
shSMARCAL1#1
shSMARCAL1#2
shNC
shSMARCAL1#1
shSMARCAL1#2
shNC
shSMARCAL1#1
shSMARCAL1#2
shNC
shSMARCAL1#1
shSMARCAL1#2

Figure S11C

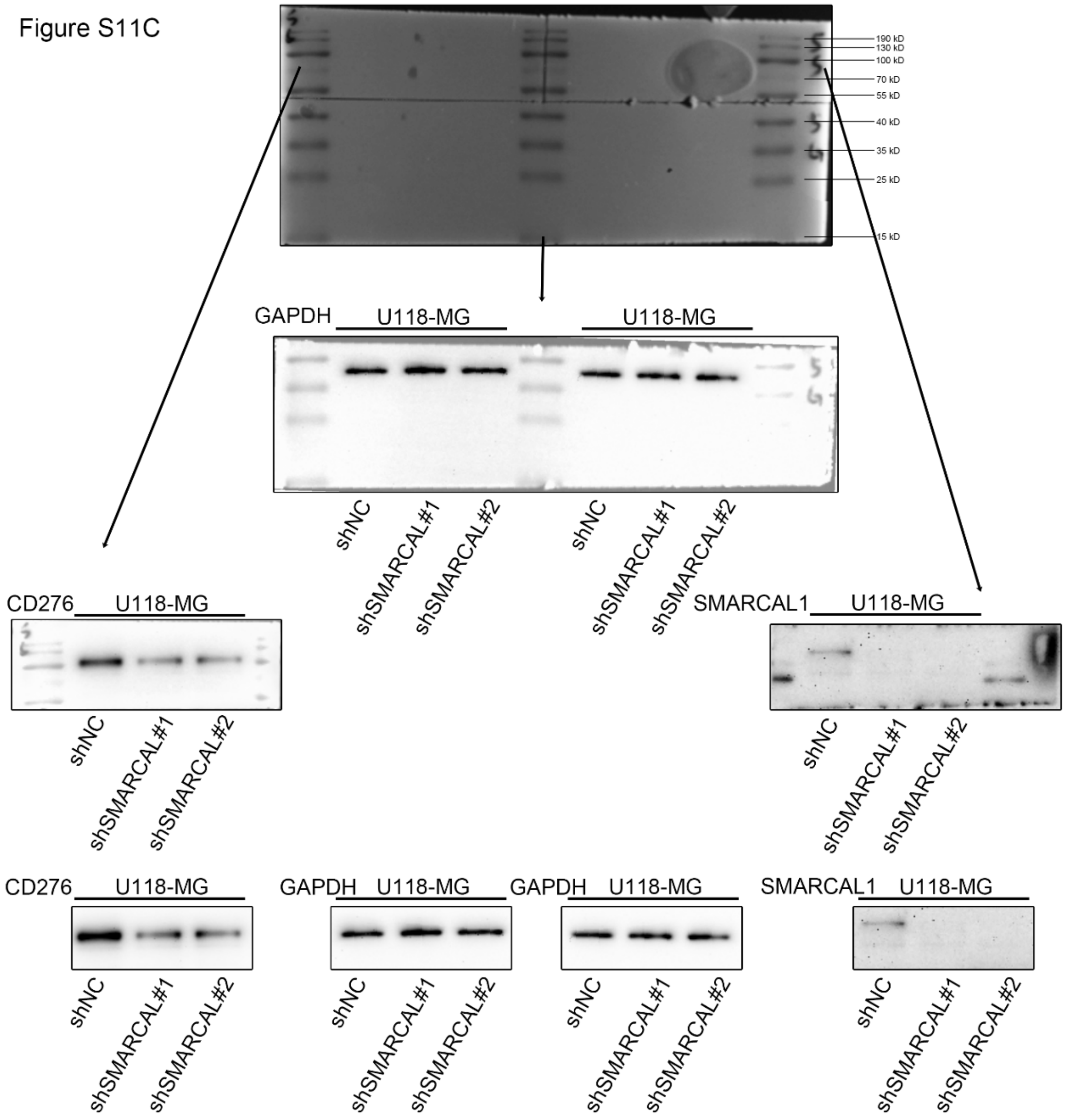

190 kD
130 kD
100 kD
70 kD
55 kD
40 kD
35 kD
25 kD
15 kD
GAPDH
U118-MG
U118-MG
shNC
shSMARCAL#1
shSMARCAL#2
shNC
shSMARCAL#1
shSMARCAL#2
CD276
U118-MG
shNC
shSMARCAL#1
shSMARCAL#2
SMARCAL1
U118-MG
shNC
shSMARCAL#1
shSMARCAL#2
CD276
U118-MG
shNC
shSMARCAL#1
shSMARCAL#2
GAPDH
U118-MG
shNC
shSMARCAL#1
shSMARCAL#2
GAPDH
U118-MG
shNC
shSMARCAL#1
shSMARCAL#2
SMARCAL1
U118-MG
shNC
shSMARCAL#1
shSMARCAL#2
